# Dynamically optimizing stomatal conductance for maximum turgor-driven growth over diel and seasonal cycles

**DOI:** 10.1093/aobpla/plad044

**Published:** 2023-07-06

**Authors:** Aaron Potkay, Xue Feng

**Affiliations:** Department of Civil, Environmental, and Geo-Engineering, University of Minnesota, Twin Cities, 500 Pillsbury Drive S.E., Minneapolis, MN 55455, USA; Saint Anthony Falls Laboratory, University of Minnesota, Twin Cities, 23rd Ave SE, Minneapolis, MN 55414, USA; Department of Civil, Environmental, and Geo-Engineering, University of Minnesota, Twin Cities, 500 Pillsbury Drive S.E., Minneapolis, MN 55455, USA; Saint Anthony Falls Laboratory, University of Minnesota, Twin Cities, 23rd Ave SE, Minneapolis, MN 55414, USA

**Keywords:** Dynamic optimality, non-structural carbohydrates, source-sink dynamics, stomatal optimization, turgor-driven expansion, tree growth, tree hydraulics

## Abstract

Stomata have recently been theorized to have evolved strategies that maximize turgor-driven growth over plants’ lifetimes, finding support through steady-state solutions in which gas exchange, carbohydrate storage and growth have all reached equilibrium. However, plants do not operate near steady state as plant responses and environmental forcings vary diurnally and seasonally. It remains unclear how gas exchange, carbohydrate storage and growth should be dynamically coordinated for stomata to maximize growth. We simulated the gas exchange, carbohydrate storage and growth that dynamically maximize growth diurnally and annually. Additionally, we test whether the growth-optimization hypothesis explains nocturnal stomatal opening, particularly through diel changes in temperature, carbohydrate storage and demand. Year-long dynamic simulations captured realistic diurnal and seasonal patterns in gas exchange as well as realistic seasonal patterns in carbohydrate storage and growth, improving upon unrealistic carbohydrate responses in steady-state simulations. Diurnal patterns of carbohydrate storage and growth in day-long simulations were hindered by faulty modelling assumptions of cyclic carbohydrate storage over an individual day and synchronization of the expansive and hardening phases of growth, respectively. The growth-optimization hypothesis cannot currently explain nocturnal stomatal opening unless employing corrective ‘fitness factors’ or reframing the theory in a probabilistic manner, in which stomata adopt an *inaccurate* statistical ‘memory’ of night-time temperature. The growth-optimization hypothesis suggests that diurnal and seasonal patterns of stomatal conductance are driven by a dynamic *carbon-use strategy* that seeks to maintain homeostasis of carbohydrate reserves.

## Teaser

Stomatal behaviour has long been interpreted as an eco-evolutionary optimization strategy that maximizes fitness. Various theories have postulated different proxies for fitness to explain stomatal conductance, traditionally expressing fitness as photosynthetic carbon assimilation with some hydraulic cost or constraint on the optimization, typically soil moisture. A recent growth-optimization hypothesis proposed carbohydrate- and turgor-limited whole-plant growth as a better proxy for fitness. We examine its predictions under dynamic conditions in which whole-plant carbohydrate storage and sink demand vary diurnally and seasonally. Our dynamic simulations suggest that stomatal behaviour is closely linked to sink demand and whole-plant carbohydrate storage, especially the latter, and seeks to balance photosynthetic carbon supply and sink demand, maintaining homeostasis of whole-plant carbohydrate storage. However, the growth-optimization hypothesis was unable to explain why stomata open at night without incorporating either empirical corrective factors or a probabilistic perspective.

## Introduction

**Figure 1.**
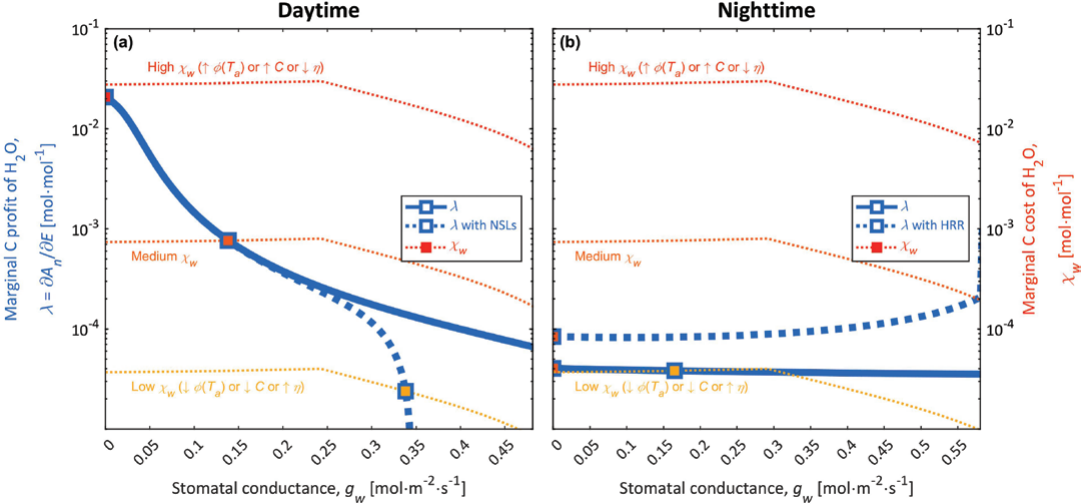
Daytime (**A**) and night-time (**B**) values of marginal carbon profit of water (*λ* = ∂*A*_n_/∂*E*; thick lines) and marginal carbon cost of water given by the growth-optimization hypothesis (GOH) (*χ*_w_; thin lines) for potential values of stomatal conductance (*g*_w_), where *A*_n_ and *E* are the leaf area-specific net carbon assimilation and transpiration rates, respectively [see Supporting Information—Fig. S1a and b]. Solid thick lines in **A** and **B** show the traditional daytime and night-time calculations of *λ*, in which gross photosynthetic assimilation depends on leaf internal CO_2_ concentration (*c*_*i*_) and leaf temperature (*T*_L_; see Supporting Information—Fig. S1c), and dark respiration similarly depends on *T*_L_. Dashed thick line in A and B show daytime *λ* with non-stomatal limitations (NSLs) and night-time *λ* with hydraulically regulated respiration (HRR), in which cases, gross photosynthetic assimilation and dark respiration decline with decreasing leaf water potential (*ψ*_L_) and leaf relative water content (RWC_L_; see Supporting Information—Fig. S1d), respectively. Markers denote the optimal *g*_w_. The range of the *x*-axis corresponds to values of RWC_L_ that exceed the turgor loss point [see Supporting Information—Fig. S1d]. In **A**, the daytime *λ* curve (without NSLs) and the Low *χ*_w_ curve intersect at a large *g*_w_ beyond turgor loss (*g*_w_ = 0.886 mol∙m^−2^∙s^−1^), corresponding to where *χ*_w_ = *λ* = 0. Simulations were performed at atmospheric CO_2_ and O_2_ partial pressures of 410 and 207 mmol∙mol^−1^, respectively, *T*_a_ = 25 °C, RH = 0.4, a solar elevation below the zenith of 0 radians, and incoming irradiance of 600 and 0 W∙m^−2^ in daytime and night-time simulations, respectively, using parameters from Potkay and Feng (2023) and Table 1. NSLs and HRR are turned on by default, and we turned them off for traditional estimates of *λ* by setting *φ*_c_ = *φ*_j_ = 1 [see Supporting Information—Equations S1.3.3 and S1.3.4] and *b*_r_^*^ = 0 [see Supporting Information—Equation S1.3.13].

It has long been theorized that stomata optimally use resources to maximize photosynthetic carbon assimilation (*A*_n_; see Table 1 for terminology; Cowan 1977; Cowan and Farquhar 1977). We refer to these and similar hypotheses that optimize *A*_n_ in relation to some metric of water stress (e.g. Wolf *et al.* 2016; Wang *et al.* 2020) as assimilation optimization hypotheses (AOHs). Assimilation optimization hypotheses predict the optimal stomatal conductance (*g*_w_) as the conductance at which the marginal carbon profit of water (*λ* = *dA*_n_/*dE*, where *E* is transpiration) and marginal carbon cost of water (*χ*_w_) are equal, unless the maximum *χ*_w_ exceeds the maximum *λ*, in which case *g*_w_ = 0 (Cowan and Farquhar 1977; Wang *et al.* 2020; Fig. 1), and stomata close as *λ* increases. *λ* is property of gas exchange and photosynthesis and is independent of optimization (Buckley *et al.* 2017; see Supporting Information—E**quation S1.3.10**), while *χ*_w_ emerges from finding the mathematical solution to the optimization problem given the assumed costs or constraints on the AOH (Wang *et al.* 2020; Potkay and Feng 2023; Equation 3). Estimates of *λ* are sensitive to how *A*_n_ and leaf dark respiration (*R*_d_) are modelled (Fig. 1; see Supporting Information—Fig. **S1**). The inclusion of non-stomatal limitations (NSLs) to photosynthesis (decreased mesophyll conductance and/or photosynthetic capacities; here modelled as reduced apparent photosynthetic capacities under negative leaf water potentials; Zhou *et al.* 2013; Novick *et al.* 2016; Dewar *et al.* 2018) causes daytime *λ* to decline faster with *g*_w_ than without NSLs (Fig. 1). For example, formulating *R*_d_ as a function of leaf relative water content (RWC_L_; Flexas *et al.* 2005; Galmés *et al.* 2007), here called hydraulically regulated respiration (HRR), nearly doubles night-time *λ* (when *A*_n_ = −*R*_d_; Fig. 1), since excessive transpiration dehydrates leaves, decreasing *R*_d_ (increasing *A*_n_; see Supporting Information—**Fig. S1**). When resources are scarce and needed, their costs (*χ*_w_) are high, leading to more conservative use (stomatal closure; Fig. 1). For example, stomata should not transpire a finite store of soil–water (e.g. sourced by episodic rain events) rapidly; otherwise, it depletes the soil–water available for future transpiration, reducing the total carbon gain through legacy effects (Buckley *et al.* 2017; Feng *et al.* 2022). The optimal strategy would be to transpire all the available water, depleting soil–water just as the next rain event either will imminently occur (Manzoni *et al.* 2013) or is probabilistically ‘anticipated’ (Cowan 1986; Mäkelä *et al.* 1996). This soil–water-saving strategy is often referred to as a *water-use strategy* (Manzoni *et al.* 2013; Mrad *et al.* 2019).

**Table 1.** Glossary of key variables and acronyms.

Symbol or acronym	Meaning
Variables	
* a*_L_	Leaf area
* A*_n_	Net carbon assimilation
* C*	Whole-plant NSC storage
* D*_L_	Vapour pressure deficit (VPD) between leaf and air
* E*	Transpiration
* f*_*c*_	Constant describing *R*_G_; *f*_c_ = *R*_G_/(*R*_G_ + *G*) < 1, and when *dC*/*dt* = 0, f_*c*_ = *R*_G_/(*a*_L_∙*A*_n_ − *R*_M_)
* f*_f_	‘Fitness factor’; *f*_f_ = *χ*_w,N_/*χ*_w,D_
* G*	Whole-tree growth
* I*_s_	Incoming irradiance on a surface normal to the solar beam
* R*_d_	Leaf dark respiration
* R*_G_	Construction respiration
RH	Relative humidity of air
* R*_M_	Stem and root maintenance respiration
RWC_L_	Leaf relative water content
* T*_L_	Leaf temperature
* η*	NSC-use efficiency (NSCUE); cost of depleting NSCs associated with the profit of growth; 0 < *η* ≤ (1 − *f*_*c*_)
* λ*	Marginal carbon profit of water; *λ* = *dA*_n_/*dE*
* * $\tilde{\phi }$	‘Effective’ extensibility, relating the average over the stem of the difference between turgor pressure and a threshold to the whole-stem relative volumetric growth rate
* χ*_w_	Marginal carbon cost of water
* ψ*_L_	Leaf water potential
Ф	Zenith angle; solar elevation below the zenith
Acronyms	
AOH	Assimilation optimization hypothesis; a hypothesis that states that stomata optimize a trade-off between *A*_n_ and some additional cost or constraint
eCO_2_	Elevated atmospheric CO_2_ concentration
GOH	Growth-optimization hypothesis; the hypothesis that stomata optimize turgor-driven growth
GOSM	Growth optimizing stomatal model; a model that predicts the *g*_w_ that maximizes turgor-driven growth; a GOSM makes other modelling assumptions in additional to the GOH to reach a tractable solution
HRR	Hydraulically regulated respiration; referring to the dependence of *R*_d_ on RWC_L_
NSC	Non-structural carbohydrate; large organic macromolecules that provide both the material and energy required for biological chemical reactions, the synthesis of other organic compounds and the growth of new biomass; NSCs buffer the asynchrony of supply and demand over diel, seasonal and decadal timescales and across plant organs
NSL	Non-stomatal limitation; referring to decreased mesophyll conductance and/or photosynthetic capacities; typically, relevant to leaf desiccation

D and N subscripts are used to denote daytime and night-time values, respectively.

Recently, Potkay and Feng (2023) theorized that stomata have evolved to maximize turgor-driven growth over entire lifetimes, which we refer to as the growth-optimization hypothesis (GOH). The GOH expands on AOHs by not only considering the optimal coordination between *g*_w_ and gas exchange but also non-structural carbohydrate (NSC) reserves (*C*) and turgor-driven growth (*G*). Though some AOHs are conceptually founded upon growth maximization, they mathematically couple growth to photosynthesis (treating *G* ∝ *A*_n_; Givnish and Vermeij 1976; Cowan 1982; Friend 1995), ignoring NSC storage and limitations. The GOH emphasizes carbon as a fundamental resource and NSCs as storage. The finite nature of NSCs imposes a cost to growth (represented by *η*; Equations 3 and 4). Spending NSCs to grow at one instance depletes the NSCs required for growth at later times, potentially hampering future growth as a legacy effect. Hence, stomata should maximize growth by storing NSCs when the potential for *C* accumulation is high and *η* is low (daytime, spring, summer) to be used when the potential for *C* accumulation is low and *η* is high (night-time, fall, winter, drought). Like the *water-use strategy*, the GOH suggests a *carbon-use strategy*, by which stomata open to slow the depletion of NSCs, especially when NSCs are valuable (small *C*, large *η*). NSC depletion is slowed in two ways. First, stomatal opening increases *A*_n_, accumulating NSCs. Second, stomatal opening reduces plant water potentials (*ψ*) (Sperry *et al.* 1998) and thus also cambial turgor (Hölttä *et al.* 2010), slows cell expansion and division (Lockhart 1965; Kirkham *et al.* 1972) and reduces growth’s carbon demand. For systems with multiple limiting resources, when one resource becomes more limiting, the other resources become less costly (Farquhar *et al.* 2002). All else being equal, the cost of water (*χ*_w_) should decline and stomata should open under low NSCs and/or high demand (low *C* and/or high *η* in Fig. 1). This coordination explains observations of more aggressive water use and accelerated declines in *ψ* and water content in NSC-depleted plants (O’Brien *et al.* 2014; Sapes *et al.* 2019) and in defoliated trees (Salmon *et al.* 2015, which stored fewer stem NSCs; Poyatos *et al.* 2013) as well as heightened stomatal sensitivity to elevated CO_2_ concentrations (eCO_2_) at the beginning of the growing season when NSC demands are high (Quentin *et al.* 2015; Urban *et al.* 2019; Sanches *et al.* 2020; Lauriks *et al.* 2021*b*, 2022). Alternatively, stomata should close, deprioritizing carbon gain, even when water is available, if growth is sink limited (Blonder *et al.* 2023).

Potkay and Feng (2023) showed that the GOH captures realistic stomatal, growth and carbohydrate responses to environmental cues in steady state (when stomata, NSCs and their costs have equilibrated). However, steady state reflects perfect acclimation of gas exchange, carbon use and growth to constant environmental conditions, and ignores the temporal dynamics of the underlying feedbacks between *g*_w_, *C* and *G* that define the *carbon-use strategy*, instead describing their final equilibrium. Real environmental conditions vary over diel, seasonal and longer timescales, and similarly, *g*_w_, NSCs and growth follow distinct temporal patterns over these timescales (Smith and Stitt 2007; Maseyk *et al.* 2008; Rossi *et al.* 2013; Martínez-Vilalta *et al.* 2016; Mencuccini *et al.* 2017; Tixier *et al.* 2018, 2020). Indeed, NSCs lag steady-state NSC predictions, and the lag depends on the amplitude and mean of seasonal photosynthetic activity, the phenology of NSC demand and tree size (Oswald and Aubrey 2023). Here, we present transient optimal simulations explicitly considering the *carbon-use strategy* under varying environmental conditions.

Additionally, we interpret the existence and environmental responses of nocturnal stomatal movements through the GOH. Stomata typically close during the first few hours of night and then open, reaching a maximum nocturnal conductance just before dawn (Hennessey *et al.* 1993; Resco de Dios *et al.* 2013*a*, 2015) with nocturnal stomatal conductances (*g*_w,N_) that are typically 5–40 % of daytime conductances (Caird *et al.* 2007). AOH models have been unable to simulate this nocturnal pattern (Zeppel *et al.* 2014; Yu *et al.* 2019), because their mathematical formulations for *χ*_w_ exceed night-time *λ* (Wang *et al.* 2021; Fig. 1), predicting complete closure at night. Parenthetically, representing HRR lessens this disparity between *χ*_w_ and *λ* (Fig. 1B). Wang *et al.* (2021) introduced an empirical ‘fitness factor’ (*f*_f_ = *χ*_w,N_/*χ*_w,D_, where *χ*_w,N_ and *χ*_w,D_ are night-time and daytime *χ*_w_) to predict nocturnal stomatal opening from *χ*_w,D_ (*χ*_w,N_ = *f*_f_∙*χ*_w,D_), where *χ*_w,D_ was predicted by Wang *et al.*’s (2020) AOH, and *f*_f_ ≈ 0.15 at 25 °C when ignoring NSLs and HRR. Their pragmatic approach cannot explain why *f*_f_ < 1 or how *f*_f_ should respond to environmental conditions. Justifying nocturnal stomata opening as optimal requires a theory for why night-time carbon costs of water are less than that of daytime (*χ*_w,N_ < *χ*_w,D_, *f*_f_ < 1).

The GOH suggests three hypotheses for why *χ*_w_ declines from day to night. First, *χ*_w_ scales with extensibility ($\tilde{\phi }$, which relates turgor pressure to relative volumetric growth; Lockhart 1965; Potkay *et al.* 2022), which is temperature dependent (Cabon *et al.* 2020; Peters *et al.* 2021), resulting from temperature dependent enzymatically regulated cell expansion (Cosgrove 2000) and following a modified-Eyring equation with a temperature optimum (Johnson *et al.* 1942; Parent *et al.* 2010; see Supporting Information—**Fig.** S1 in Potkay and Feng 2023). Since nights are cooler than days, $\tilde{\phi }$ and *χ*_w_ should be smaller at night than day, potentially opening stomata (Fig. 1B). Second, *χ*_w_ increases asymptotically with *C* (Potkay and Feng 2023), and thus *χ*_w_ should decline from day to night as NSCs wane (Smith and Stitt 2007; Tixier *et al.* 2018; Fig. 1B). Third, *χ*_w_ declines under high sink demand (*η*) and thus should decline at night when most volumetric growth typically occurs (Steppe *et al.* 2015; Zweifel *et al.* 2021; but see Mencuccini *et al.* 2017). Here, we test these three hypotheses for nocturnal stomatal opening.

## Materials and Methods

### Growth optimizing stomata model

Here, we review Potkay and Feng’s (2023) growth optimizing stomatal model (GOSM). The GOH states stomata maximize an individual tree’s whole-stem growth over its lifetime:


$$\begin{array}{l} \underset{g_{w}}{\max}\;\underset{t_{1}}{\overset{t_{2}}{\int }}\;G\left(C,\;E\right)\;dt, \end{array}$$ (1)

where *g*_w_ is the stomatal conductance to vapour, *t* is time and *G* is the whole-tree growth, which depends on the leaf area-specific transpiration (*E*) and the NSC storage (*C*) as control and state variables, respectively. This maximization is subject to the constraint that *C* must be balanced by its supply and demand:


$$\begin{array}{l} \dot{C}=\frac{dC}{dt}=a_{L}A_{n}-R_{M}-R_{G}-G=a_{L}A_{n}-R_{M}-\frac{G}{1-f_{c}}, \end{array}$$ (2)

where *a*_L_ is the leaf area, *A*_n_ is the leaf area-specific net assimilation, *R*_*M*_ is the stem and root maintenance respiration, *R*_*G*_ is the construction respiration and *f*_*c*_ is the constant fraction of *G* + *R*_*G*_ diverted to *R*_*G*_. Environmental conditions include the soil–water potential (*ψ*_soil_), air temperature (*T*_a_), the air’s relative humidity (RH), irradiance on a surface normal to the solar beam (*I*_s_) and solar elevation below the zenith (Φ). The difference in the temporal bounds over which growth is maximized (*t*_2_ − *t*_1_) is the *timescale of optimization*. The GOH suggests that the *timescale of optimization* spans an individual’s lifetime. Pragmatically, we approximate an infinitely long *timescale* by reducing it to its smallest repeated units, solving Equation (1) over a cyclic annual period (Oswald and Aubrey 2023). Since how *G* responds to *C* and *E* is sensitive to tree size (Potkay *et al.* 2022; Potkay and Feng 2023), this cyclic approximation holds for mature trees, whose relative growth is slow and whose relative change in size is negligible (Valentine and Mäkelä 2012; Forrester 2021), but not young trees, whose fast growth dynamically alters the relationship between *G*, *C* and *E*. Our boundary condition’s inability to describe small trees is an important limitation, considering that the GOH maximizes growth over a tree’s entire lifetime, including trees’ youngest stages. We propose alternative simulations for young trees in Supporting Information—**Notes S1 and Section S2**.

Solving Equations (1) and (2) through the calculus of variations (Witelski and Bowen 2015) with *g*_w_ as the control variable and *C* as the state variable, Potkay and Feng (2023) derived a solution through the marginal carbon cost of water (*χ*_w_; Fig. 1A),


$$\begin{array}{l} \chi_{w}=\left(\frac{1}{\eta }-\frac{1}{1-f_{c}}\right)\frac{1}{a_{L}}\left|\frac{\partial G}{\partial E}\right|, \end{array}$$ (3)

where *η* is the NSC-use efficiency, representing sink demand and the cost to growth of depleting NSCs (0 < *η* ≤ 1 − *f*_*c*_), and ∂*G*/∂*E* < 0, since transpiration causes *ψ* and turgor to decline, slowing growth. Their solution also describes how *η* changes in time,


$$\begin{array}{l} \dot{\eta }=\frac{d\eta }{dt}=\eta \frac{\partial R_{M}}{\partial C}-\left(1-\frac{\eta }{1-f_{c}}\right)\frac{\partial G}{\partial C}, \end{array}$$ (4)

For the full derivation of Equations (3) and (4), please see Potkay and Feng (2023; notably see **Section S2** of their Supporting Information). Though Potkay and Feng (2023) recognized that *η* is dynamic, they presented solutions in which *η* was either a known, instantaneous constant or in steady state ($\dot{\eta }\;\;=0$) for simplicity. Here, we describe the full dynamics of *η* as described by Equation (4).

**Figure 2.**
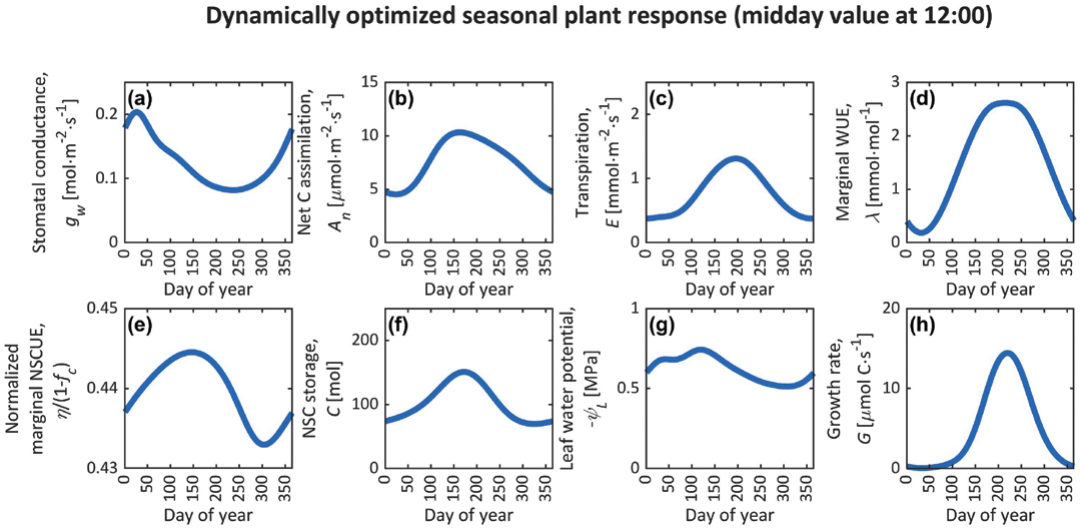
Annual trends from our year-long simulation in stomatal conductance (*g*_w_), photosynthetic carbon assimilation (*A*_n_), transpiration (*E*), marginal water-use efficiency (*λ*), NSC storage (*C*), growth rate (*G*), leaf water potentials (*ψ*_L_) and marginal NSC-use efficiency (NSCUE; *η*) normalized by its maximum value (1 − *f*_*c*_) that maximize growth under the cyclic environmental conditions shown in Supporting Information—Fig. S3. We present daily values at noon (1200 h).

The *carbon-use strategy* that opens stomata to conserve NSC is apparent from Equations (3) and (4). By Equation (4), the *η*∙∂*R*_M_/∂*C* term is responsible for elevating *η* over time when *C* is small, since *η*∙∂*R*_M_/∂*C* is always positive and greatest at low *C* (∂*R*_M_/∂*C* ≥ 0, ∂^2^*R*_M_/∂*C*^2^ ≤ 0). Meanwhile, the negative −(1 − *η*/(1 − *f*_c_))∙∂*G*/∂*C* term is responsible for diminishing *η* over time when either *C* is small or conditions are favourable for growth, either of which causes large ∂*G*/∂*C* (∂*G*/∂*C* ≥ 0, ∂^2^*G*/∂*C*^2^ ≤ 0, ∂*G*/∂*C* ∝ *G*). Their net effect of *C* on *η* is unclear from Equation (4) without *a priori* knowledge of the magnitudes of ∂*R*_M_/∂*C* and ∂*G*/∂*C*. Nonetheless, we later show $\dot{\eta }>0$ when *C* is small and $\dot{\eta }<0$ when *C* is large (Fig. 2). Hence, *C* and *η* feedback dynamically to stabilize each other. Large *η* opens stomata (smaller *χ*_w_ by Equation 3; Fig. 1A), assimilating more carbon while reducing sink demand, accumulating NSCs and reducing *η* over time by Equation (4). This negative feedback suggests that the *carbon-use strategy* seeks to maintain homeostasis of NSCs, which is indeed supported by past studies (Smith and Stitt 2007; Schönbeck *et al.* 2018; Dickman *et al.* 2019).

In Potkay and Feng’s (2023) GOSM, *A*_n_ is modelled by a big-leaf version of the Farquhar *et al.* (1980) model with temperature-dependent photosynthetic capacities, leaf temperatures are predicted from the foliar radiation budget, *ψ* is predicted from steady-state hydraulics, *G* is formulated after Potkay *et al.*’s (2022) turgor-driven growth model (in which an NSC-unlimited potential turgor is estimated empirically from simulated *ψ*, which determines an NSC-unlimited potential *G*_0_ that sets the maximum for realized *G*), and *G* and *R*_M_ are temperature dependent and NSC limited following Michaelis–Menten kinetics (Thornley 1970, 1971). We modified the GOSM [see Supporting Information—**Notes S1 and Section S1**] by simplifying the mathematics for stem xylem hydraulics and *G*, quickening the computation of ∂*G*/∂*E* with negligible effects on predictions [see Supporting Information—**Fig. S4**]. We added three new processes: (1) temperature-dependent hydraulic conductances, which limit stomatal opening under cold weather (Lintunen *et al.* 2020), (2) NSLs, here modelled by reducing apparent photosynthetic capacities under excessively negative *ψ* (Zhou *et al.* 2013; Novick *et al.* 2016; Dewar *et al.* 2018), since they may be key to explaining stomatal behaviour when conditions are unfavourable for growth (Potkay and Feng 2023), and (3) HRR, which may be partially responsible for explaining nocturnal stomatal opening (Fig. 1B).

### Dynamic simulations

We applied the GOSM and parameters of Potkay and Feng (2023) for Scots pine to perform both year-long and day-long simulations of the *g*_w_, *A*_n_, *E*, *λ*, *η*, *C*, *ψ*_L_ and *G* that maximize growth under cyclic environmental conditions. Parameters for new processes are shown in Table 2. Four original parameters related to sink demand from Potkay and Feng (2023) were adjusted to produce realistic NSC ranges and NPP:GPP ratios in year-long simulations (Table 2; see Supporting Information—**Fig. S5**). The *η* and *C* dynamics (Equations 3 and 4) are simulated by explicit finite difference with 0.5-h time step with cyclic boundary conditions (*η*(*t*_1_) = *η*(*t*_2_), $\underset{t_{1}}{\overset{t_{2}}{\int }}\;\eta \frac{\partial R_{M}}{\partial C}-\left(1-\frac{\eta }{1-f_{c}}\right)\frac{\partial G}{\partial C}\;dt=0,$*C*(*t*_1_) = *C*(*t*_2_), $\underset{t_{1}}{\overset{t_{2}}{\int }}\;a_{L}A_{n}-R_{M}-\frac{G}{1-f_{c}}\;dt=0$; see Supporting Information—**Fig. S6**). Atmospheric conditions are based on NOAA reanalysis data (NCEP/NCAR Reanalysis 1; Kalnay *et al.* 1996) for forest stands in Northern Spain (Tillar Valley; Poblet Forest Natural Reserve; Prades Mountains), where several site-specific hydraulic and growth parameters were estimated (Potkay *et al.* 2021, 2022). Details concerning environmental forcings and numerical solutions are presented in Supporting Information—**Notes S1, Section S2, Figs. S2, S3 and S5**.

**Table 2.** New and changed GOSM parameters.

Symbol	Meaning	New value	Old value	Units	Source
Non-stomatal limitations (NSLs)					
*a*_c_^*^	Shape parameter controlling the decline in apparent *V*_*c*,max_ with decreasing *ψ*_L_	2.92	—	MPa^−1^	Tuzet *et al.* (2003); Nadal-Sala *et al.* (2021) for Scots Pine
*a*_j_^*^	Shape parameter controlling the decline in apparent *J*_max_ with decreasing *ψ*_L_	2.92	—	MPa^−1^	
*ψ*_L,c,50_	*ψ*_L_ at which the apparent *V*_*c,*max_ has been halved	−1.71	—	MPa	
*ψ*_L,j,50_	*ψ*_L_ at which the apparent *J*_max_ has been halved	−1.71	—	MPa	
Hydraulically regulated respiration (HRR)					
*b*_r_^*^	Shape parameter controlling the exponential decline in *R*_d_ with loss of RWC_L_	2	—	—	Chosen as an intermediate value from the range of species’ values (0 to 5) determined by fitting Supporting Information— Equation S1.3.13 to the positively correlated portion of *RWC*_L_ − *R*_d_ data (RWC_L_ > 0.6) from Flexas *et al.* (2005) and Galmés *et al.* (2007)
*π*_L,0_	Leaf osmotic potential at full hydration	−2	—	MPa	Chosen as relatively drought-resistant values from range reported in Bartlett *et al.* (2012) for temperate conifers
*ε*_L_	Leaf modulus of elasticity	14	—	MPa	
Temperature-dependent hydraulic conductance					
*k*_L,25 °C_	Maximum leaf conductance per leaf area at 25 °C	2.02 × 10^−2^	—	mol∙m^−2^∙s^−1^∙MPa^−1^	Modified from Potkay and Feng’s (2023)*k*_L_ parameter to account for difference in *T*_L_ and *T*_a_
*k*_S,25 °C_	Maximum stem conductance at 25 °C	9.76 × 10^-2^	—	mol∙s^−1^∙MPa^−1^	Same values as Potkay and Feng’s (2023)*k*_S_ and *k*_R_ parameters
*k*_R,25 °C_	Maximum belowground conductance at 25 °C	2.30 × 10^−2^	—	mol∙s^−1^∙MPa^−1^	
Q_10,*k*L_	Q_10_ describing increase in *k*_L_ with *T*_L_	1.60	—	—	Matzner and Comstock (2001); Sack *et al.* (2004)
Q_10,*k*S_	Q_10_ describing increase in *k*_S_ with *T*_a_	1.25	—	—	Cochard *et al.* (2000)
Q_10,*k*R_	Q_10_ describing increase in *k*_R_ with *T*_a_	2.20	—	—	Wan *et al.* (2001)
Sink demand					
${\tilde{\phi }}_{{}_{25\;{}^{\circ }C}}\;$	‘Effective’ whole-stem extensibility at 25 °C	1.43 × 10^−8^	4.6 × 10^−8^	MPa^−1^∙s^−1^	Indirectly estimated such that our year-long simulation produced an annual net-to-gross primary productivity ratio of ~0.45 (Waring *et al.* 1998; Collalti *et al.* 2020) as well as an annual *C* range of ~60 to ~160 mol based on *C* estimates by Schiestl-Aalto *et al.* (2019) for Scots Pine of approximately the same height as our simulated tree [see Supporting Information—Fig. S5]
*R*_M,0,25 °C_	Maximum stem and root maintenance respiration rate at 25 °C	8.17 × 10^−6^	5.0 × 10^−5^	mol∙s^−1^	
*γ*_g_	Shape parameter controlling decline in *G* under low NSCs	3.71 × 10^−3^	2.6 × 10^−1^	—	
*γ*_r_	Shape parameter controlling decline in *R*_M_ under low NSCs	7.06 × 10^−3^	3.8 × 10^−1^	—	

*V*_c,max_, maximum carboxylation rate; *J*_max_, maximum electron transport rate; *ψ*_L_; leaf water potential; *R*_d_, leaf dark respiration; RWC_L_, leaf relative water content; *T*_L_, leaf temperature; *T*_a_; air temperature; *k*_L_, leaf conductance per leaf area; *k*_S_, stem conductance; *k*_R_, belowground conductance; *G*, growth rate; *R*_M_, stem and root maintenance respiration rate; *C*, whole-tee non-structural carbohydrate (NSCs) pool.

We present cyclic year-long simulations over an average year (*t*_1_ = 0, *t*_2_ = 365 days), reflecting environmental conditions that vary within and among days and seasons, but not across years [**see** Supporting Information—**Figs**. **S2 and S3**]. Additionally, we compare year-long simulations to day-long simulations for individual days of the year (DOY; *t*_1_ = 0, *t*_2_ = 24 h) and to Potkay and Feng’s (2023) steady-state solution to the GOSM. Two steady-state simulations in which $\dot{C}=0$ and $\dot{\eta }=0$ were performed. First, stomata were optimized for instantaneous midday environmental conditions, and second, stomata were optimized for diel-averaged constant conditions [see Supporting Information—**Notes S1 and Section S3**]. The steady-state solution to diel-averaged conditions is similar to the cyclic day-long simulation, except *C* and *η* are constant within a day instead of being solved by Equations (2) and (4), and boundary conditions are not defined in steady-state simulations.

### Nocturnal stomatal opening

**Figure 3.**
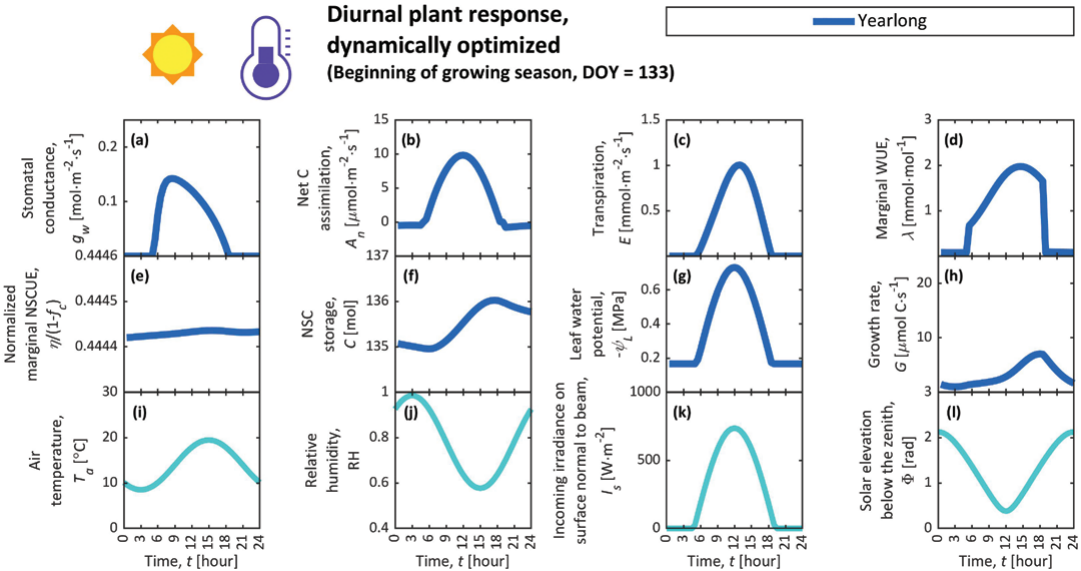
Diel trends from the year-long simulation during the beginning of the growing season (DOY = 133), including the stomatal conductance (*g*_w_) (**A**), photosynthetic carbon assimilation (*A*_n_) (**B**), transpiration (*E*) (**C**), marginal water-use efficiency (WUE; *λ*) (**D**), marginal NSC-use efficiency (NSCUE; *η*) normalized by its maximum value (1 − *f*_*c*_) (**E**), NSC storage (*C*) (**F**), leaf water potential (*ψ*_L_) (**G**) and growth rate (*G*) (**H**) as plant responses. Environmental conditions are shown in the bottom row, including trends in air temperature (*T*_a_) (**I**), relative humidity (RH) (**J**), incoming irradiance on a surface normal to the beam (*I*_s_) (**K**) and zenith angle (solar elevation below the zenith; Φ) (**L**). Day-long simulations for this day (DOY 133) did not converge to a result that satisfied the boundary conditions for *C* and *η* (i.e. Δ*η* = 0 and Δ*C* = 0 isolines do not intersect; see Supporting Information—Fig. S6, e.g. of intersecting isolines).

**Figure 4.**
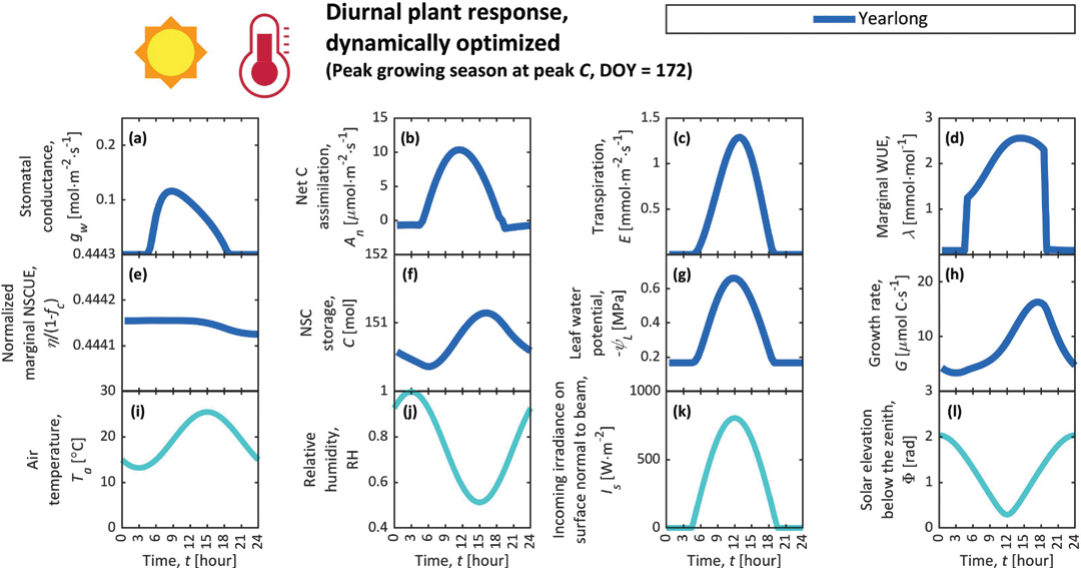
Diel trends from year-long simulation during the peak growing season, particularly during peak annual NSC reserves (*C*) (DOY = 172), including the stomatal conductance (*g*_w_) (**A**), photosynthetic carbon assimilation (*A*_n_) (**B**), transpiration (*E*) (**C**), marginal water-use efficiency (WUE; *λ*) (**D**), marginal NSC-use efficiency (NSCUE; *η*) normalized by its maximum value (1 − *f*_c_) (**E**), NSC storage (*C*) (**F**), leaf water potential (*ψ*_L_) (**G**) and growth rate (*G*) (**H**) as plant responses. Environmental conditions are shown in the bottom row, including trends in air temperature (*T*_a_) (**I**), relative humidity (RH) (**J**), incoming irradiance on a surface normal to the beam (*I*_s_) (**K**) and zenith angle (solar elevation below the zenith; Φ) (**L**). Day-long simulations for this day (DOY 172) did not converge to a result that satisfied the boundary conditions for *C* and *η* (i.e. Δ*η* = 0 and Δ*C* = 0 isolines do not intersect; see Supporting Information—Fig. S6, e.g. of intersecting isolines).

**Figure 5.**
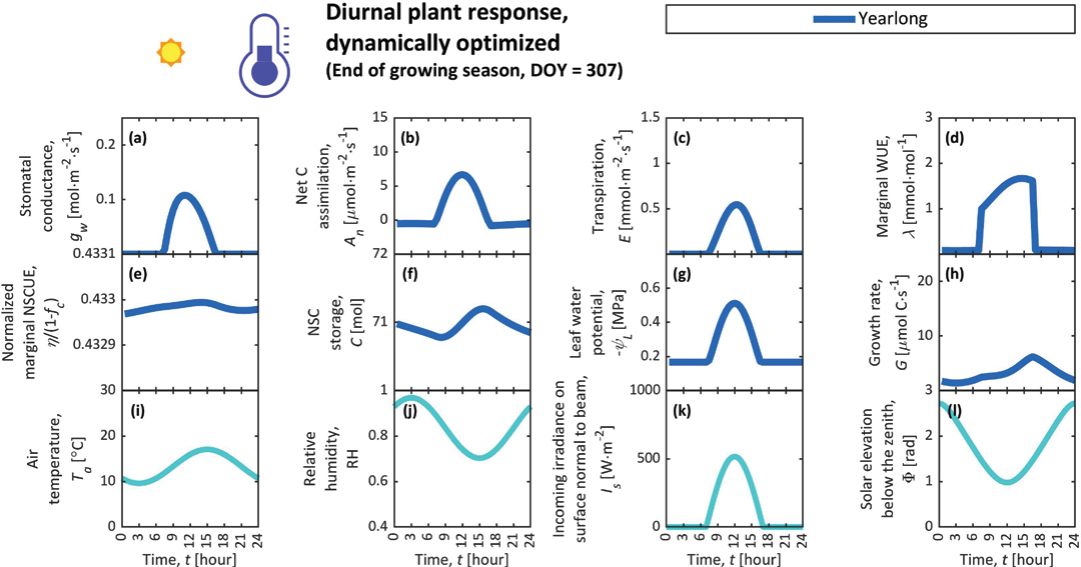
Diel trends from the year-long simulation during the end of the growing season (DOY = 307), including the stomatal conductance (*g*_w_) (**A**), photosynthetic carbon assimilation (*A*_n_) (**B**), transpiration (*E*) (**C**), marginal water-use efficiency (WUE; *λ*) (**D**), marginal NSC-use efficiency (NSCUE; *η*) normalized by its maximum value (1 − *f*_*c*_) (**E**), NSC storage (*C*) (**F**), leaf water potential (*ψ*_L_) (**G**) and growth rate (*G*) (**H**) as plant responses. Environmental conditions are shown in the bottom row, including trends in air temperature (*T*_a_) (**I**), relative humidity (RH) (**J**), incoming irradiance on a surface normal to the beam (*I*_s_) (**K**) and zenith angle (solar elevation below the zenith; Φ) (**L**). Day-long simulations for this day (DOY 307) did not converge to a result that satisfied the boundary conditions for *C* and *η* (i.e. Δ*η* = 0 and Δ*C* = 0 isolines do not intersect; see Supporting Information—Fig. S6, e.g. of intersecting isolines).

To test our hypotheses that diel NSCs and temperature fluctuations explain nocturnal stomatal opening, we numerically solved *C* and $\tilde{\phi }(T_{a})$ combinations that simulate realistic nocturnal stomatal conductance (*g*_w,N_). Values of 0.005 and 0.04 mol∙m^−2^ s^−1^ were chosen as end members considering that *g*_w,N_ is typically 5–40 % of daytime *g*_w_ (Caird *et al.* 2007), assuming daytime *g*_w_ = 0.1 mol∙m^−2^∙s^−1^, based on mid-growing season values from our year-long simulation (Fig. 2A). Simulations were performed at Potkay and Feng’s (2023) default environmental conditions, except RH and *I*_s_, which were set to 0.5 and 0 W∙m^−2^, respectively, and *η* = 0.44∙(1 − *f*_c_) based on our year-long simulation (Fig. 2E). We did not directly test our hypothesis that diel *η* fluctuations explain nocturnal stomatal opening, because simulated *η* is nearly constant (Figs. 2–5) and thus cannot explain nocturnal stomatal opening. Although *η* rises nocturnally for DOY 1–146 and DOY 305–365 (Fig. 2E), thereby lowering *χ*_w_ from day to night, the increase in *η* is far too slight to achieve the small nocturnal *χ*_w_ required for nocturnal stomatal opening. All else being equal, *η* would have to increase ~7 % nocturnally relative to its daytime value (for *f*_f_ = 0.15 and a daytime *η* of 0.44∙[1 − *f*_c_]). However, the simulated *η* change between day and night never exceeds 0.2 % and is negative for approximately half of the year (Fig. 2E).

With a minimal NSC storage of *C* = 60 mol, when *g*_w,N_ is most sensitive to NSC changes, and the corresponding $\tilde{\phi }$ that produced *g*_w,N_ = 0.04 mol∙m^−2^∙s^−1^ at same reference conditions, we simulated the instantaneous response of *g*_w,N_ to leaf-to-air vapour pressure deficit (VPD; *D*_L_; by varying RH), soil water potential (*ψ*_soil_) and atmospheric CO_2_ concentration (*c*_a_), keeping *C* constant. These environmental responses were repeated at elevated (+25 %) and depleted (−25 %) NSC storages to represent delayed responses caused by slower changes in NSC status associated with VPD, drought and eCO_2_ (Muller *et al.* 2011; Dietze *et al.* 2014; Du *et al.* 2020). According to a meta-analysis of NSCs under various experiment treatments by Du *et al.* (2020), the average effects of warming, drought and eCO_2_ on total NSC concentrations are 9 %, −4 % and 24 %, respectively. Hence, our ±25 % was chosen as a maximal value, coinciding with the greatest change in *g*_w,N_.

## Results

### Annual trends

Annual trends in midday *g*_w_, *A*_n_, *E*, *λ*, *η*, *C*, *ψ*_L_ and *G* from our year-long simulation are shown in Fig. 2. During the beginning and end of the growing season, *λ* is small, suggesting that stomata are nearly maximizing *A*_n_ instantaneously (*dA*_n_/*dE* approaching 0) at these times. Counterintuitively, our GOSM predicts that *g*_w_ is greatest during the cold, dormant periods at the beginning and end of the year, resulting from high RH, low *T*_a_ [see Supporting Information—**Fig. S3**] and small VPD (Fig. 2A). Large *g*_w_ under low VPD is unsurprising, considering AOHs and other empirical formulations for *g*_w_ often predict similar results (Oren *et al.* 1999; Katul *et al.* 2009). In fact, low VPD conditions are often ignored in studies for this reason (Ewers and Oren 2000), and predictions are often corrected by simply constraining *g*_w_ below a parameterized maximum *g*_w_ (i.e. *g*_w_ = min(*g*_w_^opt^, *g*_w_^max^), where *g*_w_^opt^ is the optimal solution, and *g*_w_^max^ is the maximum conductance; Mäkelä *et al.* 1996) or by constraining VDP above a threshold (*D*_L_ ≥ 0.05 kPa in CLM5; Franks *et al.* 2018). These large *g*_w_ had little impact on *A*_n_ and *G* (Fig. 2B and H), which control *C* and *η* dynamics (Equations 3 and 4), since *A*_n_ and *G* were both temperature limited (Bernacchi *et al.* 2001; Parent *et al.* 2010), and *A*_n_ was light limited [see Supporting Information—**Fig. S3**].

Midday values of *A*_n_ and *E* begin to rise from their winter values on the ~45th and 66th DOY. Rising *E* at the beginning of the growing season weakly affects the early seasonal trend of *ψ*_L_, which is also modulated by temperature seasonality due to the temperature dependence of hydraulic conductance. In fact, by midseason, *ψ*_L_ becomes less negative over time, despite rising *E*, because of the positive effects of warmth on hydraulic conductance, reducing the potential difference required to drive flow. Soon after *A*_n_ and *E* elevate, *T*_a_ rises enough for growth to begin. Throughout the early growing season, *g*_w_ declines. Meanwhile |*ψ*_L_|, *η*, *A*_n_, *C*, *E*, *λ* and *G* rise until peaking on DOY 119, 146, 163, 172, 195 (approximately at peak *I*_s_), 215 and 220 (at peak *T*_a_), respectively, and then decline. During the growing season, midday *g*_w_ ≈ 0.1 mol∙m^−2^∙s^−1^, comparing well to the upper bound of measurements from our site on healthy Scots pine (Poyatos *et al.* 2013; Salmon *et al.* 2015). The diel changes in *η* (i.e. *dη*/*dt*) inversely mirror *C* with an ~80-day lag (i.e. high and low *C* later cause *η* to decline and rise between days, respectively), displaying the *carbon-use strategy*. Nonetheless, *η* is approximately constant, suggesting that *g*_w_ is controlled more by NSC dynamics (*C*) than by variations in sink demand (*η*).

Day-long simulations satisfied both of our cyclic boundary conditions for *η* and *C* (*η*(*t*_1_) = *η*(*t*_2_), $\underset{t_{1}}{\overset{t_{2}}{\int }}\;\eta \frac{\partial R_{M}}{\partial C}-\left(1-\frac{\eta }{1-f_{c}}\right)\frac{\partial G}{\partial C}\;dt=0$, and *C*(*t*_1_) = *C*(*t*_2_), $\underset{t_{1}}{\overset{t_{2}}{\int }}\;a_{L}A_{n}-R_{M}-\frac{G}{1-f_{c}}\;dt=0$; see Supporting Information—**Fig. S6**) only on DOY 174–210 and 309–334 when midday *C* was nearly constant across days in the year-long simulation (i.e. at the seasonal maximum and minimum *C*; see Supporting Information—**Fig. S7**). Day-long, year-long and both steady-state simulations of *A*_n_ compared well on these days, and day-long simulations and steady-state simulations to diel-averaged conditions agreed for all variables. In addition to *A*_n_, day-long and both steady-state simulations agreed only in terms of *η*. Meanwhile, steady-state simulations to instantaneous midday conditions agreed with year-long simulations for *E* and *G*. However, for *g*_w_, *λ*, *C* and *ψ*_L_, year-long predictions and steady-state predictions to instantaneous conditions differed from each other as well as from day-long simulations and diel-averaged steady-state simulations. Notably, day-long and steady-state simulations over-predicted *C* more often than not. The steady-state solution to instantaneous midday conditions predicted infinite *C* over the growing season, because midday whole-canopy photosynthate input (*a*_L_ × *A*_n_) exceeds the maximum possible midday sink demand (*G*_0_/(1 − *f*_c_) and *R*_*m*,0_ in Potkay and Feng 2023), meaning that there is no optimal stomatal behaviour that prevents NSCs from accumulating between days at these times according to Potkay and Feng’s (2023) instantaneous steady-state strategy; instead, a dynamic perspective is necessary. For day-long simulations and diel-averaged steady-state solutions, these *C* disparities declined while approaching DOY 172 and 325 when year-long-simulated midday *C* was nearly constant across days as source- and sink demand were near equal.

### Diel trends

We present diel trends from our year-long simulation for individual days throughout the growing season, including an example of the early growing season (DOY 133; Fig. 3), three examples during the peak growing season, specifically when *A*_n_, *C* and *G* peak (DOY 163, 172 and 220, respectively; Fig. 4; see Supporting Information—**S8 and S9**), and an example of the late growing season (DOY 307; Fig. 5). In day-long simulations, cyclic boundary conditions for *η* and *C* were not satisfied for these key dates. Noon values from individual day-long simulations that met the cyclic boundary conditions (DOY 174–210 and 309–334) are shown in Supporting Information—**Fig. S7**. Stomata generally open most in the morning and then gradually close through the day (Figs 3–5; **see** Supporting Information—Figs**. S8 and S9**). Maximal *g*_w_ at dawn predominantly results from low morning VPD (low *T*_a_, high RH). During the early growing season, these large morning *g*_w_ also result from small *χ*_w_ (predominantly due to cold temperatures and low NSCs; Figs. 2 and 3). In year-long solutions, *λ* tends to rise throughout each day, peaking at approximately the same time of day that *C* peaks (Figs. 3–5; **see** Supporting Information—Figs**. S8** and **S9**), emphasizing the role of *C* on stomatal behaviour over diel timescales. In other words, *λ* rises with *C* throughout the day, prompting higher water-use efficiency and stomatal closure. Additionally, *η* and *C* appear nearly constant throughout the day (Figs. 3–5; **see** Supporting Information—Figs**. S8 and S9**), while *dη*/*dt* and *C* display opposing sinusoidal patterns, suggesting negative feedback between *C* and *η* like those seen in seasonal trends (Fig. 2), although far smaller in magnitude, and displaying a reduced form of the *carbon-use strategy* at diel timescales. Though growth occurs during both day and night, maximum diel *G* is simulated in the late afternoon when air is warmest and *C* is slightly elevated (Figs 3–5; **see** Supporting Information—Figs**. S8 and S9**).

### Nocturnal stomatal opening

Our GOSM requires either very cold temperatures (*T*_a_ ≈ 5 °C) or unrealistically small NSCs to simulate realistic *g*_w,N_ [**see** Supporting Information—**Fig. S10**], meaning that nocturnal stomatal opening would be simulated only during the winter and early spring. Since growth is both temperature- and NSC limited, these required small *T*_a_ and *C* would negate virtually all growth at night. Simulated diel *C* and *η* changes are too small to significantly alter *χ*_w_ between day and night (Figs. 3–5; see Supporting Information—Figs**. S8 and S9**). Though *χ*_w_ varies slightly between day and night, these variations arise predominantly from diel *T*_a_ changes. These results do not support our hypotheses that diel *T*_a_, *C* and *η* variations explain nocturnal stomatal opening.

**Figure 6.**
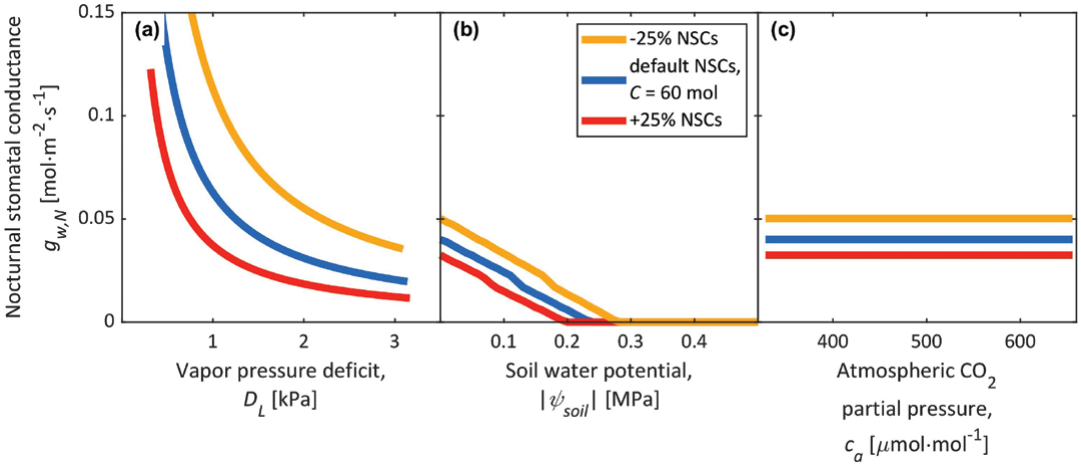
Instantaneous response of nocturnal stomatal conductance (*g*_w,N_) to vapour pressure deficit (*D*_L_; **A**), soil water potential (*ψ*_soil_; **B**) and atmospheric CO_2_ partial pressure (*c*_a_; **C**) at three NSC storages (75 %, 100 %, and 125 % of a reference storage size of *C* = 60 mol). Simulations were performed at atmospheric CO_2_ and O_2_ partial pressures of 410 μmol∙mol^−1^ (in **A** and **B**) and 207 mmol∙mol^−1^, respectively, RH = 0.5 (in B and C), a soil water potential of 0 MPa (in **A** and **C**), a solar elevation below the zenith of 0 radians, and an incoming irradiance of 0 W∙m^−2^, using parameters from Potkay and Feng (2023) and Table 1. Air temperature (*T*_a_) was set to 25 °C for most calculations (e.g. leaf temperature, *R*_d_, *λ*), except for exclusively the calculation of the extensibility ($\tilde{\phi }$), for which *T*_a_ = 8.07 °C.

Upon changing $\tilde{\phi }$ to a relatively small value that produced *g*_w,N_ = 0.04 mol∙m^−2^∙s^−1^ at our reference conditions and *C* = 60 mol ($\tilde{\phi }$ equal to ~5 % of the value in Table 2), we simulated the environmental responses of *g*_w,N_ at constant *C* (Fig. 6). By reducing $\tilde{\phi }$ to lessen *χ*_w,N_, we effectively apply Wang *et al.*’s (2021) approach with a constant ‘fitness factor’. At a given *C*, hydraulic stress (VPD, soil drought) closes stomata, while CO_2_ concentration has no immediate effect on *g*_w,N_ (Fig. 6). Depleting *C* opened stomata (Fig. 6), while *C* accumulation closed stomata nocturnally (Fig. 6). Conversely, long droughts deplete NSCs and open stomata at night (Fig. 6).

## Discussion

### Dynamic optimization and plants’ *timescales of optimization*

Plants are theorized to optimally coordinate their supply and demand for resources over relevant timescales. Natural selection acts over this *timescale of optimization*, since selection has generated plants’ adaptive regulatory patterns, which remains a major uncertainty (Dewar *et al.* 2009). Ideally, the *timescale* should be the period over which the objective is integrated (*t*_2_ − *t*_1_ in Equation 1). Individual plant traits may operate over different *timescales*, and they certainly differ in the bounds of integration (*t*_2_ − *t*_1_) that modellers use to predict them through mathematical optimization. It is common to choose longer bounds for traits that change slower (e.g. assuming that quickly changing *g*_w_ is instantaneously optimized; Wolf *et al.* 2016). However, rapid kinetics do not necessarily imply shorter mathematical bounds (Feng *et al.* 2022). For realistic predictions, the choice of bound length does not necessarily have to equal or even approximate the true *timescale* over which natural selection acts. The mathematical bounds of integration must only describe the shortest portion of the *timescale* in which the temporal dynamics of the chosen constraints (soil−water in water-saving AOHs, NSCs in the GOH) repeat (e.g. the *timescale* is treated as the sum of repeated periods that are identical or effectively the same in a stochastic sense). Though the *timescale* for stomata has been theorized as plants’ lifetime (Cowan 1986) and may be even longer, water-saving AOHs often approximate the *timescale* as many repeated soil–water dry-downs between rain events (Cowan 1982; Mäkelä *et al.* 1996; Manzoni *et al.* 2013; Mrad *et al.* 2019). Conversely, we have approximated the *timescale* as multiple repeated years, the shortest period over which NSC trends are cyclic (Oswald and Aubrey 2023).

Since stomatal conductance, NSCs, and growth rates follow near-cyclic trends over diel and seasonal timescales (Smith and Stitt 2007; Maseyk *et al.* 2008; Rossi *et al.* 2013; Martínez-Vilalta *et al.* 2016; Mencuccini *et al.* 2017; Tixier *et al.* 2018, 2020), we simulated plant responses that maximize growth over individual days (*t*_2_ − *t*_1_ = 24 h) and a full year (*t*_2_ − *t*_1_ = 365 days) and compared these dynamic simulations to Potkay and Feng’s (2023) steady-state solution (Figs. 2–5; see Supporting Information—Figs**. S8 and S9**). Year-long simulations captured realistic seasonal and diel patterns, supporting the GOH. Cyclic day-long simulations, however, were possible only for the middle and end of the growing season when changes in *C* between days were small in year-long simulations and when maximum possible sink demands exceeded instantaneous carbon input (when steady-state simulations would predict finite *C*). On these days, year-long, day-long and diel-averaged steady-state simulations generally compared well in terms of *A*_n_, though not in terms of other predicted variables [**see** Supporting Information—**Fig. S7**], especially not in their *C* predictions. This suggests that cyclic boundary conditions (*C*(*t*_1_) = *C*(*t*_2_)) and steady state are inappropriate to describe NSC use for an individual DOY, since real NSCs change over multiple days and are thus non-cyclic across days. The amplitude of simulated NSC cycles depends on the duration of the simulation and shrinks as *t*_2_ − *t*_1_ becomes smaller, approaching the steady-state solution as *t*_2_ − *t*_1_ approaches zero. In day-long simulations, *t*_2_ − *t*_1_ is small, and thus day-long and diel-averaged steady-state simulations approximate each other [**see** Supporting Information—**Fig. S7**]. Hence, day-long simulations over-predict *C* for the same reason that steady-state simulations do: they ignore the fact that instantaneous *A*_n_ during the peak growing season exceeds the average *A*_n_ over a year, thereby overestimating *C*. Sink demands during the peak growing season similarly exceed their annual averages, but to a much lesser extent, since plants respire throughout the entire year, while peak photosynthesis occurs over a smaller window (at least in seasonal environments). More appropriate boundary conditions for *C* in dynamic day-long simulations would set *C*(*t*_1_) to a realistic predetermined value. *C*(*t*_2_) would be treated implicitly (explicit definition is non-essential). However, the appropriate value for *C*(*t*_1_) is unclear. It could be chosen from measurements of whole-tree NSCs, although these estimates are rare, or it could be taken as the value solved by the full year-long simulation at the specified DOY. The latter choice, however, makes the day-long simulation unnecessary since the year-long simulation makes all of the same predictions over a longer period. The dynamic version of the GOSM presented here is an approvement upon Potkay and Feng’s (2023) steady-state GOSM, which cannot capture how *C* and *η* evolve dynamically over hours to seasons. Hence, the dynamic GOSM better describes how stomata respond to environmental variation as modulated by the changes in NSC storage (*C*) and the cost of sink demand (*η*).

Both year-long and day-long simulations predicted that maximum diel *G* typically occurs in the late afternoon when air is warmest and *C* is slightly elevated (Figs. 3–5; **see** Supporting Information—Figs**. S8 and S9**), clashing with expectations and observations that most volumetric growth and turgor-driven cell expansion occur at night (Steppe *et al.* 2015; Zweifel *et al.* 2021; but see Mencuccini *et al.* 2017). Accordingly, most volumetric growth is expected at night when *ψ* and turgor are greatest. This expectation, however, ignores temperature limitations on expansion (Cabon *et al.* 2020; Peters *et al.* 2021). In other simulations in which *T*_a_ is constant throughout day and night (not shown), most growth indeed occurs at night when *ψ* and turgor are largest. Volumetric growth (e.g. cell expansion, division) is only one aspect of growth (Hilty *et al.* 2021), while our GOSM describes growth gravimetrically through an implicit assumption that expansion and cell wall hardening are synchronized (i.e. wood density is assumed constant in Potkay *et al.* 2022). Hence, it is not entirely appropriate to compare our simulations of gravimetric growth to observations of volumetric growth, which are known to follow distinct temporal patterns (Rathgeber *et al.* 2016). For example, cell wall hardening is expected to be NSC dependent (Hölttä *et al.* 2010; Cartenì *et al.* 2018; Friend *et al.* 2023) and temperature dependent (Cuny *et al.* 2015; Cuny and Rathgeber 2016), suggesting that, unlike expansion, hardening may be faster during the day, which is consistent with our simulations. Nonetheless, our representation of growth should be improved. Future versions of the GOH should model the phases of wood formation separately to distinguish between volumetric growth, gravimetric growth, and their distinct dynamics and sensitivities to environmental conditions by integrating ideas from xylogenesis models (Eckes-Shephard *et al.* 2022). The GOH’s objective function (Equation 1) would be rewritten as maximizing volumetric instead of gravimetric growth, and we expect changes to our predictions of *g*_w_ and *C*. Water-use efficiency (*χ*_w_) and *C* would both be higher (resulting in lower *g*_w_) than currently predicted (Fig. 2) in the early growing season when cambial and developing xylem cells are dividing and expanding (larger ∂*G*/∂*E* term in Equation (3) because of shorter, more-concentrated period of expansion than our current growth scheme) with little NSC deposition, since cell wall hardening would have not begun. Conversely, *χ*_w_ and *C* would both be lower (with higher *g*_w_) in the late growing season as increasingly more developing xylem cells end their expansion (∂*G*/∂*E* approaching zero) and transition into cell wall hardening, when most NSC deposition occurs.

### Carbon-use strategies

Much like how some AOHs suggest that stomata follow *water-use strategies* that moderate current transpiration to save soil–water for future uptake (Manzoni *et al.* 2013; Mrad *et al.* 2019), the GOH suggests that stomata also follow *carbon-use strategies* to maintain NSC homeostasis (Smith and Stitt 2007; Schönbeck *et al.* 2018; Dickman *et al.* 2019). When NSCs are high, *η* declines (Equation 4; Fig. 2), both of which close stomata (Fig. 1A), increasing *ψ*, turgor and *G*, while reducing *A*_n_ and NSCs through decreased carbon supply and increased demand. When NSCs are low, *η* rises, and stomata open to improve *A*_n_ and reduce turgor and *G*, accumulating NSCs through increased supply and decreased demand. This *carbon-use strategy* was evident in year-long optimizations (Fig. 2) and day-long optimizations, though diel *C* − *η* feedbacks were far smaller than seasonal feedbacks. These regulatory, negative feedbacks are driven by *C* changes predominately and *η* changes to a lesser extent, since seasonal and diel *η* changes were small (Figs. 2–5; **see** Supporting Information—Figs**. S8 and S9**). These results suggest that carbon, in addition to water, is a resource that limits stomatal opening (Buckley 2023).

The *carbon-use strategy* can explain why maximal photosynthetic stimulation by eCO_2_ and coinciding heightened stomatal sensitivity typically occur at the beginning of the growing season (low *χ*_w_ during the early growing season in Fig. 2; Quentin *et al.* 2015; Urban *et al.* 2019; Sanches *et al.* 2020; Lauriks *et al.* 2021*b*, 2022) when NSCs deplete to meet growth demands (Palacio *et al.* 2018; Tixier *et al.* 2020). Since larger seasonal NSC fluctuations occur in more seasonal climates (Fermaniuk *et al.* 2021), we expect seasonal variations in photosynthetic and stomatal sensitivity to eCO_2_ to be pronounced in boreal ecosystems. Similarly, the *carbon-use strategy* can explain stomatal behaviour’s apparent coordination with NSC depletion during droughts. According to the GOH, drought-induced NSC depletion has a negative effect on *χ*_w_ that counters the rise of *χ*_w_ due to hydraulic stress, slowing stomata closure, consistent with observations of aggressive water use and accelerated desiccation in NSC-depleted plants (O’Brien *et al.* 2014; Sapes *et al.* 2019) and defoliated trees with smaller stem NSC reserves (Poyatos *et al.* 2013; Salmon *et al.* 2015). Unlike the GOH, these two phenomena (seasonal CO_2_ sensitivity, stomata-NSC-drought feedbacks) cannot be explained by most AOHs (e.g. Cowan and Farquhar 1977; Mäkelä *et al.* 1996; Manzoni *et al.* 2013; Wolf *et al.* 2016; Sperry *et al.* 2017), which do not account for NSCs and their role in stomatal behaviour and plant water use (Blonder *et al.* 2023). Only a few AOHs capture NSC-dependent stomatal behaviours, including AOHs that consider the downregulation of photosynthesis under elevated leaf sugar concentrations (predicted by phloem transport models; Nikinmaa *et al.* 2013; Hölttä *et al.* 2017; Dewar *et al*. 2022) and an AOH, which explicitly defines a cost of maintaining leaf osmotic potentials (Deans *et al.* 2020), which we interpret as implicitly incorporating a cost of the sugars that generate osmotic potential. In these AOHs, leaf sugar concentrations impose a *cost* to *g*_w_, while all NSCs (sugars, starches) impose a *constraint* to *g*_w_ in our GOSM. Additionally, NSCs in our GOSM are not leaf specific; all NSCs are relevant regardless of organ. In addition to the direct control of NSCs on *g*_w_, NSC demand (represented by *η*) modifies *g*_w_ in the GOSM, which no AOH directly considers (although phloem transport-based AOHs indirectly consider sinks, which impact sugar concentrations).

Our GOH emphasizes NSCs as a key limiting resource by explicitly incorporating the NSC balance as a constraint on growth. This NSC constraint does not mean growth may not also be water limited (Lempereur *et al.* 2015; Eckes-Shephard *et al.* 2021); water conservation over time merely does not currently factor into the current mathematical set-up of our optimization problem (the GOSM nonetheless represents the effects of water limitation on growth and hydraulics). Hence, our current model may offer an incomplete view of stomal behaviour, and the relative contributions of carbon and water limitations to optimal stomatal behaviour remain unknown. Future versions of the GOH should consider both carbon and water as constraints to describe both the *carbon-use strategy* and the *water-use strategy* and identify their relative contributions to stomatal opening and closure. Because we have not constrained the objective (Equation 1) by soil–water, we limited our simulations to scenarios of constant and high soil–water availability. Nonetheless, even without the *water-use strategy*, the GOH would predict lower *g*_w_ and *G* and higher *C* if transpiration depleted soil–water, since the GOH captures these responses under soil–water stress in steady state (Potkay and Feng 2023), and dynamic simulations tend to follow steady-state trends as an attractor. Contrarily, in the absence of hydraulic limitations, water-saving AOHs would predict unrealistically large *g*_w_ in our simulations with constant and high soil–water content, since plant-controlled soil–water depletion is prerequisite to their theory. These large *g*_w_ can be shown by Manzoni *et al.*’s (2022) heuristic model for *g*_w_ for a plant transpiring at a constant rate during a soil–water dry-down, in which *g*_w_ is inversely proportional to the dry-down length (see their Equation 11). In our modelling scenarios with constant and plentiful soil–water, the dry-down length is effectively zero, leading to infinite *g*_w_ according to Manzoni *et al.* (2022). Other water-saving AOHs would predict finite but still similarly large *g*_w_ under constant and high soil–water content. The inclusion of hydraulic limitations also constrains *g*_w_ from being excessively large in water-saving AOHs (Mrad *et al.* 2019; Lu *et al.* 2020); nonetheless, soil–water dry-downs, which are absent from our simulations, are fundamental to these modified water-saving AOHs.

### Nocturnal stomatal opening

We hypothesized that the GOH explains nocturnal stomatal movements, particularly through diel fluctuations in *T*_a_, *η*, and/or *C* (*carbon-use strategy*), since these variables control *χ*_w_ (Fig. 1). Nocturnal stomatal patterns have been thought to maximize growth (Resco de Dios *et al.* 2016) and involve NSCs (Lasceve *et al.* 1997; Easlon and Richards 2009). Support for our hypotheses was mixed. On one hand, either very low *T*_a_ or unrealistically small *C* were prerequisite to realistic *g*_w,N_ [see Supporting Information—**Fig. S10**], nullifying growth at night when most expansion typically occurs (Steppe *et al.* 2015; Zweifel *et al.* 2021), and simulated diel changes in *C* and *η* were too small for the diel variations in *χ*_w_ required to open stomata nocturnally (Figs. 3–5; see Supporting Information—Figs**. S8 and S9**). In fact, diurnal NSC changes are known to be small in trees (e.g. Tixier *et al.* 2018) and might be more relevant in smaller plants that deplete NSCs faster (Smith and Stitt 2007; Oswald and Aubrey 2023). On the other hand, after lowering $\tilde{\phi }$, our GOSM captured several nocturnal stomatal behaviours, including how diminished daytime assimilation (e.g. shading) increases *g*_w,N_ (Barbour *et al.* 2005; Easlon and Richards 2009) by storing fewer NSCs as well as decreased *g*_w,N_ under eCO_2_ (Wheeler *et al.* 1999; Zeppel *et al.* 2012; Resco de Dios *et al.* 2016) upon acclimation by storing more NSCs, and under high VPD (Barbour and Buckley 2007; Howard and Donovan 2010) and soil drought (Zeppel *et al.* 2012, 2014; Chowdhury *et al.* 2021; Wang *et al.* 2021) (Fig. 6). Both eCO_2_ and short periods of hydraulic stress are known to cause NSCs to accumulate (Dietze *et al.* 2014; Mitchell *et al.* 2014; Du *et al.* 2020; Grossiord *et al.* 2020), which cause night-time stomatal closure according to the GOH (Fig. 6). Conversely, long periods of hydraulic stress may deplete NSCs, opening stomata nocturnally (Fig. 6). The GOH suggests that *g*_w,N_ has only a delayed response to CO_2_ concentrations as a long-term *carbon-use strategy*, while *g*_w,N_ responds to hydraulic stress in both instantaneously through immediate growth declines (Lempereur *et al.* 2015; Tumajer *et al.* 2022) and in a delayed manner due to the slower acclimation of NSCs and the *carbon-use strategy*. This instantaneous response on *g*_w,N_ is always negative, while the delayed response may be either positive or negative depending on whether NSCs are depleted or accumulated, respectively, and thus depending on the duration and nature of hydraulic stress. The GOH may also explain positive and neutral responses of *g*_w,N_ to VPD (Barbour *et al.* 2005; Daley and Phillips 2006; Dawson *et al.* 2007; Howard and Donovan 2010; Zeppel *et al.* 2012; Resco de Dios *et al.* 2013*b*; Wang *et al.* 2021) if elevated VPD causes such NSC accumulation. However, we cannot explain reports of nocturnal opening under eCO_2_ (Wheeler *et al.* 1999; Zeppel *et al.* 2012; Resco de Dios *et al.* 2016). We are unaware of any studies reporting a NSC decline under eCO_2_, and hence the literature does not support NSC acclimation as a reason for nocturnal stomatal opening under eCO_2_. Similarly, eCO_2_-induced changes in leaf area cannot explain nocturnal opening, since eCO_2_ tends to increase leaf areas (Lauriks *et al.* 2021*a*). Instead of opening, however, such an increase in leaf area would close stomata further by increasing *χ*_w_ (Manzoni *et al.* 2022; Potkay and Feng 2023). Nonetheless, our GOH offers a step forward to identify the evolutionary advantage of nocturnal stomatal opening, which AOHs are generally unable (Zeppel *et al.* 2014; Yu *et al.* 2019) unless employing an empirical ‘fitness factor’ to predict *χ*_w,N_ from *χ*_w,D_ (Wang *et al.* 2021).

Capturing nocturnal stomatal opening with realistic night-time expansion may require framing the problem in a probabilistic manner that accounts for plants’ statistical ‘memory’ of environmental conditions (Buckley *et al.* 2017; see Supporting Information—**Notes S1**, **Sections S4.1 and** S**4.2**). Indeed, nocturnal stomatal behaviour is regulated by circadian rhythm (Hennessey *et al.* 1993; Resco de Dios *et al.* 2013*a*, 2015, 2020), which probabilistically ‘anticipates’ a range of environmental conditions. Following this idea, we derived a simple expression for Wang *et al.*’s (2021) ‘fitness factor’ accounting for plants’ thermal ‘memory’ [see Supporting Information—E**quation S4.2.6**]:


$$f_{\text{f}}\approx \frac{\frac{〈\overset{\hat{∼}}{\phi }{〉}_{\text{N}}}{\eta }-\frac{\tilde{\phi }(T_{\text{a,N}})}{1-f}}{\frac{〈\overset{\hat{∼}}{\phi }{〉}_{\text{D}}}{\eta }-\frac{\tilde{\phi }(T_{\text{a,D}})}{1-f_{\text{c}}}},$$ (5)

where ${〈\overset{\hat{∼}}{\phi }〉}_{N}$ and ${〈\overset{\hat{∼}}{\phi }〉}_{D}$ are the mean $\tilde{\phi }$ for the ‘anticipated’ night-time and daytime temperature distributions, respectively, and $\phi (T_{\text{a,N}}\text{)}$ and $\phi (T_{\text{a,D}})$ are the $\tilde{\phi }$ evaluated at the actual night-time and daytime temperatures, respectively. While our deterministic GOSM (Equation 3) implicitly assumes plants’ ‘*anticipation*’ of temperature is perfectly *accurate* and *precise*, Equation (5) does not. By *accurate*, we mean the ‘*anticipated*’ temperature distribution is centred on the actual temperature, and by *precise*, we mean the variation of the ‘*anticipated*’ temperature distribution is small. If ‘*anticipation*’ is *accurate* and relatively *precise*, Equation (5) predicts realistic *f*_f_ only on cool days and nights [see Supporting Information—**Fig. S11**], confirming our previous results [see Supporting Information—**Fig. S10**]. In fact, as long as ‘anticipation’ is *accurate*, Equation (5) cannot predict realistic *f*_f_ (~0.05 < *f*_f_ < ~0.25 at 25 °C) on warm nights regardless of the *precision* [see Supporting Information—**Fig. S12**]. To capture realistic *f*_f_, ‘anticipation’ must be *inaccurate*, *‘*anticipating’ night-time temperatures cooler than actuality [see Supporting Information—**Fig. S13**], enabling simultaneous nocturnal stomatal opening and expansion. This thermal *inaccuracy* could arise from a selective ‘*memory*’ and from the fact that leaf temperature (*T*_L_) is not the same as temperatures in other growing tissues (approximated here by *T*_a_). If stomata ‘*anticipated*’ that *T*_a_ was always some function of actual *T*_L_ based on a selective ‘*memory*’ of only daytime experiences (when on average, *T*_a_ < *T*_L_ for *T*_a_ < ~28 °C; Michaletz *et al.* 2016), stomata would ‘anticipate’ temperatures cooler than actuality at night (when *T*_a_ ≈ *T*_L_ in actuality). Alternatively, the thermal *inaccuracy* might reflect a cautious strategy to safeguard against incurring penalties of cold nights that are unaccounted in the GOH framework.

## Supplementary Material

plad044_suppl_Supplementary_Material

## Data Availability

MATLAB codes for model and plotting/analyzing data, including forcing data and simulation outputs are available in the Supporting Information (Notes S2).

## Notes

**Form & Function. Chief Editor:** Kate McCulloh

## Supporting Information

The following additional information is available in the online version of this article –

**Figure S1.** Simulated net assimilation, leaf temperature, and leaf water content corresponding to marginal carbon profits and costs of water shown in Fig. 1.

**Figure S2.** Probability distributions parameters for the interannual variation in air temperature at Northern Spain site.

**Figure S3.** Annual trends in diel minimum and maximum conditions at Northern Spain site.

**Figure S4.** Comparison of new stem hydraulics scheme to Potkay and Feng’s (2023) original scheme.

**Figure S5.** Maximum and minimum NSC isolines and NPP:GPP ratio isoline for given maintenance respiration and extensibility parameter combinations.

**Figure S6.** Example of numerical search for cyclic solution.

**Figure S7.** Annual trends from year-long and day-long simulations.

**Figure S8.** Diel trends from year-long simulations during seasonal peak in net assimilation.

**Figure S9.** Diel trends from year-long simulations during seasonal peak in woody growth.

**Figure S10.** Combinations of NSC storages and air temperatures that result in realistic nocturnal stomatal conductance.

**Figure S11.** Fitness factor for combinations of ‘*anticipated*’ mean daytime and night-time temperatures, assuming that plants’ ‘*anticipation*’ of air temperature is perfectly *accurate.*

**Figure S12.** Fitness factor for combinations of standard deviations and skewness of ‘*anticipated*’ daytime and nighttime temperature distributions, assuming that plants’ ‘*anticipation*’ of air temperature is perfectly *accurate.*

**Figure S13.** Fitness factor for varying *accuracy* or ability to correctly ‘*anticipate*’ air temperatures.

**Notes S1.** Description of model changes, environmental forcing and numerical solution, and probabilistic growth maximization.

**Notes S2.** MATLAB code for model and plotting/analyzing data, including forcing data and simulation outputs.
