# Supplementary material for: Dynamically optimizing stomatal conductance for maximum turgor-driven growth over diel and seasonal cycles
Source: AoB Plants. 2023 Jul 6;15(5):plad044. doi: 10.1093/aobpla/plad044 (PMC10601388; doi:10.1093/aobpla/plad044)
Supplement: plad044_suppl_Supplementary_Material [file plad044_suppl_supplementary_material.pdf]

***AoB PLANTS* Supporting Information**

Article title: Dynamically optimizing stomatal conductance for maximum turgor-driven growth over diel and seasonal cycles

The following Supporting Information is available for this article:

Fig. S1 Simulated net assimilation, leaf temperature, and leaf water content corresponding to marginal carbon profits and costs of water shown in Fig. 1

Fig. S2 Probability distributions parameters for the interannual variation in air temperature at Northern Spain site

Fig. S3 Annual trends in diel minimum and maximum conditions at Northern Spain site

Fig. S4 Comparison of new stem hydraulics scheme to Potkay & Feng's (2023) original scheme

Fig. S5 Maximum and minimum NSC isolines and NPP:GPP ratio isoline for given maintenance respiration and extensibility parameter combinations

Fig. S6 Example of numerical search for cyclic solution

Fig. S7 Annual trends from yearlong and daylong simulations

Fig. S8 Diel trends from yearlong simulations during seasonal peak in net assimilation

Fig. S9 Diel trends from yearlong simulations during seasonal peak in woody growth

Fig. S10 Combinations of NSC storages and air temperatures that result in realistic nocturnal stomatal conductance

Fig. S11 Fitness factor for combinations of '*anticipated*' mean daytime and nighttime temperatures, assuming that plants' '*anticipation*' of air temperature is perfectly *accurate*

Fig. S12 Fitness factor for combinations of standard deviations and skewness of '*anticipated*' daytime and nighttime temperature distributions, assuming that plants' '*anticipation*' of air temperature is perfectly *accurate*

Fig. S13 Fitness factor for varying *accuracy* or ability to correctly '*anticipate*' air temperatures

Notes S1 Description of model changes, environmental forcing and numerical solution, and probabilistic growth maximization

Notes S2 MATLAB code for model and plotting/analyzing data, including forcing data and simulation outputs.

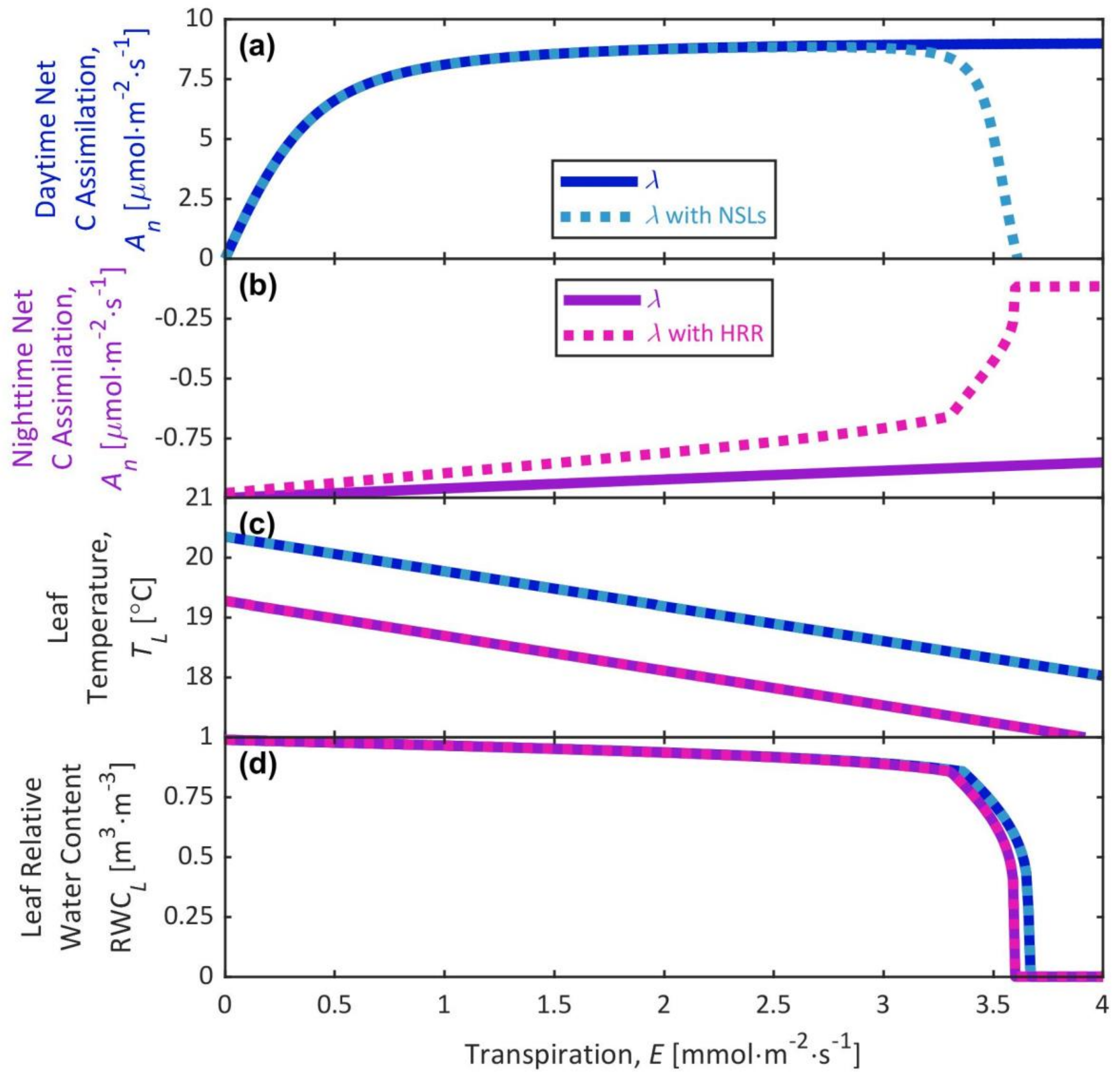

Figure S1: Daytime (a) and nighttime (b) net C assimilation, leaf temperature (c), and leaf relative water content (d) corresponding to estimates of $\lambda = \partial A_n/\partial E$ in Fig. 1 in main text. Here, we plot against transpiration (E) instead of stomatal conductance (g_w; as in Figure 1) to show the slope of E-A_n curves, which equal the marginal carbon profit of water (λ).

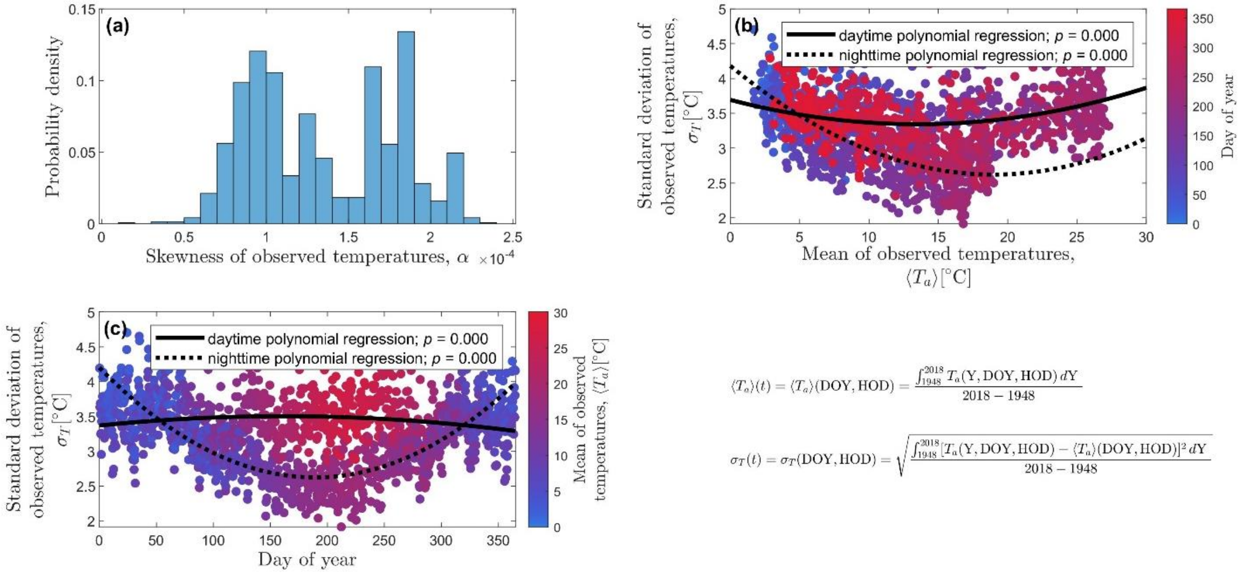

$$\langle T_a\rangle(t) = \langle T_a\rangle(\mathrm{DOY},\mathrm{HOD}) = \frac{\int_{1948}^{2018} T_a(\mathrm{Y},\mathrm{DOY},\mathrm{HOD})\,d\mathrm{Y}}{2018-1948}$$

$$\sigma_T(t) = \sigma_T(\mathrm{DOY},\mathrm{HOD}) = \sqrt{\frac{\int_{1948}^{2018} [T_a(\mathrm{Y},\mathrm{DOY},\mathrm{HOD}) - \langle T_a\rangle(\mathrm{DOY},\mathrm{HOD})]^2\,d\mathrm{Y}}{2018-1948}}$$

Figure S2: Parameters of the probability distributions for the interannual variation in air temperature (T_a) at a given day of year (DOY) and hour of day (HOD) among years (1948-2018) for Northern Spain (Tillar Valley; Poblet Forest Natural Reserve; Prades Mountains), including skewness (α; a), mean ($\langle T_a \rangle$; b&c), and standard deviation (σ_T; b&c). In b&c, polynomial regressions were fit to daytime and nighttime values, where daytime and nighttime were determined from the zenith angle (solar elevation below the zenith).

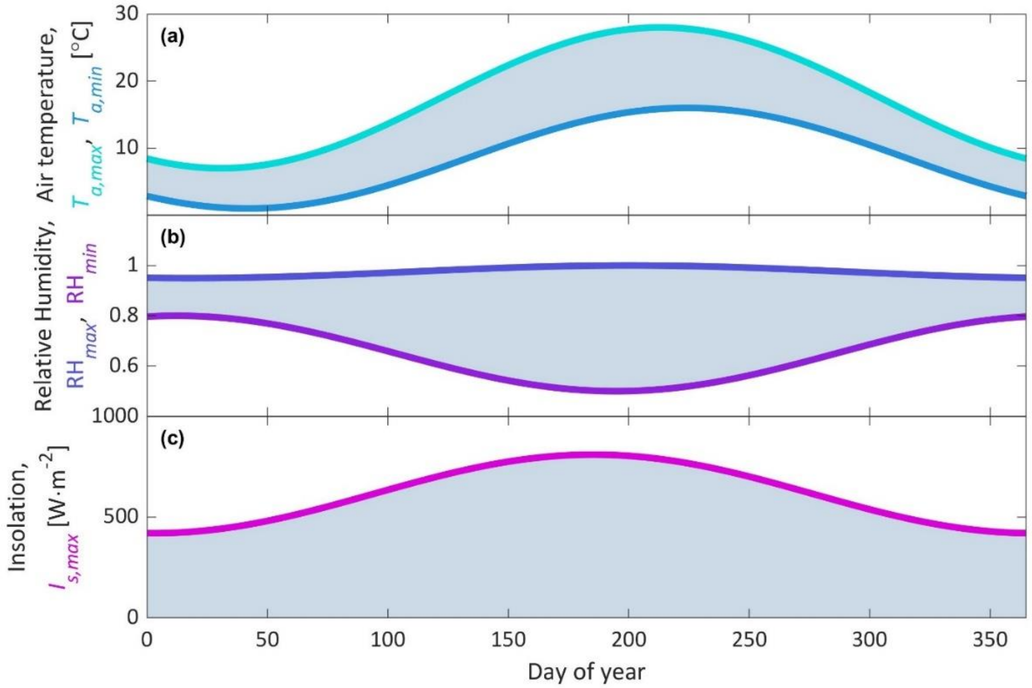

Figure S3: Annual trends in daily minimum and maximum air temperature, relative humidity, and irradiance (e.g., $T_{a,max}$, $T_{a,min}$, RH_{max}, RH_{min}, $I_{s,max}$) used in yearlong and daylong simulations. Environmental signal represents the average trend among years (1948-2018) for Northern Spain (Tillar Valley; Poblet Forest Natural Reserve; Prades Mountains).

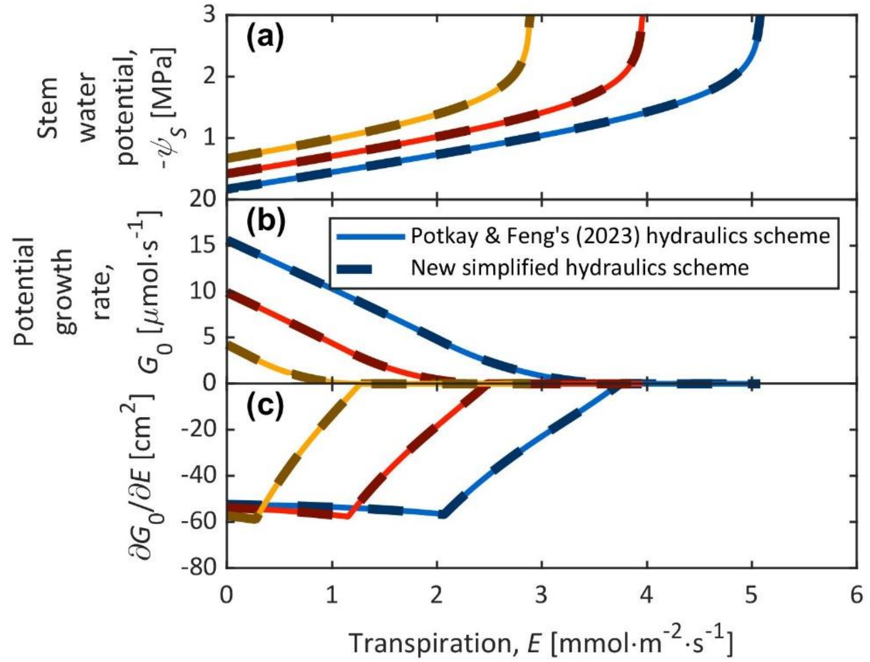

Figure S4: Comparison of new simplified hydraulics scheme with uniform hydraulic stem conductance and Potkay & Feng's original hydraulics scheme with heterogeneous conductance. Thin solid lines show Potkay & Feng's (2023) original predictions, and thick dashed lines show our new predictions. Predictions are shown for the stem water pressure potential, $-\psi_S$ (a), the potential growth rate if NSCs are not limiting, G_0 (where $G \propto G_0$) (b), and $\partial G_0/\partial E$ (where $\chi_w \propto -\partial G_0/\partial E$) (c) for three different soil water potentials, 0 MPa (blue lines), -0.25 MPa (red lines), and -0.5 MPa (gold lines). Simulations were performed with T_a = 25°C for temperature-dependent conductances and the extensibility.

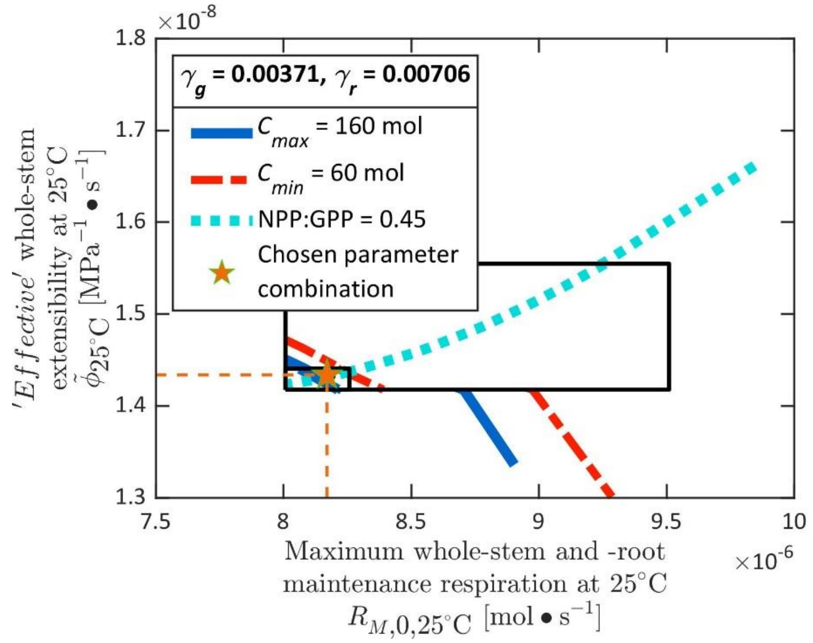

Figure S5: Sensitivity analysis from yearlong simulations with different values of maximum whole-stem and -root maintenance respiration rate at 25°C ($R_{M,0,25°C}$) and '*effective*' whole-stem extensibility at 25°C ($\tilde{\phi}_{25°C}$). Plotted are three isolines for combinations of $R_{M,0,25°C}$ and $\tilde{\phi}_{25°C}$ that produced (1) an annual maximum NSC reserve (C_{max}) of 160 mol, (2) an annual minimum NSC reserve (C_{min}) of 60 mol, and (3) an annual-average NPP:GPP ratio of 0.45 All yearlong simulations were performed with γ_g = 0.00371 and γ_r = 0.00706, which fall within the realistic bounds of values investigated by Jones et al. (2020). The maximum and minimum NSC reserves of 160 mol and 60 mol, respectively, are based on estimates by Schiestl-Aalto et al. (2019) for Scots Pine of approximately the same height as our simulated tree. The NPP:GPP ratio of 0.45 is based on Waring et al. (1998) and Collalti et al. (2020). Black boxes denote regions with different resolutions; resolution increases as search regions becomes smaller. The organge star and orange dashed lines denote our final chosen parameterization for our other simulations (Table 2).

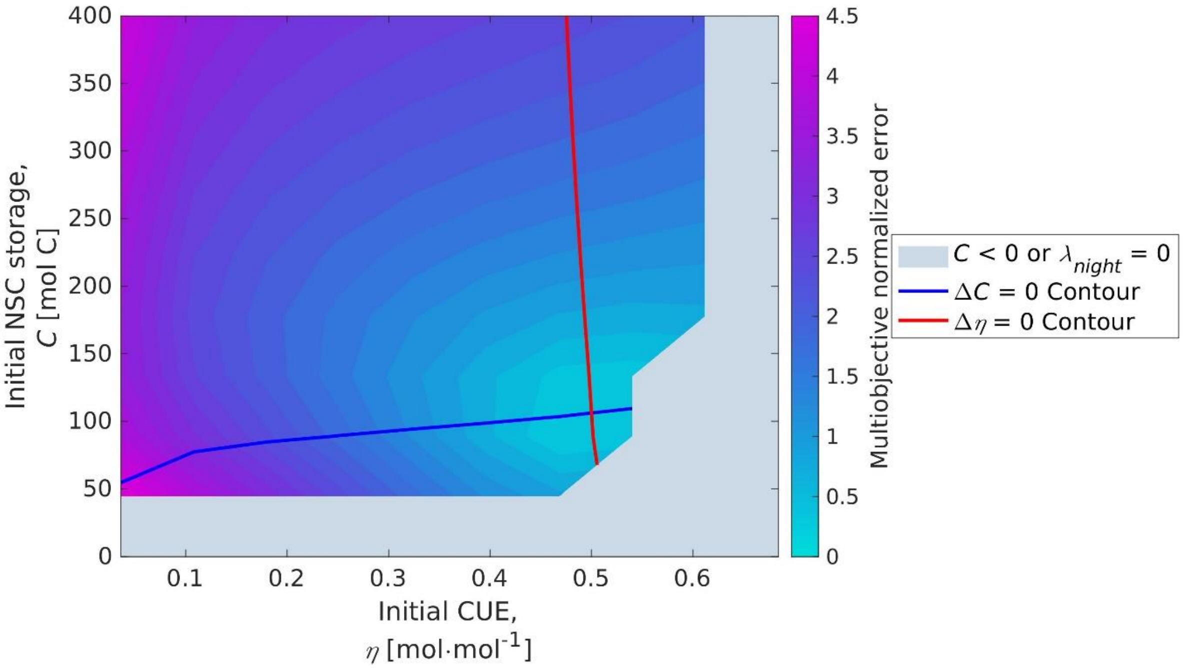

Figure S6: Example of numerical search for optimal transient solution that satisfies cyclicity (i.e., $\Delta\eta = \eta(t_1) - \eta(t_2) = 0$, $\Delta C = C(t_1) - C(t_2) = 0$). We refer to $\Delta\eta$ and ΔC as 'errors.' The *x*- and *y*-axes are the initial NSC and CUE values for a simulation (i.e., $\eta(t_1)$ and $C(t_1)$, respectively). The blue line is the isoline for $\Delta C = 0$, and the red line is the isoline for $\Delta\eta = 0$. Their intersection marks where the combination of initial NSC and CUE values satisfy cyclicity in both C and η. Grey regions are solutions that we did not consider, either due to death by carbon starvation ($C < 0$) or unrealistic nighttime water-use efficiencies ($\lambda_{night} = 0$). The *z*-axis denote the total normalized multi-objective error for both ΔC and $\Delta\eta$ calculated as $(\Delta C_{norm}^2 + \Delta\eta_{norm}^2)^{0.5}$, where ΔC_{norm} and $\Delta\eta_{norm}$ are normalized forms of ΔC and $\Delta\eta$, respectively, $\Delta C_{norm} = \Delta C/\text{std}(\Delta C) - \min(\Delta C/\text{std}(\Delta C)) + \max(\min(\Delta C/\text{std}(\Delta C)), \min(\Delta\eta/\text{std}(\Delta\eta)))$, $\Delta\eta_{norm} = \Delta\eta/\text{std}(\Delta\eta) - \min(\Delta\eta/\text{std}(\Delta\eta)) + \max(\min(\Delta C/\text{std}(\Delta C)), \min(\Delta\eta/\text{std}(\Delta\eta)))$, and min(), max(), and std() are the minimum, maximum, and standard deviation functions, respectively, of all considerer simulations.

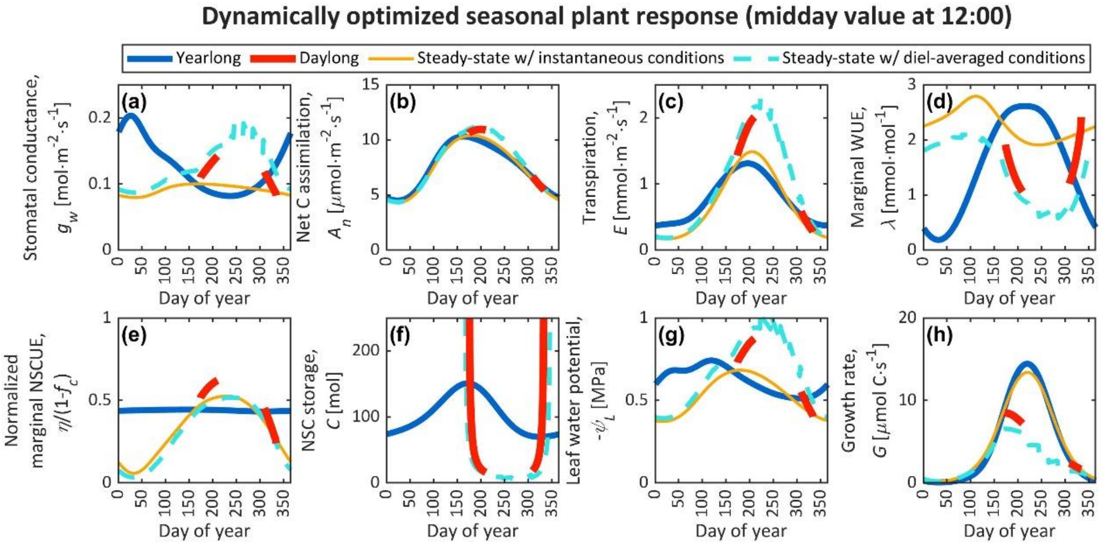

Figure S7: Annual trends from our yearlong simulation (blue lines) in stomatal conductance (g_w), photosynthetic carbon assimilation (A_n), transpiration (E), marginal water-use efficiency (λ), NSC storage (C), growth rate (G), leaf water potentials (ψ_L), and marginal NSC-use efficiency (NSCUE; η) normalized by its maximum value (1-f_c) that maximize growth under the cyclic environmental conditions shown in Fig. S3. We present daily values at noon (12:00). This figure is the same as Fig. 2, except we also present noon-values from successive daylong simulations (red lines), when daylong simulations converged to a result that satisfied the boundary conditions for C and η (i.e., when $\Delta\eta = 0$ and $\Delta C = 0$ isolines intersected; see Fig. S6 for example of intersecting isolines), and noon-values from Potkay & Feng's (2023) steady-state version of the model (gold and cyan lines). For steady-state predictions, simulations were performed twice, first using the instantaneous environmental conditions at noon (gold line) and second correcting the instantaneous conditions to reflect diel-averages (cyan line). Please see Section S3 for mathematical details on this diel-averaged version of Potkay & Feng's (2023) steady-state model.

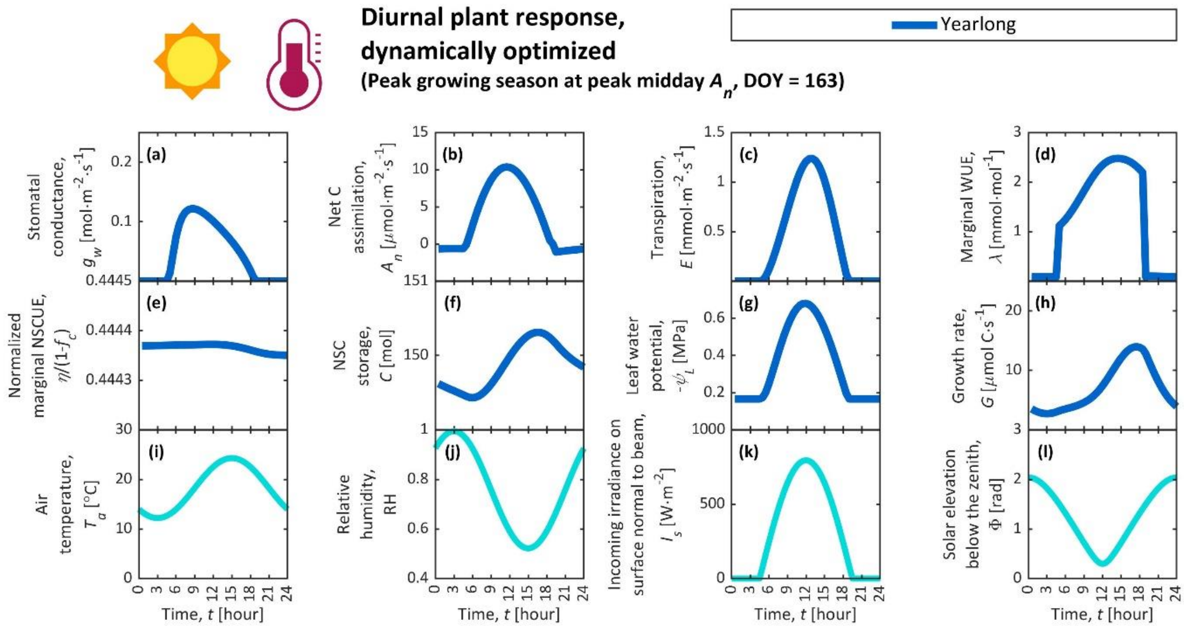

Figure S8: Diel trends from the yearlong simulation during the peak growing season, particularly during peak annual photosynthetic carbon assimilation (A_n) (DOY = 163), including the stomatal conductance (g_w) (a), photosynthetic carbon assimilation (A_n) (b), transpiration (E) (c), marginal water-use efficiency (WUE; λ) (d), marginal NSC-use efficiency (NSCUE; η) normalized by its maximum value (1-f_c) (e), NSC storage (C) (f), leaf water potential (ψ_L) (g), and growth rate (G) (h) as plant responses. Environmental conditions are shown in the bottom row, including trends in air temperature (T_a) (i), relative humidity (RH) (j), incoming irradiance on a surface normal to the beam (I_S) (k), and zenith angle (solar elevation below the zenith; Φ) (l). Daylong simulations for this day (DOY 163) did not converge to a result that satisfied the boundary conditions for C and η (i.e., $\Delta\eta = 0$ and $\Delta C = 0$ isolines do not intersect; see Fig. S6 for example of intersecting isolines).

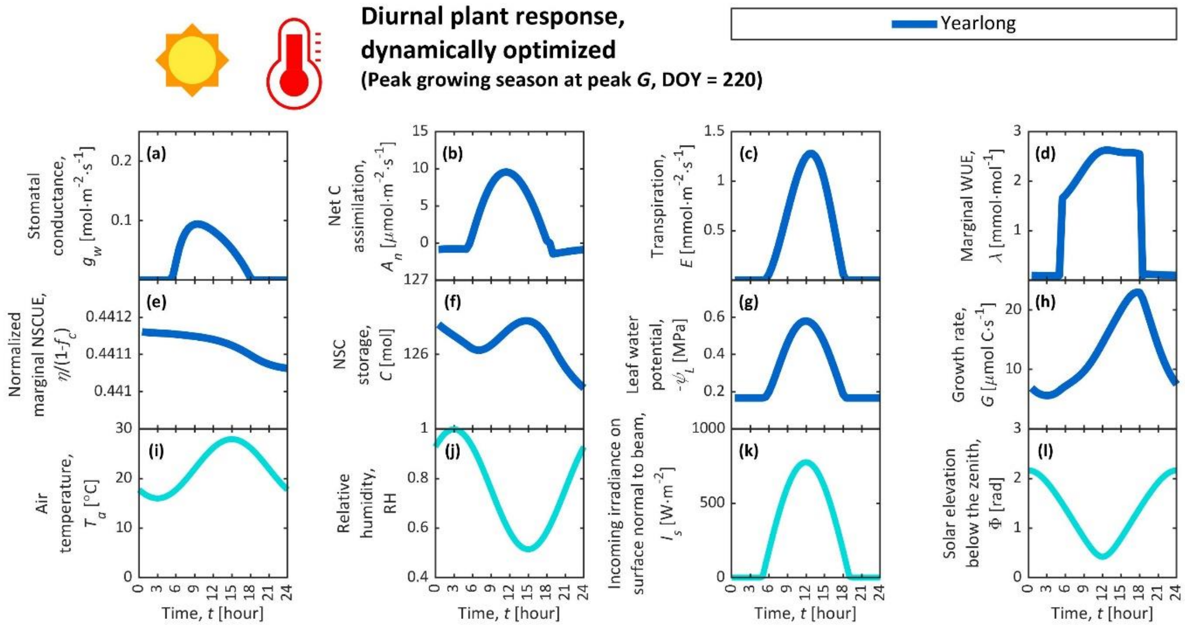

Figure S9: Diel trends from the yearlong simulation during the peak growing season coinciding, particularly during peak woody growth (G) (DOY = 220), including the stomatal conductance (g_w) (a), photosynthetic carbon assimilation (A_n) (b), transpiration (E) (c), marginal water-use efficiency (WUE; λ) (d), marginal NSC-use efficiency (NSCUE; η) normalized by its maximum value ($1\text{-}f_c$) (e), NSC storage (C) (f), leaf water potential (ψ_L) (g), and growth rate (G) (h) as plant responses. Environmental conditions are shown in the bottom row, including trends in air temperature (T_a) (i), relative humidity (RH) (j), incoming irradiance on a surface normal to the beam (I_S) (k), and zenith angle (solar elevation below the zenith; Φ) (l). Daylong simulations for this day (DOY 220) did not converge to a result that satisfied the boundary conditions for C and η (i.e., $\Delta\eta = 0$ and $\Delta C = 0$ isolines do not intersect; see Fig. S6 for example of intersecting isolines).

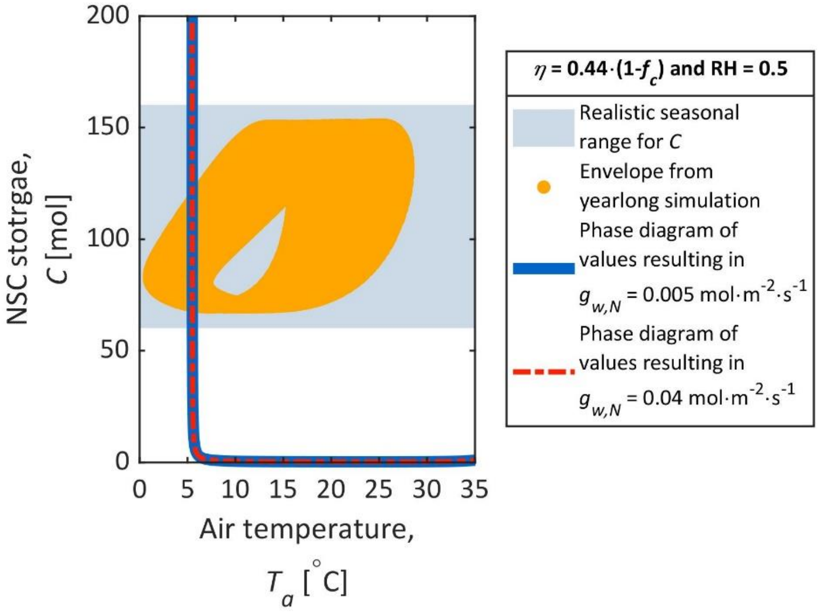

Figure S10: Combinations of NSC storage (C) and air temperature (T_a) that result in realistic values of nocturnal stomatal conductance ($g_{w,N}$), including C-T_a isolines for $g_{w,N}$ = 0.005 mol·m^{-2}·s^{-1} (solid thick blue line) and $g_{w,N}$ = 0.04 mol·m^{-2}·s^{-1} (thin dot-dashed red line). For reference, realistic seasonal ranges in C are shown (gray region), based on estimates by Schiestl-Aalto et al. (2019) for Scots Pine of approximately the same height as our simulated tree. The C-T_a combinations from our yearlong simulation (Fig. 2) are also shown for reference. Nocturnal stomatal conductance values of 0.005 mol·m^{-2}·s^{-1} and 0.04 mol·m^{-2}·s^{-1} were chosen, considering that nocturnal stomatal conductance has been reported to typically be 5-40% of their daytime values (Caird et al., 2007), and assuming a daytime stomatal conductance of 0.1 mol·m^{-2}·s^{-1}, based on mid-growing season values from our yearlong simulation (Fig. 2). Simulations were performed at atmospheric CO_2 and O_2 partial pressures of 410 μmol·mol^{-1} and 207 mmol·mol^{-1}, respectively, RH = 0.5, a solar elevation below the zenith of 0 radians, an incoming irradiance of 0 W·m^{-2}, and constant $\eta = 0.44 \cdot (1 - f_c)$ based on our yearlong simulation (Fig. 2-5), using parameters from Potkay & Feng (2023) and Table 2.

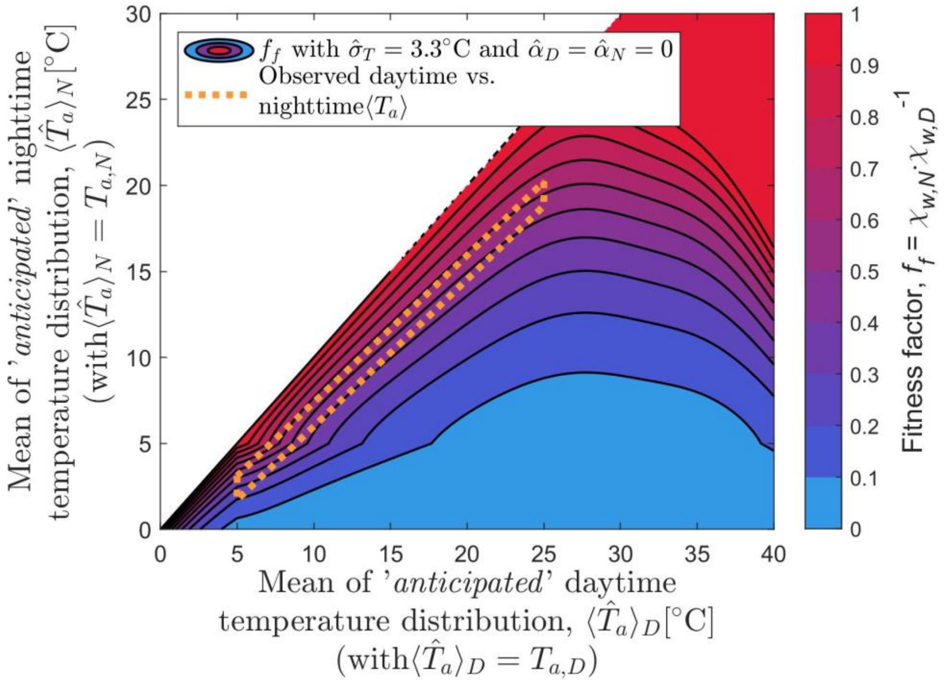

Figure S11: Fitness factor ($f_f = \chi_{w,N}/\chi_{w,D}$) predicted for combinations of varying '*anticipated*' mean daytime and nighttime temperatures ($\langle \hat{T}_a \rangle_D$ and $\langle \hat{T}_a \rangle_N$, respectively), assuming that plants' '*anticipation*' of air temperature is *accurate* (i.e., mean daytime and nighttime temperatures equal the actual daytime and nighttime temperatures, respectively; $\langle \hat{T}_a \rangle_D = T_{a,D}$; $\langle \hat{T}_a \rangle_N = T_{a,N}$). Here, the probabilistic distributions of '*anticipated*' daytime and nighttime temperatures have a standard deviation ($\hat{\sigma_T}$) of 3.3°C and are normally distributed (i.e., zero daytime and nighttime skewness; $\hat{\alpha_D} = \hat{\alpha_N} = 0$), assuming the distributions of '*anticipated*' temperatures are related to the interannual variation of actual temperatures ($\hat{\sigma_T} \approx \sigma_T$; $\hat{\alpha_D} \approx \alpha_D$; $\hat{\alpha_N} \approx \alpha_N$; Fig. S2). For reference, the observed region of actual daytime and nighttime temperatures from our site in Northern Spain (Tillar Valley; Poblet Forest Natural Reserve; Prades Mountains) is outlined (dotted orange line).

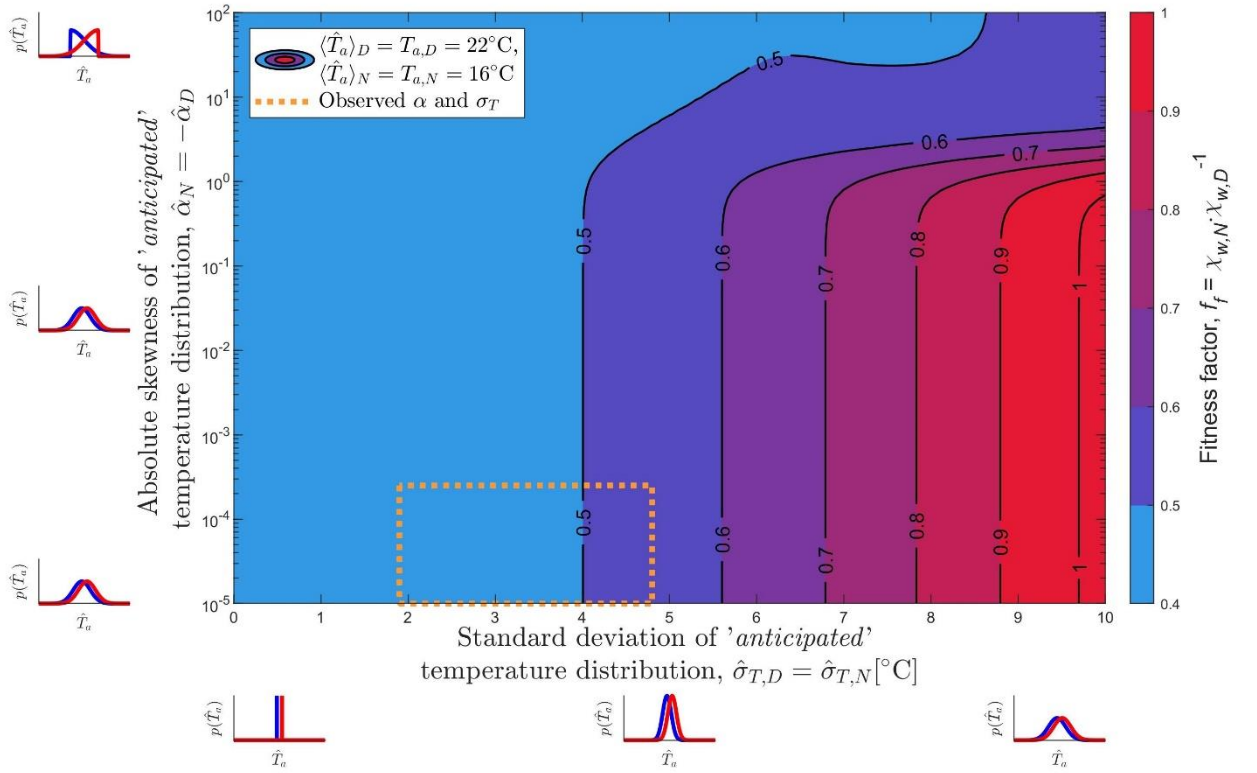

Figure S12: Fitness factor ($f_f = \chi_{w,N}/\chi_{w,D}$) predicted for combinations of varying standard deviations and skewness of the probabilistic distributions of '*anticipated*' daytime and nighttime temperatures ($\hat{\sigma}_{T,D} = \hat{\sigma}_{T,N}$ and $\hat{\alpha}_N = -\hat{\alpha}_D$, respectively) for relatively warm conditions, assuming that plants' '*anticipation*' of air temperature is *accurate* (i.e., mean daytime and nighttime temperatures equal the actual daytime and nighttime temperatures, respectively; $\langle \hat{T}_a \rangle_D = T_{a,D} = 22°C$; $\langle \hat{T}_a \rangle_N = T_{a,N} = 16°$). Daytime and nighttime pairs of actual temperatures were chosen based on their correlation at our site (see dotted orange line in Fig. S11). The *x*-axis is a metric of the plants' *precision* of '*anticipated*' air temperature with perfect *precision* at zero standard deviation and with worse precision at larger standard deviation. Subplots show how the probability distributions of '*anticipated*' daytime (red lines) and nighttime (blue lines) change with standard deviation and skewness. Here, we show results for $\hat{\alpha}_N > 0$ and $\hat{\alpha}_D < 0$, because this combination of $\hat{\alpha}_D$ and $\hat{\alpha}_N$ results in the smallest f_f, all else being equal, and thus these results represent the best-case scenario for producing positive nocturnal stomatal conductance. It is noteworthy, however, that distributions are practically normally-distributed when $|\hat{\alpha}_D| < 1$ and $|\hat{\alpha}_N| < 1$ as shown by subplots of probability distributions. Hence, most of the *y*-axis represents probabilistic distributions that are practically normally-distributed. For reference, the observed range of the standard deviations and skewness of actual temperatures (same as those from Fig. S3) from our site in Northern Spain (Tillar Valley; Poblet Forest Natural Reserve; Prades Mountains) is outlined (dotted orange line).

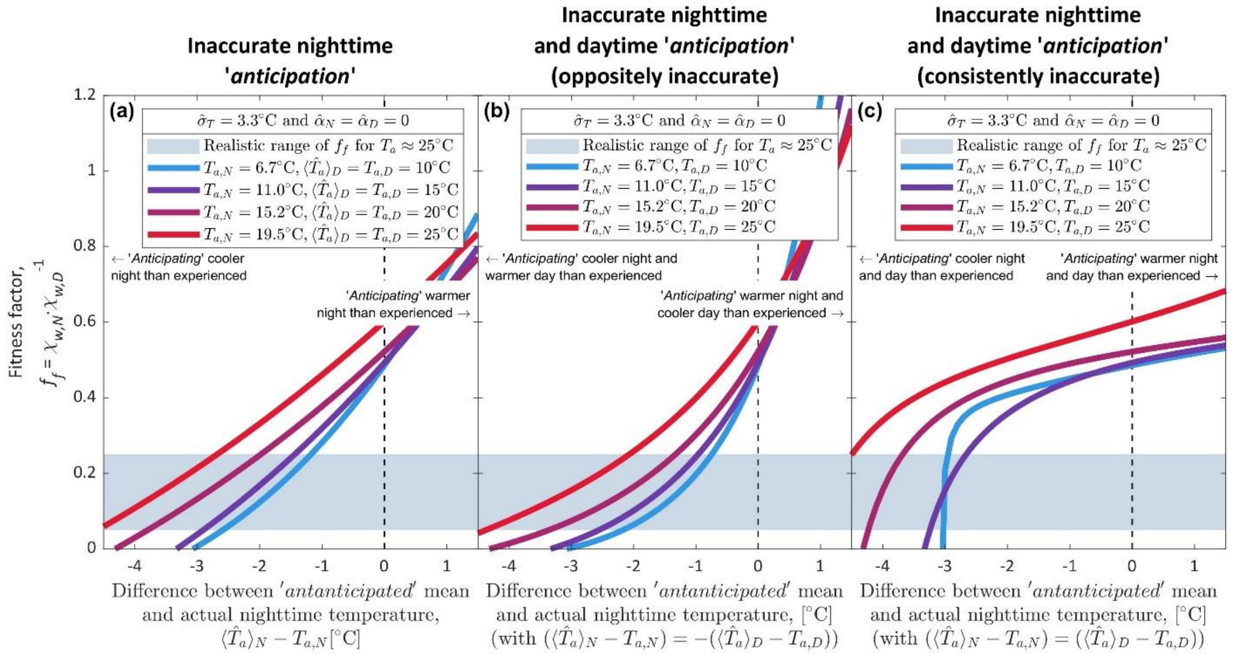

Figure S13: Fitness factor ($f_f = \chi_{w,N}/\chi_{w,D}$) predicted for varying *accuracy* or ability to correctly '*anticipate*' air temperatures under three scenarios: (a) perfectly *accurate* daytime temperature '*anticipation*' ($\langle \hat{T}_a \rangle_D = T_{a,D}$) and *inaccurate* nighttime temperature '*anticipation*', (b) *inaccurate* daytime and nighttime temperature '*anticipation*', where the daytime and nighttime difference between '*anticipated*' and actual temperatures are equal in magnitude and opposite in direction (*oppositely inaccurate*; $[(\langle \hat{T}_a \rangle_N = T_{a,N}] = -[(\langle \hat{T}_a \rangle_D = T_{a,D}])$, and (c) *inaccurate* daytime and nighttime temperature '*anticipation*', where the daytime and nighttime difference between '*anticipated*' and actual temperatures are equal in both magnitude and direction (*consistently inaccurate*; $[(\langle \hat{T}_a \rangle_N = T_{a,N}] = [(\langle \hat{T}_a \rangle_D = T_{a,D}])$. The vertical thin dashed black line indicates perfect *accuracy* in all three subplots. Daytime and nighttime pairs of actual temperatures were chosen based on their correlation at our site (see dotted orange line in Fig. S11). Here, the probabilistic distributions of '*anticipated*' daytime and nighttime temperatures have a standard deviation ($\hat{\sigma_T}$) of 3.3°C and are normally distributed (i.e., zero daytime and nighttime skewness; $\hat{\alpha_D} = \hat{\alpha_N} = 0$), assuming the distributions of '*anticipated*' temperatures are related to the interannual variation of actual temperatures ($\hat{\sigma_T} \approx \sigma_T$; $\hat{\alpha_D} \approx \alpha_D$; $\hat{\alpha_N} \approx \alpha_N$; Fig. S2). For reference, the realistic range of f_f values at 25°C based on our Fig. 1 and Wang et al. (2021) is shown (gray region), and f_f tends to increase with temperature (Wang et al., 2021).

Notes S1 – Description of model changes, environmental forcing and numerical solution, and probabilistic growth maximization

Contents

S1 Model changes

S1.1 Xylem hydraulics

S1.2 Turgor-limited potential growth

S1.3 Nonstomatal limitations to photosynthesis and hydraulically regulated respiration

S2 Environmental forcings and numerical solution

S2.1 Environmental forcing conditions

S2.2 Dynamic simulations

S2.3 Proposed boundary conditions for young trees

S3 Diel-averaged version of Potkay & Feng's (2023) steady-state model

S4 Probabilistic Growth Maximization

S4.1 General framework for probabilistic growth maximization

S4.2 Nocturnal behaviour for probabilistic growth maximization with thermal 'memory'

S1 Model changes

S1.1 Xylem hydraulics

Here, we describe how we model xylem hydraulics (Section S5 in SI of Potkay & Feng, 2023). In the original growth-optimization stomata model (GOSM) (Potkay & Feng, 2023), heterogeneity of xylem conductance within the stem was described due to heterogeneous stem xylem water pressure potentials and conductance loss. This procedure required calculating the polylogarithm function of second order ($\mathrm{Li}_2(x)$) four times (Eq. S6.7-9 in SI of Potkay & Feng, 2023) to calculate the turgor-limited potential growth rate in absence of NSC-limitations (G_0; Section S1.2 below and Section S5 in SI of Potkay & Feng, 2023). $\mathrm{Li}_2(x)$ is solved as either a

power-series with infinite terms or an integral with infinite bounds; in either case, $\mathrm{Li}_2(x)$ is numerically expensive to compute, making the last GOSM inappropriate for dynamic simulation over small time-steps as done here. Here, we present an alternative treatment for stem xylem hydraulics, assuming uniform stem xylem conductance, which is far more computationally inexpensive, and which replaces the stem xylem hydraulic equations presented in Section S5 of Potkay & Feng's (2023) SI. Additionally, we detail our addition of temperature-dependent conductances.

In place of Eq. S5.1 in Potkay and Feng (2023), the steady-state stem xylem hydraulics are given by

$$a_L E = k_S \mathrm{VC}_S(\psi_S)[\psi_{RC} - \psi_S - \varpi\rho g H] , \tag{S1.1.1}$$

where a_L is the leaf area [m^2], E is the leaf-area specific transpiration rate [mol·m^{-2}·s^{-1}], k_S is the maximum conductance of the stem [mol·s^{-1}·MPa^{-1}], ψ_{Rc} and ψ_S are xylem water pressure potentials [MPa] of the root collar and stem, respectively, ϖ is a factor that converts from Pa to MPa (ϖ = 10^{-6} MPa·Pa^{-1}), ρ is the density of water [kg·m^{-3}], g is the acceleration due to gravity [m·s^{-2}], H is the stem height [m], and VC_S is the conductive fraction of the stem which is here modeled as a function of solely by ψ_S,

$$\mathrm{VC}_S(\psi_S) = \frac{1}{1+\exp[-\alpha_S(\psi_S-\beta_S)]}, \tag{S1.1.2}$$

where α_S is a shape parameter [MPa^{-1}], and β_S is the potential at which the stem has lost half of its conductance [MPa]. Conversely, in the original GOSM (Potkay & Feng, 2023), VC_S was spatially-explicit along the stem between the stem apex and the root collar and depended on local stem water pressure potentials. Combining Eq. S1.1.1-2 and rearranging,

$$a_L E = \frac{k_S[\psi_{RC}-\psi_S-\varpi\rho g H]}{1+\exp[-\alpha_S(\psi_S-\beta_S)]} \tag{S1.1.3a}$$

$$\Rightarrow -\alpha_S(\psi_S-\beta_S) = \ln\left[\frac{k_S}{a_L E}(\psi_{RC}-\psi_S-\varpi\rho g H)-1\right] \tag{S1.1.3b}$$

$$\Rightarrow \alpha_S\beta_S + \ln\left(-\alpha_S\frac{a_L E}{k_S}\right) = \alpha_S\psi_S + \ln\left[\frac{k_S}{a_L E}(\psi_{RC}-\psi_S-\varpi\rho g H)-1\right] + \ln\left(-\alpha_S\frac{a_L E}{k_S}\right) \tag{S1.1.3c}$$

$$\Rightarrow \alpha_S\beta_S + \ln\left(-\alpha_S\frac{a_L E}{k_S}\right) = \alpha_S\psi_S + \ln\left[\alpha_S\left(\psi_S-\psi_{RC}+\varpi\rho g H+\frac{a_L E}{k_S}\right)\right] \tag{S1.1.3d}$$

$$\Rightarrow \alpha_S\left(\beta_S + \varpi\rho g H - \psi_{RC} + \frac{a_L E}{k_S}\right) + \ln\left(-\alpha_S \frac{a_L E}{k_S}\right) = \alpha_S\left(\psi_S + \varpi\rho g H - \psi_{RC} + \frac{a_L E}{k_S}\right) + \ln\left[\alpha_S\left(\psi_S + \varpi\rho g H - \psi_{RC} + \frac{a_L E}{k_S}\right)\right] \quad \text{(S1.1.3e)}$$

$$\Rightarrow \ln(\kappa) = \omega + \ln[\omega], \quad \text{(S1.1.3f)}$$

where ω and κ are unitless shorthand for

$$\omega = \alpha_S\left(\psi_S + \varpi\rho g H - \psi_{RC} + \frac{a_L E}{k_S}\right). \quad \text{(S1.1.4)}$$

$$\kappa = -\alpha_S \frac{a_L E}{k_S} \exp\left[\alpha_S\left(\beta_S + \varpi\rho g H - \psi_{RC} + \frac{a_L E}{k_S}\right)\right]. \quad \text{(S1.1.5)}$$

Note that κ is known for a given E, while ω is unknown, since it contains ψ_S, for which we are solving. We introduce the Lambert W function, $W(x)$, as $y = W(x)$ is the solution to the classical problem, $y \cdot \exp(y) = x$. Given that $y \cdot \exp(y) = x$ may be log-transformed into $y + \ln(y) = \ln(x)$, which has the same form as Eq. S1.1.3f, we solve for ω and ψ_S as

$$\omega = W(\kappa) \quad \text{(S1.1.6)}$$

$$\psi_S = \psi_{RC} - \varpi\rho g H - \frac{a_L E}{k_S} + \frac{W(\kappa)}{\alpha_S}, \quad \text{(S1.1.7)}$$

where Eq. S1.1.7 replaces the definition for ψ_S given by Eq. S5.7 in Potkay and Feng (2023). Solving Eq. S1.1.7 requires solving the Lambert W function, $W(x)$, once; however, this computation is ~730 times faster on a personal computer than solving $\mathrm{Li}_2(x)$ four times as done in the previous approach. Fig. S4 shows that this new hydraulics scheme and Potkay and Feng's (2023) original scheme are practically the same. We still solve for ψ_{Rc} though Potkay and Feng's (2023) original Eq. S5.6.

Like in Potkay and Feng (2023), we require a solution for $\partial\psi_S/\partial E$ in place of their Eq. S5.9. Before differentiating Eq. S1.1.6 for $\partial\psi_S/\partial E$, we note an interesting property of the derivative of $W(x)$,

$$\frac{\partial W(x)}{\partial x} = \frac{W(x)}{x[1+W(x)]}. \quad \text{(S1.1.8)}$$

Given Eq. S1.1.8, $\partial\psi_S/\partial E$ may be simply solved by applying the chain-rule to Eq. S1.1.5&7,

$$\frac{\partial\psi_S}{\partial E} = \frac{\partial\psi_{RC}}{\partial E} - \frac{a_L}{k_S} + \frac{1}{\alpha_S}\frac{\partial W(\kappa)}{\partial\kappa}\frac{\partial\kappa}{\partial E} \quad \text{(S1.1.9a)}$$

$$\Rightarrow \frac{\partial\psi_S}{\partial E} = \frac{\partial\psi_{RC}}{\partial E} - \frac{a_L}{k_S} + \frac{1}{\kappa\alpha_S}\frac{W(\kappa)}{1+W(\kappa)}\frac{\partial\kappa}{\partial E} \quad \text{(S1.1.9b)}$$

$$\Rightarrow \frac{\partial\kappa}{\partial E} = k\left[\frac{1}{E} + \alpha_S\left(\frac{a_L}{k_S} - \frac{\partial\psi_{RC}}{\partial E}\right)\right] \quad \text{(S1.1.9c)}$$

$$\Rightarrow \frac{\partial\psi_S}{\partial E} = \left(\frac{\partial\psi_{RC}}{\partial E} - \frac{a_L}{k_S}\right) + \frac{W(\kappa)}{1+W(\kappa)}\frac{1}{\alpha_S E} - \frac{W(\kappa)}{1+W(\kappa)}\left(\frac{\partial\psi_{RC}}{\partial E} - \frac{a_L}{k_S}\right) \quad \text{(S1.1.9d)}$$

$$\Rightarrow \frac{\partial\psi_S}{\partial E} = \frac{\frac{\partial\psi_{RC}}{\partial E} - \frac{a_L}{k_S} + \frac{W(\kappa)}{\alpha_S E}}{1+W(\kappa)} \quad \text{(S1.1.9e)}$$

which may be more simply expressed by rearranging Eq. S1.1.7 for $W(\kappa)$ and plugging it into Eq. S1.1.9e,

$$\frac{\partial\psi_S}{\partial E} = \frac{\frac{\partial\psi_{RC}}{\partial E} - \frac{1}{E}(\psi_{RC} - \psi_S - \varpi\rho g H)}{1 - \alpha_S\left(\psi_{RC} - \psi_S - \varpi\rho g H - \frac{a_L E}{k_S}\right)} = \frac{\frac{\partial\psi_{RC}}{\partial E} - \frac{1}{\mathrm{VC}_S(\psi_S)}\frac{a_L}{k_S}}{1 - \alpha_S\frac{a_L E}{k_S}\left(\frac{1}{\mathrm{VC}_S(\psi_S)} - 1\right)}, \quad \text{(S1.1.10)}$$

which we apply in place for Potkay and Feng's (2023) Eq. S5.9. We still solve for $\partial\psi_{Rc}/\partial E$ though Potkay and Feng's (2023) original Eq. S5.8.

In Potkay and Feng's (2023) GOSM, we treated conductances as independent of temperature. Considering the role of temperature-dependent conductance in explaining stomatal behavior (Lintunen et al., 2020), especially under cold temperatures, we have here formulated leaf, stem, and belowground conductances as temperature-dependent following Q_{10}-type functions. We set the Q_{10} for stem conductance as 1.25, since the temperature-dependence of stem conductance often scales with that of the viscosity of water between 15-30°C (Cochard et al. 2000), which has a Q_{10} of ~1.25. We set the Q_{10} for leaf conductance as 1.60 based on Matzner and Comstock (2001) and Sack et al. (2004). We set the Q_{10} for belowground conductance as 2.20 based on Wan et al. (2001). These Q_{10} values, especially those for leaves and roots, may be specie-specific, and the studies used to parameterize our model were not performed on Scots Pine (Matzner & Comstock, 2001; Wan et al. 2001; Sack et al., 2004), for which the model was otherwise parameterized (Potkay & Feng, 2023). Leaf conductance was formulated as a function of leaf temperature, while stem and belowground conductances were formulated as functions of air temperature. Strictly speaking, belowground conductance should be formulated in terms of soil temperature; we have implicitly assumed that the air-to-soil temperature difference is small, which is on-average the case for North-East Spain (Lembrechts et al., 2022), where our environmental forcing conditions are

based. Values of the maximum stem and belowground conductances at 25°C, $k_{S,25°C}$ and $k_{R,25°C}$, respectively, were set equal the values of the temperature-independent maximum stem and belowground conductances from Potkay and Feng (2023; their k_S and k_R, respectively), given their simulations were performed at 25°C. We set the maximum leaf conductances at 25°C, $k_{L,25°C}$, equal to 1.265 times the value of the temperature-independent maximum leaf conductance from Potkay and Feng (2023; their k_L). The factor of 1.265 was chosen to correct for the fact that the simulated leaf temperature is smaller than the air temperature, while Potkay and Feng's (2023) value for k_L parameter was parameterized for an air-, and not leaf-, temperature of 25°C.

S1.2 Turgor-limited potential growth

Here, we describe changes to how we model the sink-limited potential growth rate if NSCs were not limiting, G_0 (Section S5 in SI of Potkay & Feng, 2023), accounting for the new stem xylem hydraulics scheme presented here (Section S1.2). As in Potkay et al. (2022), we assume that the drop in stem xylem water pressure potentials between the root collar and stem apex is linear. We apply Potkay et al.'s (2022) simpler solution for G_0 (their Eq. 4-5) in place of Potkay & Feng's (2023) complex Eq. S6.7,

$$G_0 = \tilde{\phi}(T_a)\frac{C_W}{u_S}\left[(\psi_S - \pi_0 - \Gamma)(1 - \tilde{z}^+) + (\psi_{Rc} - \psi_S)\left(\frac{1-\tilde{z}^{+2}}{2}\right)\right], \quad \text{(S1.2.1)}$$

where $\tilde{\phi}$ is the temperature-dependent '*effective*' whole-tree extensibility [$MPa^{-1}\cdot s^{-1}$] (see Potkay et al., 2022; Potkay & Feng, 2023), T_a is the air temperature [K], C_W is the woody stem biomass in C equivalents [mol C], u_S is the fraction of growth allocated to woody stem growth, Γ is the threshold pressure that turgor must exceed before tissue expansion occurs [MPa], π_0 is the uniform osmotic potential of the stem if NSCs were not limiting [MPa], the empirical estimation for which from ψ_S remains that same as in Potkay et al. (2022) and Potkay and Feng (2023), and $\tilde{z}^+$ is the relative distance along the stem from the stem apex, normalized by height, at which point growth first ceases,

$$\tilde{z}^{+} = \begin{cases} 1, & \frac{\pi_0+\Gamma-\psi_S}{\psi_{Rc}-\psi_S} > 1 \\ \frac{\pi_0+\Gamma-\psi_S}{\psi_{Rc}-\psi_S}, & 0 \le \frac{\pi_0+\Gamma-\psi_S}{\psi_{Rc}-\psi_S} \le 1. \\ 0, & \frac{\pi_0+\Gamma-\psi_S}{\psi_{Rc}-\psi_S} < 0 \end{cases} \quad \text{(S1.2.2)}$$

Like in Potkay and Feng (2023), we require a solution for $\partial G_0/\partial E$ in place of their Eq. S6.11 given our new equation for G_0. Differentiating Eq. S1.2.1, $\partial G_0/\partial E$ is

$$\frac{\partial G_0}{\partial E} = \tilde{\phi}(T_a)\frac{c_W}{u_S}\begin{Bmatrix} \frac{\partial \psi_S}{\partial E}\left(1-\frac{\partial \pi_0}{\partial \psi_S}\right)(1-\tilde{z}^{+}) - (\psi_S - \pi_0 - \Gamma)\frac{\partial \tilde{z}^{+}}{\partial E} \\ +\left(\frac{\partial \psi_{Rc}}{\partial E} - \frac{\partial \psi_S}{\partial E}\right)\left(\frac{1-\tilde{z}^{+2}}{2}\right) - (\psi_{Rc}-\psi_S)\tilde{z}^{+}\frac{\partial \tilde{z}^{+}}{\partial E} \end{Bmatrix} \quad \text{(S1.2.3a)}$$

$$\Rightarrow \frac{\partial G_0}{\partial E} = \tilde{\phi}(T_a)\frac{c_W}{u_S}\begin{Bmatrix} \frac{\partial \psi_S}{\partial E}\left[\left(1-\frac{\partial \pi_0}{\partial \psi_S}\right)(1-\tilde{z}^{+}) - \left(\frac{1-\tilde{z}^{+2}}{2}\right)\right] + \frac{\partial \psi_{Rc}}{\partial E}\left(\frac{1-\tilde{z}^{+2}}{2}\right) \\ -[\psi_S - \pi_0 - \Gamma + (\psi_{Rc}-\psi_S)\tilde{z}^{+}]\frac{\partial \tilde{z}^{+}}{\partial E} \end{Bmatrix} \quad \text{(S1.2.3b)}$$

$$\Rightarrow \frac{\partial G_0}{\partial E} = \tilde{\phi}(T_a)\frac{c_W}{u_S}\left\{\frac{\partial \psi_S}{\partial E}\left[\left(1-\frac{\partial \pi_0}{\partial \psi_S}\right)(1-\tilde{z}^{+}) - \left(\frac{1-\tilde{z}^{+2}}{2}\right)\right] + \frac{\partial \psi_{Rc}}{\partial E}\left(\frac{1-\tilde{z}^{+2}}{2}\right)\right\}, \quad \text{(S1.2.3c)}$$

where $\partial \psi_S/\partial E$ is given above by Eq. S1.1.10, and $\partial \psi_{Rc}/\partial E$ and $\partial \pi_0/\partial \psi_S$ are given by Potkay and Feng's (2023) Eq. S5.8&S6.12, respectively.

S1.3 Nonstomatal limitations to photosynthesis and hydraulically regulated respiration

In this section, we describe our inclusion of nonstomatal limitations (NSLs) to photosynthesis as well as other changes to how we model net carbon assimilation from the original GOSM (Potkay & Feng, 2023), notably including a hydraulic regulation for leaf dark respiration. By nonstomatal limitations, we refer to increases in mesophyll resistance to CO_2 or decreases in maximum photosynthetic capacities under water stress, both of which inhibit photosynthesis (e.g., Zhou et al., 2016; Novick et al., 2016; Dewar et al., 2018). While increasing mesophyll resistance and declining maximum *true* photosynthetic capacities represent different physiological phenomena, both may be mathematically represented by declines in the *apparent* maximum photosynthetic capacities. Since our GOSM effectively describes the *apparent* maximum photosynthetic capacities (i.e., photosynthesis is calculated from the CO_2 concentration in the mesophyll internal leaf space instead of the CO_2 concentration of the true sites of photosynthesis), we formulate nonstomatal limitations here in terms

of declining *apparent* maximum photosynthetic capacities ($V_{c,max}$, J_{max}; Eq. S4.3-5 in Potkay & Feng, 2023).

In the original GOSM (Potkay & Feng, 2023), the maximum carboxylation rate, $V_{c,max}$, and the biochemical maximum electron transport rate, J_{max}, were treated as functions of only leaf temperature, T_L. To introduce nonstomatal limitations, we treat the values of $V_{c,max}$ and J_{max} at a standard leaf temperature of 25°C, $V_{c,max,25°C}$ and $J_{max,25°C}$, respectively, as functions of leaf water potential, ψ_L,

$$V_{c,max,25℃} = V_{c,max,25℃,0}\varphi_c(\psi_L) \tag{S1.3.1}$$

$$J_{max,25℃} = J_{max,25℃,0}\varphi_j(\psi_L), \tag{S1.3.2}$$

where $V_{c,max,25°C,0}$ and $J_{max,25°C,0}$ are the maximal values of $V_{c,max,25°C}$ and $J_{max,25°C}$, in absence of leaf water stress (ψ_L = 0) [mol·m^{-2}·s^{-1}], and φ_c and φ_j are unitless reduction factors for $V_{c,max}$ and J_{max}, respectively, bound between 0 and 1, both of which are functions of ψ_L. In reality, φ_c and φ_j may be distinct due to differing sensitivities of $V_{c,max}$ and J_{max} to ψ_L; however, we simply treat φ_c and φ_j as identical functions of ψ_L here. Leaf water potentials are calculated by Potkay and Feng's (2023) Eq. S5.11. We set $V_{c,max,25°C,0}$ and $J_{max,25°C,0}$ equal to the values of Potkay and Feng's (2023) parameters for $V_{c,max,25°C}$ and $J_{max,25°C}$, respectively.

In simulations without nonstomatal limitations, φ_c = φ_j = 1, and in simulations with stomatal limitations, we parameterize φ_c and φ_j through a slightly-modified mathematical formulation of Tuzet et al. (2003) and Scots-pine-specific parameters from Nadal-Sala et al. (2021), since we similarly apply Potkay and Feng's (2023) GOSM parameterization for Scots pine,

$$\varphi_c = \frac{1}{1+\exp[a_c^*(\psi_{L,c,50}-\psi_L)]} \tag{S1.3.3}$$

$$\varphi_j = \frac{1}{1+\exp\left[a_j^*(\psi_{L,j,50}-\psi_L)\right]}, \tag{S1.3.4}$$

where a_c^* and a_j^* are shape parameters [MPa^{-1}] for φ_c and φ_j, respectively, and $\psi_{L,c,50}$ and $\psi_{L,j,50}$ are parameters [MPa], representing the ψ_L at which φ_c = ½ (50%) and φ_j = ½ (50%), respectively. Following Nadal-Sala et al.'s (2021) modeling study of Scots pine, we set a_c^* = a_j^* = 2.92 MPa^{-1} and $\psi_{L,c,50}$ = $\psi_{L,j,50}$ = -1.71 MPa. This parameterization predicts that $\varphi_c = \varphi_j \approx$ ⅒ when ψ_L = -2.45 MPa and that $\varphi_c = \varphi_j \approx 0$ when $\psi_L \leq$ -4 MPa.

By altering our scheme for photosynthesis and introducing nonstomatal limitations, we must also derive a new solution for the marginal water-use efficiency (WUE), $\lambda = dA_n/dE$, where A_n is the leaf area-specific net carbon assimilation rate [mol·m^{-2}·s^{-1}] to replace Potkay and Feng's (2023) Eq. S4.11. Similar to Potkay & Feng (2023), we derive λ following similar steps as those of Buckley et al. (2002; their Appendix 2) and Buckley et al. (2017). In fact, this derivation is identical to Potkay and Feng's (2023) derivation for λ, except we must introduce an additional term in their Eq. S4.7 to account for nonstomatal limitations.

As in Potkay and Feng (2023), we define

$$\lambda = \frac{dA_n}{dE} = \frac{\frac{dA_n}{dg_c}}{\frac{dE}{dg_w}\frac{dg_w}{dg_c}}, \tag{S1.3.5}$$

where g_c is the total conductance to CO_2 [mol·m^{-2}·s^{-1}], including stomatal and boundary layer resistances, g_w is the stomatal conductance to H_2O [mol·m^{-2}·s^{-1}], dE/dg_w and dg_w/dg_c are given by Potkay and Feng's (2023) Eq. S3.8 and Eq. S3.10, respectively. The term in the numerator, dA_n/dg_c may be written twice, once through a diffusional supply-based definition of A_n (Eq. S4.1 in Potkay & Feng, 2023), and again by the biochemical demand curve of A_n (Eq. S4.2-4 in Potkay & Feng, 2023),

$$\frac{dA_n}{dg_c} = \frac{\partial A_n}{\partial g_c} + \frac{\partial A_n}{\partial c_i}\frac{dc_i}{dg_c} = k\frac{dc_i}{dg_c} + \xi\frac{dT_L}{dg_w}\frac{dg_w}{dg_c} + \frac{\partial A_n}{\partial \psi_L}\frac{d\psi_L}{dE}\frac{dE}{dg_w}\frac{dg_w}{dg_c} \tag{S1.3.6}$$

where c_i is the unitless mole fraction of the leaf internal air space, k is the is the slope of the biochemical A_n versus c_i demand curve [mol·m^{-2}·s^{-1}] at constant temperature ($k = \partial A_n/\partial c_i$; defined by Eq. S4.12 in Potkay & Feng, 2023), $\xi = \partial A_n/\partial T_L$ (defined by Eq. S4.18 in Potkay & Feng, 2023), and dT_L/dg_w, dg_w/dg_c, $d\psi_L/dE$, and dE/dg_w are given by Potkay and Feng's (2023) Eq. S3.4, Eq. S3.10, Eq. S5.12, and Eq. S3.8, respectively. Note that Eq. S1.3.6 is the same as Potkay and Feng's Eq. S4.7 with the addition of the last group of terms, $\partial A_n/\partial\psi_L \cdot d\psi_L/dE \cdot dE/dg_w \cdot dg_w/dg_c$, which represent the effects of nonstomatal limitations. We rearrange Eq. S1.3.6 for dc_i/dg_c,

$$\frac{dc_i}{dg_c} = \frac{\frac{\partial A_n}{\partial g_c} - \left(\xi\frac{dT_L}{dg_w} + \frac{\partial A_n}{\partial \psi_L}\frac{d\psi_L}{dE}\frac{dE}{dg_w}\right)\frac{dg_w}{dg_c}}{k - \frac{\partial A_n}{\partial c_i}}, \tag{S1.3.7}$$

which, given definitions provided by Potkay and Feng (2023; their Eq. S4.1-4; $\partial A_n/\partial g_c = A_n/g_c$; $\partial A_n/\partial c_i = -g_c$), simplifies to

$$\frac{dc_i}{dg_c} = \frac{1}{k+g_c}\left[\frac{A_n}{g_c} - \left(\xi\frac{dT_L}{dg_w} + \frac{\partial A_n}{\partial\psi_L}\frac{d\psi_L}{dE}\frac{dE}{dg_w}\right)\frac{dg_w}{dg_c}\right]. \tag{S1.3.8}$$

Plugging Eq. S1.3.8 back into Eq. S1.3.6, dA_n/dg_c becomes

$$\frac{dA_n}{dg_c} = \frac{\partial A_n}{\partial g_c} + \frac{\partial A_n}{\partial c_i}\frac{1}{k+g_c}\left[\frac{A_n}{g_c} - \left(\xi\frac{dT_L}{dg_w} + \frac{\partial A_n}{\partial\psi_L}\frac{d\psi_L}{dE}\frac{dE}{dg_w}\right)\frac{dg_w}{dg_c}\right] \tag{S1.3.9a}$$

$$\Rightarrow \frac{dA_n}{dg_c} = \frac{A_n}{g_c} - \frac{g_c}{k+g_c}\left[\frac{A_n}{g_c} - \left(\xi\frac{dT_L}{dg_w} + \frac{\partial A_n}{\partial\psi_L}\frac{d\psi_L}{dE}\frac{dE}{dg_w}\right)\frac{dg_w}{dg_c}\right] \tag{S1.3.9b}$$

$$\Rightarrow \frac{dA_n}{dg_c} = \frac{k}{k+g_c}\frac{A_n}{g_c} + \frac{g_c}{k+g_c}\left(\xi\frac{dT_L}{dg_w} + \frac{\partial A_n}{\partial\psi_L}\frac{d\psi_L}{dE}\frac{dE}{dg_w}\right)\frac{dg_w}{dg_c}. \tag{S1.3.9c}$$

We plug Eq. S1.3.9a into Eq. S1.3.5, simplifying through Potkay and Feng's Eq. S3.4 for dT_L/dg_w, which contains a dE/dg_w term, to define λ as

$$\lambda = \frac{k}{k+g_c}\frac{A_n}{g_c\frac{dE}{dg_w}\frac{dg_w}{dg_c}} + \frac{g_c}{k+g_c}\frac{\xi\frac{dT_L}{dg_w}}{\frac{dE}{dg_w}} + \frac{g_c}{k+g_c}\frac{\partial A_n}{\partial\psi_L}\frac{d\psi_L}{dE} \tag{S1.3.10a}$$

$$\Rightarrow \lambda = \frac{k}{k+g_c}\frac{A_n}{g_c\frac{dE}{dg_w}\frac{dg_w}{dg_c}} - \frac{g_c}{k+g_c}\frac{\xi L}{4\varepsilon\sigma {T_L}^3 + C_p g_b} + \frac{g_c}{k+g_c}\frac{\partial A_n}{\partial\psi_L}\frac{d\psi_L}{dE}, \tag{S1.3.10b}$$

which is identical to the GOSM's previous definition for λ (Eq. S4.11 in Potkay & Feng, 2023), except with the addition of the terms on the far right, $g_c \cdot (k + g_c)^{-1} \cdot \partial A_n/\partial\psi_L \cdot d\psi_L/dE$, which represent nonstomatal limitations. To evaluate the Eq. S1.3.10, we solve for $\partial A_n/\partial\psi_L$ as

$$\frac{\partial A_n}{\partial\psi_L} = \frac{\partial A_n}{\partial V_{c,max}}\frac{dV_{c,max}}{d\psi_L} + \frac{\partial A_n}{\partial J_{max}}\frac{dJ_{max}}{d\psi_L} + \frac{\partial A_n}{\partial R_d}\frac{dR_d}{d\psi_L} \tag{S1.3.11a}$$

$$\Rightarrow \frac{\partial A_n}{\partial\psi_L} = \begin{bmatrix} \frac{\partial A_n}{\partial A_c}\frac{\partial A_c}{\partial V_{c,max}}\frac{dV_{c,max}}{dV_{c,max,25℃}}\frac{dV_{c,max,25℃}}{d\varphi_c}\frac{d\varphi_c}{d\psi_L} \\ + \frac{\partial A_n}{\partial A_j}\frac{\partial A_j}{\partial J}\frac{\partial J}{\partial J_{max}}\frac{dJ_{max}}{dJ_{max,25℃}}\frac{dJ_{max,25℃}}{d\varphi_j}\frac{d\varphi_j}{d\psi_L} - \frac{dR_d}{d\psi_L}\end{bmatrix}, \tag{S1.3.11b}$$

which we simplify through Eq. S1.3.1-2 and definitions given by Potkay and Feng (2023),

$$\frac{\partial A_n}{\partial\psi_L} = \begin{bmatrix} \frac{\partial A_n}{\partial A_c}\frac{A_c+R_d}{V_{c,max,25℃}}V_{c,max,25℃,0}\frac{d\varphi_c}{d\psi_L} - \frac{dR_d}{d\psi_L} \\ + \frac{\partial A_n}{\partial A_j}\frac{A_j+R_d}{J}\frac{\partial J}{\partial J_{max}}\frac{J_{max}}{J_{max,25℃}}J_{max,25℃,0}\frac{d\varphi_j}{d\psi_L}\end{bmatrix} \tag{S1.3.12a}$$

$$\Rightarrow \frac{\partial A_n}{\partial\psi_L} = \frac{\partial A_n}{\partial A_c}\frac{A_c+R_d}{\varphi_c(\psi_L)}\frac{d\varphi_c}{d\psi_L} + \frac{\partial A_n}{\partial A_j}\frac{A_j+R_d}{J}\frac{\partial J}{\partial J_{max}}\frac{J_{max}}{\varphi_j(\psi_L)}\frac{d\varphi_j}{d\psi_L} - \frac{dR_d}{d\psi_L} \tag{S1.3.12d}$$

$$\Rightarrow \frac{\partial A_n}{\partial \psi_L} = \frac{\partial A_n}{\partial A_c}(A_c + R_d)\frac{d\ln(\varphi_c)}{d\psi_L} + \frac{\partial A_n}{\partial A_j}(A_j + R_d)\frac{\partial J}{\partial J_{max}}\frac{J_{max}}{J}\frac{d\ln(\varphi_j)}{d\psi_L} - \frac{dR_d}{d\psi_L} \quad \text{(S1.3.12d)}$$

$$\Rightarrow \frac{\partial A_n}{\partial \psi_L} = \frac{\partial A_n}{\partial A_c}(A_c + R_d)a_c^*(1-\varphi_c) + \frac{\partial A_n}{\partial A_j}(A_j + R_d)\frac{\partial J}{\partial J_{max}}\frac{J_{max}}{J}a_j^*(1-\varphi_j) - \frac{dR_d}{d\psi_L}, \text{(S1.3.12d)}$$

where $\partial A_n/\partial A_c$, $\partial A_j/\partial A_c$, and $\partial J/\partial J_{max}$ are given by Potkay and Feng's (2023) Eq. S4.13, Eq. S4.15, and Eq. S4.19, respectively. Modeling of A_c and A_j are detailed in Potkay and Feng's (2023) Section S4 of their SI., though now including the nonstomatal limitations presented here (Eq. S1.3.1-4).

We treat R_d as a function of T_L and ψ_L (i.e., $dR_d/d\psi_L > 0$) unlike in Potkay and Feng (2023; Section S4 of their SI), who did not include a functional dependence of R_d on ψ_L. To parameterize the role of water-stress on leaf respiration, we model R_d as a function of leaf water content instead of leaf water potential, since empirical relationships between R_d and water content (or its corollary, turgor) are often reported in the literature (Flexas et al., 2005; Galmés et al., 2007; Saveyn et al., 2007; Huang et al., 2020). We refer to this dependence of R_d on leaf water content as '*hydraulically regulated respiration*' (HRR). We model R_d as

$$R_d(T_L, \mathrm{RWC}_L(\psi_L)) = R_{d,0}(T_L)\exp[b_r^*(\mathrm{RWC}_L(\psi_L) - 1)], \quad \text{(S1.3.13)}$$

where $R_{d,0}$ is the leaf area-specific dark respiration rate at full hydration [$\mathrm{mol\cdot m^{-2}\cdot s^{-1}}$], RWC_L is the leaf relative water content ($0 \leq \mathrm{RWC}_L \leq 1$), and b_r^* is an unitless, empirical shape parameter relating RWC_L to R_d. In most simulations, we set $b_r^* = 0$, ignoring the relationship between RWC_L and R_d. We set $b_r^* = 2$ as an intermediate value from the parameter's range ($\sim 0 \leq b_r^* \leq \sim 5$), which we estimated by fitting Eq. S1.3.13 to the positively-correlated portion of RWC_L-R_d data ($\mathrm{RWC}_L > 0.6$) from Flexas et al. (2005) and Galmés et al. (2009). Similar to Potkay and Feng (2023), we model $R_{d,0}$ as a function of leaf temperature through its proportionality with the maximum carboxylation rate at a leaf temperature of 25°C, $V_{c,max,25°\mathrm{C}}$, according to de Pury and Farquhar (1997) and a Q_{10}-type relationship,

$$R_{d,0}(T_L) = 0.01 \cdot V_{c,max,25°\mathrm{C},0} Q_{10}^{\frac{T_L - 25°\mathrm{C}}{10°\mathrm{C}}}, \quad \text{(S1.3.14)}$$

where $Q_{10} = 2$. This formulation for R_d (Eq. S1.3.13-14) is identical to Potkay and Feng's (2023) formulation when ignoring the RWC_L-R_d relationship (i.e., $b_r^* = 0$).

We solve the $dR_d/d\psi_L$ term in Eq. S1.3.11-12 as

$$\frac{dR_d}{d\psi_L} = \frac{\partial R_d}{\partial \mathrm{RWC}_L}\frac{d\mathrm{RWC}_L}{d\psi_L}, \tag{S1.3.15}$$

where $\partial R_d/\partial \mathrm{RWC}_L$ is found by differentiating Eq. S1.3.13,

$$\frac{\partial R_d}{\partial \mathrm{RWC}_L} = b_r^* R_{d,0}(T_L)\exp[b_r^*(\mathrm{RWC}_L(\psi_L) - 1)] = b_r^* R_d\big(T_L, \mathrm{RWC}_L(\psi_L)\big). \tag{S1.3.16}$$

We model RWC_L and its relationship to ψ_L through pressure-volume curves (Bartlett et al., 2012). We emphasize that pressure-volume curves assume that change in osmotic potential occur from solely dilution of a constant number of moles of solutes, and thus this approach ignores acclimation of osmolyte concentrations and osmotic potentials. Given this simplification, RWC_L may be defined as (Bartlett et al., 2019)

$$\mathrm{RWC}_L(\psi_L) = \begin{cases} \frac{\psi_L+\pi_{L,0}+\varepsilon_L}{2\varepsilon_L} + \sqrt{\left(\frac{\psi_L+\pi_{L,0}+\varepsilon_L}{2\varepsilon_L}\right)^2 - \frac{\pi_{L,0}}{\varepsilon_L}}, & \psi_L > \pi_{L,tlp} \\ \frac{\pi_{L,0}}{\psi_L}, & \psi_L \le \pi_{L,tlp} \end{cases}, \tag{S1.3.17}$$

where $\pi_{L,0}$ is the osmotic potential at full hydration [MPa], ε_L is the bulk leaf modulus of elasticity [MPa], and $\pi_{L,tlp} = \pi_{L,0}\cdot\varepsilon_L\cdot(\pi_{L,0} + \varepsilon_L)^{-1}$ is the leaf turgor loss point [MPa]. Given that our model is parameterized for Scots Pine in a Mediterranean climate, we set $\pi_{L,0}$ = -2 MPa, ε_L = 14 MPa, and thus $\pi_{L,tlp}$ = -2.33 MPa, reflecting some of the most drought-resistant values from the natural range in $\pi_{L,0}$ and ε_L for temperate conifers (Bartlett et al., 2012). We find the $d\mathrm{RWC}_L/d\psi_L$ term in Eq. S2.3.15 by differentiating Eq. S1.3.17,

$$\frac{d\mathrm{RWC}_L}{d\psi_L} = \begin{cases} \frac{1}{2\varepsilon_L}\left(1 + \frac{\psi_L+\pi_{L,0}+\varepsilon_L}{\sqrt{(\psi_L+\pi_{L,0}+\varepsilon_L)^2 - 4\varepsilon_L\pi_{L,0}}}\right), & \psi_L > \pi_{L,tlp} \\ -\frac{\pi_{L,0}}{\psi_L{}^2}, & \psi_L \le \pi_{L,tlp} \end{cases}. \tag{S1.3.18}$$

S2 Environmental forcings and numerical solution

S2.1 Environmental forcing conditions

As discussed in the main text, we present plant responses for environmental conditions that are cyclic. Atmospheric conditions are based on NOAA reanalysis data (NCEP/NCAR Reanalysis 1; Kalnay et al. 1996) for forest stands in Northern Spain (Tillar Valley; Poblet Forest Natural Reserve; Prades Mountains). We treated environmental conditions as annually cyclic and

continuous by fitting sinusoidal functions to temporal trends in T_a, RH, and I_s with diel and annual periods. It is noteworthy that the diel variation in I_s is described by a $\max(\cos(x), 0)$ function, where $\max(x, y)$ is the maximum of x or y and $\cos(x)$ is the cosine function, instead of true sinusoidal function, such that I_s is zero at night (see Eq. S2.1.12 below). First, we decomposed the time of observed environmental conditions, t, into year (Y), the day of year (DOY) and hour of day (HOD), where Y is an integer (1948-2018), DOY is an integer between 1 and 365, and HOD is continuous between 0 and 24. We write

$$T_a(t) = T_a(\mathrm{Y}, \mathrm{DOY}, \mathrm{HOD}) \tag{S2.1.1}$$

$$\mathrm{RH}(t) = \mathrm{RH}(\mathrm{Y}, \mathrm{DOY}, \mathrm{HOD}) \tag{S2.1.2}$$

$$I_s(t) = I_s(\mathrm{Y}, \mathrm{DOY}, \mathrm{HOD}). \tag{S2.1.3}$$

For annual cyclicity, we effectively removed the dependence of T_a, RH, and I_s by averaging yearlong trends in T_a, RH, and I_s from the reanalysis data among years (1948-2018) at subdaily increments. The interannual average environmental conditions at a single DOY and HOD are

$$\langle T_a\rangle(t) = \langle T_a\rangle(\mathrm{DOY}, \mathrm{HOD}) = \frac{\int_{1948}^{2018} T_a(\mathrm{Y}', \mathrm{DOY}, \mathrm{HOD})\, d\mathrm{Y}'}{2018-1948} \tag{S2.1.4}$$

$$\langle \mathrm{RH}\rangle(t) = \langle \mathrm{RH}\rangle(\mathrm{DOY}, \mathrm{HOD}) = \frac{\int_{1948}^{2018} T_a(\mathrm{Y}', \mathrm{DOY}, \mathrm{HOD})\, d\mathrm{Y}'}{2018-1948} \tag{S2.1.5}$$

$$\langle I_s\rangle(t) = \langle I_s\rangle(\mathrm{DOY}, \mathrm{HOD}) = \frac{\int_{1948}^{2018} T_a(\mathrm{Y}'', \mathrm{DOY}, \mathrm{HOD})\, d\mathrm{Y}'}{2018-1948} \tag{S2.1.6}$$

For T_a, we also fit skewed normal distributions to evaluate the standard deviation and skewness of air temperature (Fig. S2), which we considered later in a probabilistic view of growth maximization (Section S4). These skewed normal distributions have the form (O'hagan & Leonard, 1976),

$$p_T(T_a, t) = \frac{\sqrt{1-\beta(\alpha(t))^2}}{\sigma_T(t)\sqrt{2\pi}} \exp\left\{-\frac{1}{2}\left[\sqrt{1-\beta(\alpha(t))^2}\,\frac{T_a-\langle T_a\rangle(t)}{\sigma_T(t)} + \beta(\alpha(t))\right]^2\right\}\left\{1 + \mathrm{erf}\left[\frac{\alpha(t)}{\sqrt{2}}\left(\sqrt{1-\beta(\alpha(t))^2}\,\frac{T_a-\langle T_a\rangle(t)}{\sigma_T(t)} + \beta(\alpha(t))\right)\right]\right\} \tag{S2.1.7}$$

where σ_T is the standard deviation of environmental conditions among years at a single DOY and HOD,

$$\sigma_T(t) = \sigma_T(\text{DOY}, \text{HOD}) = \sqrt{\frac{\int_{1948}^{2018} [T_a(\text{Y}', \text{DOY}, \text{HOD}) - \langle T_a \rangle(\text{DOY}, \text{HOD})]^2 \, d\text{Y}'}{2018 - 1948}} \tag{S2.1.8}$$

$\text{erf}[(x)]$ is the error function, α is a time-specific shape parameter controlling the skew, and $\beta(\alpha(t))$ is shorthand for

$$\beta\big(\alpha(t)\big) = \alpha(t)\sqrt{\frac{2}{\pi(1+\alpha(t)^2)}}. \tag{S2.1.9}$$

Then, from these $\langle T_a \rangle$, $\langle \text{RH} \rangle$, and $\langle I_s \rangle$ for each HOD, we determined the maximum and minimum diel conditions (referred to as $T_{a,max}$, $T_{a,min}$, RH_{max}, RH_{min}, $I_{s,max}$; Fig. S3). We determined $T_{a,max}$, $T_{a,min}$, RH_{max}, RH_{min}, and $I_{s,max}$ by fitting the following functions for each DOY,

$$\langle T_a \rangle(\text{DOY}, \text{HOD}) = \frac{T_{a,max}(\text{DOY}) + T_{a,min}(\text{DOY})}{2} + \frac{T_{a,max}(\text{DOY}) - T_{a,min}(\text{DOY})}{2} \sin\left(\pi \frac{\text{HOD} - \varphi_T}{12 \text{ hr.}}\right) \tag{S2.1.10}$$

$$\langle \text{RH} \rangle(\text{DOY}, \text{HOD}) = \frac{\text{RH}_{max}(\text{DOY}) + \text{RH}_{min}(\text{DOY})}{2} + \frac{\text{RH}_{max}(\text{DOY}) - \text{RH}_{min}(\text{DOY})}{2} \sin\left(\pi \frac{\text{HOD} - \varphi_{\text{RH}}}{12 \text{ hr.}}\right) \tag{S2.1.11}$$

$$\langle I_s \rangle(\text{DOY}, \text{HOD}) = I_{s,max}(\text{DOY}) \max\left[\cos\left(\pi \frac{\text{HOD} - 12 \text{ hr.}}{\text{DL}(\text{DOY})}\right), 0\right], \tag{S2.1.12}$$

where φ_T and φ_{RH} are fitted DOY-specific phases shifts in hours, and DL is the DOY-specific daylength in hours and is the number of hours that irradiance is positive. Instead of fitting DL, we determined it from the diel pattern of zenith angles (Φ) calculated from the latitude of our site (40°N), HOD, and the declination of the Sun (approximated by $-0.4091 \cdot \cos\left(2\pi \frac{\text{DOY}+10}{365}\right)$ radians), ignoring right ascension. Specifically, DL was calculated as the duration over which $\Phi \leq ½\cdot\pi$. Notably, in Eq. S2.1.12, we have assumed that the diel maximum of $\langle I_s \rangle$ occurs always at noon. Unless otherwise noted, we fixed ψ_{soil} at 0 MPa for simplicity and because our current growth optimization does not consider how plants optimally use soil-water over a dry down (i.e., *water-use strategies*; Manzoni et al., 2013; Mrad et al., 2019). Lastly, to describe intra-annual variation in maximum and minimum diel conditions, we fit sinusoidal functions to $T_{a,max}$, $T_{a,min}$, RH_{max}, RH_{min}, I_s, (Fig. S3) similar to those of Eq. S2.10-11, except with periods of 365 days instead of 24 hr.

S2.2 Dynamic simulations

Dynamic solutions of plant responses to cyclic environmental signals may be either *cyclic*, *stable*, or *unstable*. *Stable* simulations are not cyclic over the first period of the cyclic

environmental signals; nonetheless, they converge over multiple periods onto the *cyclic* solution. Conversely, *unstable* solutions diverge from the cyclic solution over multiple periods, especially as η approaches either of its limits (0 or $1 - f_c$, since $\dot{\eta} \leq 0$ when $\eta \leq 0$ and $\dot{\eta} > 0$ when $\eta \geq 1 - f_c$ by Eq. 4), leading to unrealistic stomatal responses. Here, we present *cyclic* solutions. For a given environmental signal, we numerically searched for the combination of initial η and C values (i.e., $\eta(t_1)$, $C(t_1)$) that assured their cyclicity between the initial time, t_1, and the timescale of optimization, t_2 (i.e., $\eta(t_1) = \eta(t_2)$ and $C(t_1) = C(t_2)$). We performed this search by plotting contour maps of $\Delta\eta = \eta(t_1) - \eta(t_2)$ and $\Delta C = C(t_1) - C(t_2)$ as function of $\eta(t_1)$ and $C(t_1)$ (,). We performed this process iteratively, plotting contours at progressively finer resolutions in $\eta(t_1)$-$C(t_1)$ space near the intersection of the $\Delta\eta = 0$ and $\Delta C = 0$ isolines calculated by the previous iteration. We stopped the search once it found a solution with $|\Delta\eta| < 10^{-6}$ mol·mol^{-1} and $|\Delta C| < 10^{-4}$ mol.

In our numerical search, we disregarded solutions in which NSCs became entirely depleted (i.e., carbon starvation; $C < 0$). Similarly in daylong simulations, we disregarded simulations in which the marginal carbon cost of water (χ_w; Potkay & Feng, 2023) equaled 0 when $A_n < 0$ (e.g., nighttime). We disregarded the latter case, because when $A_n < 0$, there is no g_w allowing $\lambda = 0$ by the GOSM. At nighttime, A_n approximately equals the leaf dark respiration (R_d) (Wang et al., 2021). Due to our GOSM's monotonic Q_{10}-type relationship between R_d and leaf temperature (T_L), there is no g_w allowing $\chi_w = \lambda = 0$ when $A_n \approx R_d$, since nighttime $\lambda > 0$ (Fig. 1), except in the limit as E approaches ∞, where T_L, R_d, and dR_d/dE approach $-\infty$, 0, and 0, respectively. Here, we have also modeled R_d as an increasing monotonic exponential function of leaf relative water content, RWC_L (Eq. S1.3.13), based on observed empirical relationships between respiration and tissue water content (Flexas et al., 2005; Galmés et al., 2007; Saveyn et al., 2007; Huang et al., 2020). This additional dependence of R_d on RWC_L modifies nighttime λ; however, nighttime λ nonetheless remains positive ($\lambda > 0$; Fig. 1). Some studies report a bimodal, nonmonotonic relationship between R_d and RWC_L, in which R_d initially declines under leaf desiccation and then rises with further desiccation (Flexas et al., 2005; Galmés et al., 2007). If we included this nonmonotonic relationship between R_d and RWC_L, then we would be able to produce nighttime $\lambda = 0$. However, the RWC_L at which R_d is minimum tends to range between ~60% to ~70% (Flexas et al., 2005; Galmés et al., 2007), which is less than the turgor loss point of most plants (Fig. S1; Bartlett et al., 2012). Hence, modeling more complex, nonmonotonic RWC_L-R_d formulations would not be entirely satisfying. We allowed simulations predicting $\chi_w = 0$ when $A_n > 0$ (e.g.,

daytime), because we consider both of the effects of evaporative cooling and nonstomatal limitations (NSLs; Eq. S1.3.1-2) on photosynthesis. In the absence of nonstomatal limitations, evaporative cooling reduces the carboxylation- and electron transport-limited rates of A_n, producing a maximal A_n with respect to E, though often at unrealistically large E (Potkay & Feng, 2012). Additionally, we consider nonstomatal limitations caused by changes in *apparent* photosynthetic capacities under leaf water stress (Section S1.3), since they are compatible with traditional AOH theories (Zhou et al., 2013; Novick et al., 2016), and since Potkay and Feng (2023) recognized their importance to realistic predictions of stomatal conductance under the GOH framework when stems cannot grow (G = 0). These additional limitations enable improve predictions of E when χ_w = 0 during daytime (Dewar et al., 2018). In numerical searches for yearlong cyclic solutions, we included simulations in which χ_w equaled 0 when $A_n < 0$, because of cold nighttime winter temperatures (Fig. S1), which made $\chi_w = 0$ unavoidable due to the effects of cold temperature on G and thus also χ_w (i.e., $\chi_w = 0$ when $T_a \leq 5$°C by the temperature-dependence of G and thus also $\partial G/\partial E$ in Eq. 3; Fig. S1 in Potkay & Feng, 2023). In this case, we simply set g_w = 0.

S2.3 Proposed boundary conditions for young trees

Simulating young trees is beyond the scope of this study and would require non-cyclic multi-year simulations. Boundary conditions at the end of the *timescale* (t_2) would not need to be defined; however, initial conditions (for C and η) at the time of sprouting (t_1) would need to be defined. The initial condition for C could be estimated empirically, and the initial condition for η could be treated as its maximum value ($1 - f_c$), which would agree with the common notion that the growth of younger trees is more source-limited than that of older trees (e.g., Brienen et al., 2022) and with observations of increasing hydraulic costs with size (e.g., Ryan et al., 2006). Over many years, these non-cyclic simulations would converge onto the cyclic simulation as relative size changes diminished.

S3 Diel-averaged version of Potkay & Feng's (2023) steady-state model

In Fig. S7, we present predictions of Potkay and Feng's (2023) steady-state model for two configurations. In both configurations, whole-tree NSCs and the NSC-use efficiency (C and η, respectively) are assumed in steady-state and constant within each individual DOY. Though constant within a DOY, C and η vary among days, because the model is applied to each DOY individually (i.e., there are 365 separate simulations to describe an entire year). The first configuration applies the instantaneous noon environmental conditions to predict the steady-state marginal carbon cost of water as described in Potkay and Feng (2023; their Eq. 11),

$$\bar{\chi}_w = \frac{1}{a_L}\left|\frac{\partial \bar{G}}{\partial \bar{E}}\right| \frac{\frac{\partial \bar{R}_M}{\partial \bar{C}}}{\frac{\partial \bar{G}}{\partial \bar{C}}}, \tag{S3.1.1}$$

where vinculum (e.g., $\bar{\chi}_w$, $\bar{C}$) denote steady-state values (i.e., when $d\bar{\eta}/dt = 0$ and $d\bar{C}/dt = a_L \cdot \bar{A}_n - \bar{R}_M(\bar{C}) - \bar{G}(\bar{C})/(1 - f_c) = 0$), and here all of the terms in Eq. S2.1.1 are evaluated for the environmental conditions at noon for each DOY. This steady-state assumption is unsatisfying, particularly for predictions of whole-plant NSC (i.e., $\bar{C}$), because whole-canopy noon photosynthetic carbon assimilation exceeds the NSC-unlimited whole-plant carbon demand (i.e., $a_L \cdot \bar{A}_n > R_M(C = \infty) + G(C = \infty)/(1 - f_c)$) for most of the year, leading to predictions of infinite C over the growing season (Fig. S7). To predict finite $\bar{C}$, we also present results of a modified version of Potkay and Feng's (2023) in which the NSC balance is calculated in steady-state over an individual DOY in place of Eq. 2 in the main text,

$$\frac{d\bar{C}}{dt} = a_L\langle \bar{A}_n \rangle - \langle \bar{R}_M \rangle - \frac{\langle \bar{G} \rangle}{1 - f_c} = 0, \tag{S3.1.2}$$

where the $\langle x \rangle$ operator denotes an ensemble average over a DOY (same notation as in Section S4). By replacing Eq. 2 with Eq. S3.1.2, the steady-state marginal carbon cost of water for this alternative steady-state model is

$$\bar{\chi}_w = \frac{1}{a_L}\left|\frac{\partial \langle \bar{G} \rangle}{\partial \bar{E}}\right| \frac{\frac{\partial \langle \bar{R}_M \rangle}{\partial \bar{C}}}{\frac{\partial \langle \bar{G} \rangle}{\partial \bar{C}}} \tag{S3.1.3}$$

instead of Eq. S2.1.1. We approximated $\langle A_n \rangle$, $\langle R_M \rangle$, and $\langle G \rangle$ for a given DOY as functions of that DOY's instantaneous noon values of A_n, R_M, and G, the day length, and diel variations in temperature,

$$\langle A_n \rangle \approx f_D f_A A_{n,noon} - (1 - f_D) c_{R,N} R_{d,noon,NE} \tag{S3.1.4}$$

$$\langle R_M \rangle \approx [f_D c_{R,D} + (1 - f_D) c_{R,N}] R_{M,noon} \quad \text{(S3.1.5)}$$

$$\langle G \rangle \approx f_D c_{G,D} G_{noon} + (1 - f_D) c_{G,N} G_{noon,NE}, \quad \text{(S3.1.6)}$$

where $R_{d.noon,NE}$ and $G_{noon,NE}$ are the leaf dark respiration, R_d, and growth, G, respectively, evaluated at noon if there were no transpiration and thus if turgor was maximal (subscript "*NE*" for "no transpiration"; assuming transpiration rates are small at night), f_D is the fraction of the day (a day being 24 hours) over which photosynthesis occurs (estimated as the fraction of the day over which incoming irradiance is positive like in Section S2), f_A is a unitless factor that relates noon A_n to the average net photosynthetic carbon assimilation rate over the period of day with positive irradiance (set to 0.5, assuming an approximately triangular diurnal profile of A_n), $c_{R,D}$ and $c_{R,N}$ are daytime and nighttime factors that correct for differences between the instantaneous noon maintenance respiration rate and their average values estimated for daytime and nighttime temperature variations, respectively,

$$c_{R,D} = \frac{\int^{Day} R_M(T_a(t))\, dt}{R_{M,noon} \int^{Day} dt} \quad \text{(S3.1.7)}$$

$$c_{R,N} = \frac{\int^{Night} R_M(T_a(t))\, dt}{R_{M,noon} \int^{Night} dt}, \quad \text{(S3.1.8)}$$

and $c_{G,D}$ and $c_{G,N}$ are daytime and nighttime factors that correct for differences between the instantaneous noon growth rate and their average values estimated for daytime and nighttime temperature variations, respectively, which we approximated solely through the temperature-dependence of the extensibility, $\tilde{\phi}$,

$$c_{G,D} = \frac{\int^{Day} \tilde{\phi}(T_a(t))\, dt}{\tilde{\phi}_{noon} \int^{Day} dt} \quad \text{(S3.1.9)}$$

$$c_{G,N} = \frac{\int^{Night} \tilde{\phi}(T_a(t))\, dt}{\tilde{\phi}_{noon} \int^{Night} dt}. \quad \text{(S3.1.10)}$$

This approximation for $\langle A_n \rangle$, $\langle R_M \rangle$, and $\langle G \rangle$ (Eq. 3.1.4-6) enables us to estimate $\bar{C}$ solely from instantaneous noon physiological variables and diel variations in temperature without calculating physiological variables at multiple times throughout a DOY.

S4 Probabilistic Growth Maximization

S4.1 General framework for probabilistic growth maximization

Suppose the stomata of an individual tree maximizes whole-stem growth in a probabilistic way over the tree's lifetime. It is probabilistic, because the stomata do not '*know*' the exact environmental conditions that will occur in the near future. Similarly, the stomata of leaves cannot directly '*know*' the exact conditions that the rest of the tree is experiencing (e.g., stem, roots), since plants are modular. For example, the stomata respond to their own leaf temperature (T_L); however, stomata likely are not able to directly respond to the temperature of the rest of the aboveground portion of the plant (e.g., stems), which regulates stem growth and respiration, and which we here approximate by the air temperature (T_a), though in truth, stem temperatures are neither uniform within the stem nor fully coupled to air temperature (Stockfors, 2000). Indeed, T_L and T_a are decoupled, sometime by as much as ±20°C (Michaletz et al., 2016), by solar radiation absorbed by the lead and by evaporative cooling. At best, plants might retain a statistical '*memory*' through natural selection about the probability distributions of environmental conditions (Buckley et al., 2017), from which stomata might '*anticipate*' a range of environmental conditions in a probabilistic manner for both themselves (i.e., leaf conditions) and the rest of the plant (e.g., stems). Additionally, studies of circadian regulation of nocturnal stomatal behaviour over diel periods (Hennessey et al., 1993; Resco de Dios et al., 2012, 2020) and of phenological regulation of stomata over seasons (Lauriks et al., 2021, 2022) suggest that plant '*memory*' might distinguish between conditions experienced at different times of day and times of year (Borchert, 1994; Vico et al., 2015), such that the '*anticipated*' probability distributions might vary over diel and seasonal cycles. Mathematically, this probabilistic growth maximization is stated as

$$\max_{g_w} \int \langle \hat{G} \rangle (C, E, t)\ dt \tag{S4.1.1}$$

where g_w is the optimal stomatal conductance, we use the $\langle x \rangle$ operator to denote an ensemble average and the circumflex ($\hat{x}$) to denote '*anticipated*' values, and $\langle \hat{G} \rangle$ is thus the '*anticipated*' ensemble growth rate, averaged over the probability distributions of all relevant environmental conditions at time, t,

$$\langle \hat{G} \rangle (C, E, t) = \int\int\int\int\int G(C, E) \cdot \hat{p}_{\mathrm{RH}}(\widehat{\mathrm{RH}}, t) \hat{p}_T(\hat{T}_a, t) \hat{p}_\psi(\hat{\psi}_{soil}, t) \hat{p}_I(\hat{I}_s, t) \hat{p}_\Phi(\hat{\Phi}, t)\ d\widehat{\mathrm{RH}}\ d\hat{T}_a\ d\hat{\psi}_{soil}\ d\hat{I}_s\ d\hat{\Phi} \tag{S4.1.2}$$

and where $\hat{p}_{\text{RH}}$, $\hat{p}_T$, $\hat{p}_\psi$, $\hat{p}_I$, and $\hat{p}_\Phi$ are the probability density functions at time, t, for the '*anticipated*' RH, T_a, ψ_{soil}, I_s, and Φ, respectively. The deterministic growth maximization presented by Potkay and Feng (2023; Eq. 1 in main text) is equivalent to this probabilistic growth maximization (Eq. S4.1.1-2) when $\hat{p}_{\text{RH}}$, $\hat{p}_T$, $\hat{p}_\psi$, $\hat{p}_I$, and $\hat{p}_\Phi$ are all described by zero variance (i.e., Dirac delta distributions) with means ($\langle\widehat{\text{RH}}\rangle$, $\langle\hat{T}_a\rangle$, $\langle\hat{\psi}_{soil}\rangle$, $\langle\hat{I}_s\rangle$, $\langle\hat{\Phi}\rangle$) equal to the actual conditions. Here, the variance is a measure of *precision* between '*anticipated*' and actual conditions with perfect *precision* at zero variance, and the difference between the mean '*anticipated*' and actual condition (i.e., $\langle\widehat{\text{RH}}\rangle$ - RH, $\langle\hat{T}_a\rangle$ - T_a, $\langle\hat{\psi}_{soil}\rangle$ - ψ_{soil}, $\langle\hat{I}_s\rangle$ - I_s, $\langle\hat{\Phi}\rangle$ - Φ) is a measure of *accuracy* with perfect *accuracy* at equality (i.e., $\langle\widehat{\text{RH}}\rangle$ = RH, $\langle\hat{T}_a\rangle = T_a$, $\langle\hat{\psi}_{soil}\rangle = \psi_{soil}$, $\langle\hat{I}_s\rangle = I_s$, $\langle\hat{\Phi}\rangle = \Phi$). As in Potkay and Feng (2023), this probabilistic growth maximization is constrained by the actual NSC reserve, C (rather than an '*anticipated*' NSC reserve, $\hat{C}$), since changes in C occur slowly and certainly much slower than changes in environmental conditions (RH, T_a, ψ_{soil}, I_s, and Φ). This constraint is defined by our equation for the whole-tree NSC balance (Eq. 2 in the main text). Given this constraint on the actual NSCs, we present below the general solution to this probabilistic growth maximization by following the same steps as Potkay and Feng (2023; see Section S2 in their SI).

First as in Potkay and Feng (2023), we write the augmented Lagrangian, using the notation of Witelski & Bowen (2015; see their Chapter 3.8), as

$$\mathcal{L}(E, C, \dot{C}, \eta, t) = \langle\hat{G}\rangle(C, E, t) - \eta\left[\dot{C} - F_C(E, C, t)\right], \quad \text{(S4.1.3)}$$

where η and F_C are arbitrary and undetermined variables. Please see Section S2 of Potkay and Feng's (2023) SI for further information on these variables. Second, from the *state equation* ($\partial\mathcal{L}/\partial\eta$ = 0), we identify an equivalence between $\dot{C}$ (given by Eq. 2) and F_C,

$$F_C(E, C, t) = \dot{C} = a_L A_n - R_M - \frac{G}{1-f_C}. \quad \text{(S4.1.4)}$$

Third, from the *control equation* ($\partial\mathcal{L}/\partial E$ = 0), we reach an equivalence for η,

$$\eta = -\frac{\frac{\partial\langle\hat{G}\rangle}{\partial E}}{\frac{\partial F_C}{\partial E}}, \quad \text{(S4.1.5)}$$

which, upon combination with Eq. S4.1.4 and simplification through equations in Potkay & Feng (2023; notably, $G = \sigma_g(C)\cdot G_0(E)$, and thus $\langle\hat{G}\rangle = \sigma_g(C)\cdot\langle\hat{G}_0\rangle(E)$, where G_0 is the sink-limited potential growth rate), becomes

$$\eta = -\frac{\frac{\partial\langle\hat{G}\rangle}{\partial E}}{a_L\chi_w - \frac{1}{1-f_c}\frac{\partial G}{\partial E}} = -\frac{\sigma_g(C)\frac{\partial\langle\hat{G}_0\rangle}{\partial E}}{a_L\chi_w - \frac{\sigma_g(C)}{1-f_c}\frac{\partial G_0}{\partial E}} = \frac{\sigma_g(C)\left|\frac{\partial\langle\hat{G}_0\rangle}{\partial E}\right|}{a_L\chi_w + \frac{\sigma_g(C)}{1-f_c}\left|\frac{\partial G_0}{\partial E}\right|}, \quad (S4.1.6)$$

where χ_w is the *marginal carbon cost of water*, which defines the solution for the optimal stomatal conductance, g_w, due to the equivalence of χ_w and the *marginal carbon profit of water*, λ (Eq. S1.3.10), at optimum (Potkay & Feng, 2023). Similar to Eq. S4.1.2,

$$\langle\hat{G}_0\rangle(E,t) = \int\int\int\int\int G_0(E)\cdot\hat{p}_{\mathrm{RH}}(\widehat{\mathrm{RH}},t)\hat{p}_T(\hat{T}_a,t)\hat{p}_\psi(\hat{\psi}_{soil},t)\hat{p}_I(\hat{I}_s,t)\hat{p}_\Phi(\hat{\Phi},t)\; d\widehat{\mathrm{RH}}\; d\hat{T}_a\; d\hat{\psi}_{soil}\; d\hat{I}_s\; d\hat{\Phi}. \quad (S4.1.7)$$

Lastly, we rearrange for the *marginal carbon cost of water*,

$$\chi_w = \frac{\sigma_g}{a_L}\left[\frac{1}{\eta}\left|\frac{\partial\langle\hat{G}_0\rangle}{\partial E}\right| - \frac{1}{1-f_c}\left|\frac{\partial G_0}{\partial E}\right|\right]. \quad (S4.1.8)$$

We note that Eq. S4.1.8 is similar to the solution of the deterministic growth maximization (Eq. 3 in the main text), and the two solutions are equal when $\partial\langle\hat{G}_0\rangle/\partial E = \partial G_0/\partial E$. Interestingly the general solution to the probabilistic growth maximization problem depends on both '*anticipated*' growth ($\eta^{-1}\cdot\partial\langle\hat{G}_0\rangle/\partial E$ terms in square-brackets in Eq. S4.1.8) and actual growth ($(1-f_c)^{-1}\cdot\partial G_0/\partial E$ terms). The former arises from the objective function (Eq. S4.1.1), while the latter originates from the constraint on growth owing from actual NSC-use (Eq. S4.1.4). Furthermore, since

$$\frac{\partial\langle\hat{G}_0\rangle}{\partial E} = \int\int\int\int\int \frac{\partial G_0}{\partial E}\cdot\hat{p}_{\mathrm{RH}}(\widehat{\mathrm{RH}},t)\hat{p}_T(\hat{T}_a,t)\hat{p}_\psi(\hat{\psi}_{soil},t)\hat{p}_I(\hat{I}_s,t)\hat{p}_\Phi(\hat{\Phi},t)\; d\widehat{\mathrm{RH}}\; d\hat{T}_a\; d\hat{\psi}_{soil}\; d\hat{I}_s\; d\hat{\Phi} \quad (S4.1.9)$$

by Eq. S4.1.7 and the Leibniz integral rule, and since $\partial G_0/\partial E$ depends on only RH, T_a, and ψ_{soil} (Potkay et al., 2022; Eq. S1.2.3), it emerges that the probabilistic optimization does not depend on plants' '*memory*' for light conditions (i.e., I_s, Φ) if any such '*memory*' were to exist. Only a '*memory*' for hydraulic and thermal conditions (RH, T_a, ψ_{soil}) would be relevant to the probabilistic optimization. Hence,

$$\frac{\partial\langle\hat{G}_0\rangle}{\partial E} = \int\int\int \frac{\partial G_0}{\partial E}\cdot\hat{p}_{\mathrm{RH}}(\widehat{\mathrm{RH}},t)\hat{p}_T(\hat{T}_a,t)\hat{p}_\psi(\hat{\psi}_{soil},t)\; d\widehat{\mathrm{RH}}\; d\hat{T}_a\; d\hat{\psi}_{soil}. \quad (S4.1.10)$$

S4.2 Nocturnal behaviour for probabilistic growth maximization with thermal 'memory'

Here, we derive an approximate solution for the 'fitness factor,' $f_f = \chi_{w,N}/\chi_{w,D}$ (Wang et al., 2021), where $\chi_{w,N}$ and $\chi_{w,D}$ are the *marginal carbon costs of water* at daytime and nighttime, respectively, following from our general solution for the stomatal conductance the maximizes growth in a probabilistic manner (Section S4.1; Eq. S4.1.8) and further assuming that plants

imperfectly '*anticipate*' a range of temperatures with their own probabilities at any given moment. That is, in this scenario, plants' '*anticipation*' of RH and ψ_{soil} is perfectly *accurate* (i.e., $\langle\widehat{\mathrm{RH}}\rangle$ = RH, $\langle\hat{\psi}_{soil}\rangle = \psi_{soil}$) and perfectly *precise* (i.e., $\hat{\sigma}_{\mathrm{RH}} = \hat{\sigma}_{\psi} = 0$, where $\hat{\sigma}_{\mathrm{RH}}$ and $\hat{\sigma}_{\psi}$ are the standard deviations for the probability distributions for '*anticipated*' RH and ψ_{soil}, $\hat{p}_{\mathrm{RH}}$ and $\hat{p}_{\psi}$, respectively), while plants' '*anticipation*' of T_a is imperfect in either or both of *precision* and *accuracy* (i.e., $|\langle\hat{T}_a\rangle - T_a| \geq 0$, $\hat{\sigma}_T \geq 0$, where $\hat{\sigma}_T$ is the standard deviations for the probability distribution for '*anticipated*' T_a, $\hat{p}_T$). Under these assumptions, Eq. S4.1.10 may be simplified to

$$\frac{\partial\langle\hat{G}_0\rangle}{\partial E} = \int \frac{\partial G_0}{\partial E} \cdot \hat{p}_T(\hat{T}_a, t)\, d\hat{T}_a. \quad \text{(S4.2.1)}$$

We have already derived an equation for $\partial G_0/\partial E$ (Eq. S1.2.3), in which $\partial G_0/\partial E$ depends on T_a through the '*effective*' extensibility, $\tilde{\phi}$ (see Potkay et al., 2022; Potkay & Feng, 2023), which is modeled as function of T_a (Cabon et al., 2020; Peters et al., 2021; Fig. S3 in Potkay & Feng, 2023), as well as through the water pressure potentials of the stem apex and root collar, ψ_S and ψ_{Rc}, respectively, since $\partial G_0/\partial E$ is defined in terms of $\partial\psi_S/\partial E$, $\partial\psi_{Rc}/\partial E$, $\partial\pi_0/\partial\psi_S$, and $\tilde{z}^+$, and ,which are all in turn dependent on ψ_S, ψ_{Rc}, or both, and since ψ_S and ψ_{Rc} depends on the hydraulic conductances (e.g., Eq. S1.1.7), which we modeled here as function of T_a (Matzner & Comstock, 2001; Wan et al. 2001; Sack et al., 2004; Section S1.1). However, if we ignore the dependences of hydraulic conductances on temperature on as a first-order approximation, then temperature affects $\partial G_0/\partial E$ through only $\tilde{\phi}(T_a)$. Hence, we rewrite the sink-limited potential growth rate as $G_0 = \tilde{\phi}(T_a)\cdot r_0$, and we approximate r_0 as independent of temperature, and thus $\partial G_0/\partial E \approx \tilde{\phi}(T_a)\cdot\partial r_0/\partial E$, where $\partial r_0/\partial E$ is also independent of temperature. Given this approximation, Eq. S4.2.1 may be rewritten as

$$\frac{\partial\langle\hat{G}_0\rangle}{\partial E} \approx \frac{\partial r}{\partial E}\int \tilde{\phi}(T_a) \cdot \hat{p}_T(\hat{T}_a, t)\, d\hat{T}_a = \langle\hat{\tilde{\phi}}\rangle(t)\frac{\partial r}{\partial E} = \frac{\langle\hat{\tilde{\phi}}\rangle(t)}{\tilde{\phi}(T_a)}\frac{\partial G_0}{\partial E}, \quad \text{(S4.2.2)}$$

where $\langle\hat{\tilde{\phi}}\rangle$ is the ensemble '*effective*' extensibility given the '*anticipated*' distribution of temperatures at time, t,

$$\langle\hat{\tilde{\phi}}\rangle(t) = \int \tilde{\phi}(T_a) \cdot \hat{p}_T(\hat{T}_a, t)\, d\hat{T}_a. \quad \text{(S4.2.3)}$$

Combining Eq. S4.1.8 and Eq. S4.2.2, the marginal carbon cost of water becomes

$$\chi_w \approx \frac{\sigma_g}{a_L}\left|\frac{\partial G_0}{\partial E}\right|\left[\frac{1}{\eta}\frac{\langle\hat{\tilde{\phi}}\rangle(t)}{\tilde{\phi}(T_a)} - \frac{1}{1-f_c}\right]. \quad \text{(S4.2.4)}$$

To describe the probability distribution of '*anticipated*' temperatures, we model $\hat{p}_T(\hat{T}_a, t)$ through a skew normally distribution (O'hagan & Leonard, 1976; Eq. S2.1.7), which is described by a mean, $\langle\hat{T}_a\rangle(t)$, a standard deviation, $\hat{\sigma_T}(t)$, and a skewness, $\hat{\alpha}(t)$, all of which may change in time, t, considering that stomatal behaviour changes over diel (Hennessey et al., 1993; Resco de Dios et al., 2012, 2020) and seasonal cycles (Lauriks et al., 2021, 2022). To simply describe the dynamics of the '*anticipated*' probability distributions, we approximate $\langle\hat{T}_a\rangle(t)$, $\hat{\sigma_T}(t)$, and $\hat{\alpha}(t)$ as square-wave functions in time over a diel cycle. That is, $\langle\hat{T}_a\rangle(t)$, $\hat{\sigma_T}(t)$, and $\hat{\alpha}(t)$ each have two values: a single daytime value and a single nighttime value. We denote their daytime values with D subscripts (e.g., $\langle\hat{T}_a\rangle_D$, $\hat{\sigma}_{T,D}$, $\hat{\alpha}_D$) and their nighttime values with N subscripts (e.g. $\langle\hat{T}_a\rangle_N$, $\hat{\sigma}_{T,N}$, $\hat{\alpha}_N$). Given that $\partial G_0/\partial E \propto \tilde{\phi}(T_a)$, we apply Eq. S4.2.4 to write Wang et al.'s (2021) 'fitness factor' as

$$f_f = \frac{\chi_{w,N}}{\chi_{w,D}} \approx \frac{\sigma_g(C_N)\tilde{\phi}(T_{a,N})\left[\frac{1}{\eta_N}\frac{\langle\tilde{\hat{\phi}}\rangle_N}{\tilde{\phi}(T_{a,N})} - \frac{1}{1-f_c}\right]}{\sigma_g(C_D)\tilde{\phi}(T_{a,D})\left[\frac{1}{\eta_D}\frac{\langle\tilde{\hat{\phi}}\rangle_D}{\tilde{\phi}(T_{a,D})} - \frac{1}{1-f_c}\right]} = \frac{\sigma_g(C_N)\left[\frac{\langle\tilde{\hat{\phi}}\rangle_N}{\eta_N} - \frac{\tilde{\phi}(T_{a,N})}{1-f_c}\right]}{\sigma_g(C_D)\left[\frac{\langle\tilde{\hat{\phi}}\rangle_D}{\eta_D} - \frac{\tilde{\phi}(T_{a,D})}{1-f_c}\right]}. \qquad \text{(S3.2.5)}$$

Since changes in C and η are expected to be small between daytime and nighttime ($C_D \approx C_N$; $\eta_D \approx \eta_N \approx \eta$) according to our simulations (Fig. 3-5&S8-S9), Eq. S4.2.5 can be further approximated as

$$f_f \approx \frac{\frac{\langle\tilde{\hat{\phi}}\rangle_N}{\eta} - \frac{\tilde{\phi}(T_{a,N})}{1-f_c}}{\frac{\langle\tilde{\hat{\phi}}\rangle_D}{\eta} - \frac{\tilde{\phi}(T_{a,D})}{1-f_c}}. \qquad \text{(S3.2.6)}$$

References

Bartlett, M. K., Detto, M., & Pacala, S. W. (2019). Predicting shifts in the functional composition of tropical forests under increased drought and CO_2 from trade-offs among plant hydraulic traits. *Ecology Letters*, *22*(1), 67-77.

Bartlett, M. K., Scoffoni, C., & Sack, L. (2012). The determinants of leaf turgor loss point and prediction of drought tolerance of species and biomes: a global meta-analysis. *Ecology letters*, *15*(5), 393-405.

Borchert, R. (1994). Soil and stem water storage determine phenology and distribution of tropical dry forest trees. *Ecology*, *75*(5), 1437-1449.

Brienen, R., Helle, G., Pons, T., Boom, A., Gloor, M., Groenendijk, P., ... & Jones, C. (2022). Paired analysis of tree ring width and carbon isotopes indicates when controls on tropical tree growth change from light to water limitations. *Tree Physiology*, *42*(6), 1131-1148.

Buckley, T. N., Miller, J. M., & Farquhar, G. D. (2002). The mathematics of linked optimisation for water and nitrogen use in a canopy. *Silva Fennica*, *36*(3), 639-669.

Buckley, T. N., Sack, L., & Farquhar, G. D. (2017). Optimal plant water economy. *Plant, cell & environment*, *40*(6), 881-896.

Cabon, A., Peters, R. L., Fonti, P., Martínez-Vilalta, J., & De Cáceres, M. (2020). Temperature and water potential co-limit stem cambial activity along a steep elevational gradient. *New Phytologist*, *226*(5), 1325-1340.

Caird, M. A., Richards, J. H., & Donovan, L. A. (2007). Nighttime stomatal conductance and transpiration in C3 and C4 plants. *Plant physiology*, *143*(1), 4-10.

Cochard, H., Martin, R., Gross, P., & Bogeat-Triboulot, M. B. (2000). Temperature effects on hydraulic conductance and water relations of *Quercus robur* L. *Journal of Experimental Botany*, *51*(348), 1255-1259.

Collalti, A., Ibrom, A., Stockmarr, A., Cescatti, A., Alkama, R., Fernández-Martínez, M., ... & Prentice, I. C. (2020). Forest production efficiency increases with growth temperature. *Nature communications*, *11*(1), 1-9.

Dewar, R., Mauranen, A., Mäkelä, A., Hölttä, T., Medlyn, B., & Vesala, T. (2018). New insights into the covariation of stomatal, mesophyll and hydraulic conductances from optimization models incorporating nonstomatal limitations to photosynthesis. *New Phytologist*, *217*(2), 571-585.

Flexas, J., Galmes, J., Ribas-Carbo, M., & Medrano, H. (2005). The Effects of Water Stress on Plant Respiration. In Lambers, H., & Ribas-Carbo, M. (Eds.), *Plant Respiration* (pp. 85-94). Springer. https://doi.org/10.1007/1-4020-3589-6_6.

Galmés, J., Ribas-Carbó, M., Medrano, H., & Flexas, J. (2007). Response of leaf respiration to water stress in Mediterranean species with different growth forms. *Journal of Arid Environments*, *68*(2), 206-222.

Hennessey, T. L., Freeden, A. L., & Field, C. B. (1993). Environmental effects on circadian rhythms in photosynthesis and stomatal opening. *Planta*, *189*(3), 369-376.

Huang, H., Ran, J., Ji, M., Wang, Z., Dong, L., Hu, W., ... & Deng, J. (2020). Water content quantitatively affects metabolic rates over the course of plant ontogeny. *New Phytologist*, *228*(5), 1524-1534.

Jones, S., Rowland, L., Cox, P., Hemming, D., Wiltshire, A., Williams, K., ... & Harper, A. B. (2020). The impact of a simple representation of non-structural carbohydrates on the simulated response of tropical forests to drought. *Biogeosciences*, *17*(13), 3589-3612.

Kalnay E, Kanamitsu M, Kistler R, Collins W, Deaven D, Gandin L, Zhu Y. (1996). The NCEP/NCAR 40-year reanalysis project. *Bull Am Meteorol Soc 77*:437–472.

Lauriks, F., Salomón, R. L., De Roo, L., & Steppe, K. (2021). Leaf and tree responses of young European aspen trees to elevated atmospheric CO_2 concentration vary over the season. *Tree Physiology*, *41*(10), 1877-1892.

Lauriks, F., Salomón, R. L., De Roo, L., Sobrino-Plata, J., Rodríguez-García, A., & Steppe, K. (2022). Limited mitigating effects of elevated CO_2 in young aspen trees to face drought stress. *Environmental and Experimental Botany*, 104942.

Lembrechts, J. J., Van den Hoogen, J., Aalto, J., Ashcroft, M. B., De Frenne, P., Kemppinen, J., ... & Hik, D. S. (2022). Global maps of soil temperature. *Global Change Biology, 28*(9), 3110-3144.

Lintunen, A., Paljakka, T., Salmon, Y., Dewar, R., Riikonen, A., & Hölttä, T. (2020). The influence of soil temperature and water content on belowground hydraulic conductance and leaf gas exchange in mature trees of three boreal species. *Plant, Cell & Environment, 43*(3), 532-547.

Manzoni, S., Vico, G., Palmroth, S., Porporato, A., & Katul, G. (2013). Optimization of stomatal conductance for maximum carbon gain under dynamic soil moisture. *Advances in Water Resources*, *62,* 90-105.

Matzner, S., & Comstock, J. (2001). The temperature dependence of shoot hydraulic resistance: implications for stomatal behaviour and hydraulic limitation. *Plant, Cell & Environment, 24*(12), 1299-1307.

Michaletz, S. T., Weiser, M. D., McDowell, N. G., Zhou, J., Kaspari, M., Helliker, B. R., & Enquist, B. J. (2016). The energetic and carbon economic origins of leaf thermoregulation. *Nature plants*, *2*(9), 1-9.

Mrad, A., Sevanto, S., Domec, J. C., Liu, Y., Nakad, M., & Katul, G. (2019). A dynamic optimality principle for water use strategies explains isohydric to anisohydric plant responses to drought. *Frontiers in Forests and Global Change*, *2,* 49.

Nadal-Sala, D., Grote, R., Birami, B., Knüver, T., Rehschuh, R., Schwarz, S., & Ruehr, N. K. (2021). Leaf shedding and non-stomatal limitations of photosynthesis mitigate hydraulic conductance losses in scots pine saplings during severe drought stress. *Frontiers in plant science*, 1600.

Novick, K. A., Miniat, C. F., & Vose, J. M. (2016). Drought limitations to leaf-level gas exchange: results from a model linking stomatal optimization and cohesion–tension theory. *Plant, cell & environment,* 39(3), 583-596.

O'hagan, A., & Leonard, T. (1976). Bayes estimation subject to uncertainty about parameter constraints. *Biometrika*, *63*(1), 201-203.

Peters, R. L., Steppe, K., Cuny, H. E., De Pauw, D. J., Frank, D. C., Schaub, M., ... & Fonti, P. (2021). Turgor–a limiting factor for radial growth in mature conifers along an elevational gradient. *New Phytologist*, *229*(1), 213-229.

Potkay, A., & Feng, X. (2023). Do stomata optimize turgor-driven growth?: A new framework for integrating stomata response with whole-plant hydraulics and carbon balance. *New Phytologist*, https://doi.org/10.1111/nph.18620.

Potkay, A., Hölttä, T., Trugman, A. T., & Fan, Y. (2022). Turgor-limited predictions of tree growth, height and metabolic scaling over tree lifespans. *Tree Physiology*, 42(2), 229-252.

Resco de Dios, V., Anderegg, W. R., Li, X., Tissue, D. T., Bahn, M., Landais, D., ... & Gessler, A. (2020). Circadian regulation does not optimize stomatal behaviour. *Plants*, *9*(9), 1091.

Resco de Dios, V., Goulden, M. L., Ogle, K., Richardson, A. D., Hollinger, D. Y., Davidson, E. A., ... & Moreno, J. M. (2012). Endogenous circadian regulation of carbon dioxide exchange in terrestrial ecosystems. *Global Change Biology*, *18*(6), 1956-1970.

Ryan, M. G., Phillips, N., & Bond, B. J. (2006). The hydraulic limitation hypothesis revisited. *Plant, Cell & Environment*, *29*(3), 367-381.

Sack, L., Streeter, C. M., & Holbrook, N. M. (2004). Hydraulic analysis of water flow through leaves of sugar maple and red oak. *Plant Physiology*, *134*(4), 1824-1833.

Saveyn, A., Steppe, K., & Lemeur, R. (2007). Daytime depression in tree stem CO_2 efflux rates: is it caused by low stem turgor pressure?. *Annals of Botany*, *99*(3), 477-485.

Schiestl-Aalto, P., Ryhti, K., Mäkelä, A., Peltoniemi, M., Bäck, J., & Kulmala, L. (2019). Analysis of the NSC storage dynamics in tree organs reveals the allocation to belowground symbionts in the framework of whole tree carbon balance. *Frontiers in Forests and Global Change*, *2*, 17.

Stockfors, J. (2000). Temperature variations and distribution of living cells within tree stems: implications for stem respiration modeling and scale-up. *Tree Physiology*, *20*(15), 1057-1062.

Tuzet, A., Perrier, A., & Leuning, R. (2003). A coupled model of stomatal conductance, photosynthesis and transpiration. *Plant, Cell & Environment*, *26*(7), 1097-1116.

Vico, G., Thompson, S. E., Manzoni, S., Molini, A., Albertson, J. D., Almeida-Cortez, J. S., ... & Porporato, A. (2015). Climatic, ecophysiological, and phenological controls on plant ecohydrological strategies in seasonally dry ecosystems. *Ecohydrology*, *8*(4), 660-681.

Wan, X., Zwiazek, J. J., Lieffers, V. J., & Landhäusser, S. M. (2001). Hydraulic conductance in aspen (*Populus tremuloides*) seedlings exposed to low root temperatures. *Tree Physiology*, *21*(10), 691-696.

Wang, Y., Anderegg, W. R., Venturas, M. D., Trugman, A. T., Yu, K., & Frankenberg, C. (2021). Optimization theory explains nighttime stomatal responses. *New Phytologist*, *230*(4), 1550-1561.

Waring, R. H., Landsberg, J. J., & Williams, M. (1998). Net primary production of forests: a constant fraction of gross primary production?. *Tree physiology*, *18*(2), 129-134.

Zhou, S., Duursma, R. A., Medlyn, B. E., Kelly, J. W., & Prentice, I. C. (2013). How should we model plant responses to drought? An analysis of stomatal and non-stomatal responses to water stress. *Agricultural and Forest Meteorology*, *182*, 204-214.
